# Oligonucleotides Featuring a Covalently Mercurated 6‐Phenylcarbazole Residue as High‐Affinity Hybridization Probes for Thiopyrimidine‐Containing Sequences

**DOI:** 10.1002/chem.202202530

**Published:** 2022-10-19

**Authors:** Tharun K. Kotammagari, Petri Tähtinen, Tuomas Lönnberg

**Affiliations:** ^1^ Department of Chemistry University of Turku Henrikinkatu 2 20500 Turku Finland

**Keywords:** base pairs, hybridization, mercury, oligonucleotides, thionucleosides

## Abstract

Short oligonucleotides incorporating either 1‐mercuri‐6‐phenylcarbazole, 8‐mercuri‐6‐phenylcarbazole, or 1,8‐dimercuri‐6‐phenylcarbazole C‐nucleoside in the middle of the chain have been synthesized and studied for their potential as hybridization probes for sequences containing thiopyrimidine nucleobases. All of these oligonucleotides formed very stable duplexes with complementary sequences pairing the organometallic moiety with either 2‐ or 4‐thiothymine. The isomeric monomercurated oligonucleotides were also able to discriminate between 2‐ and 4‐thiothymine based on the different melting temperatures of the respective duplexes. DFT‐optimized structures of the most stable mononuclear Hg^II^‐mediated base pairs featured a coordinated covalent bond between Hg^II^ and either S2 or S4 and a hydrogen bond between the carbazole nitrogen and N3. The dinuclear Hg^II^‐mediated base pairs, in turn, were geometrically very similar to the one previously reported to form between 1,8‐dimercuri‐6‐phenylcarbazole and thymine and had one Hg^II^ ion coordinated to a thio and the other one to an oxo substituent.

## Graphical abstract

**Getting back to (nucleo)base**: Oligonucleotides incorporating a central 1‐ or 8‐mercuri‐6‐phenyl‐1*H*‐carbazole residue show great promise as high‐affinity hybridization probes, discriminating between all canonical nucleobases as well as 2‐ and 4‐thiothymines by melting temperature margins of at least 5 °C. Duplexes facilitating Hg^II^−S coordination were particularly stable, with melting temperatures nearly 30 °C higher than those of respective unmodified duplexes.

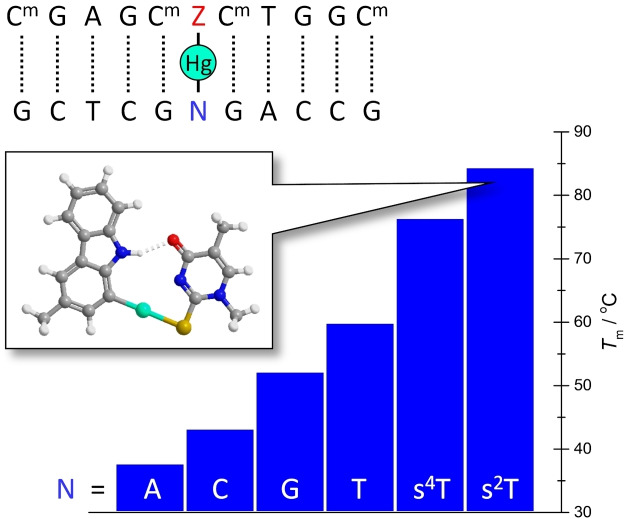

## Introduction

Thionucleosides are frequently found in the tRNAs of all types of organisms, playing various roles from structural stabilization to codon recognition.[^1^, ^2^, ^3^, ^4^, ^5^, ^6^, ^7^, ^8^, ^9^, ^10^] Thio modifications – or lack thereof – especially within the anticodon loop are associated with a number of diseases,[^11^, ^12^, ^13^, ^14^, ^15^, ^16^, ^17^, ^18^, ^19^] making thiolated tRNA an interesting diagnostic target.

**Figure 1:**
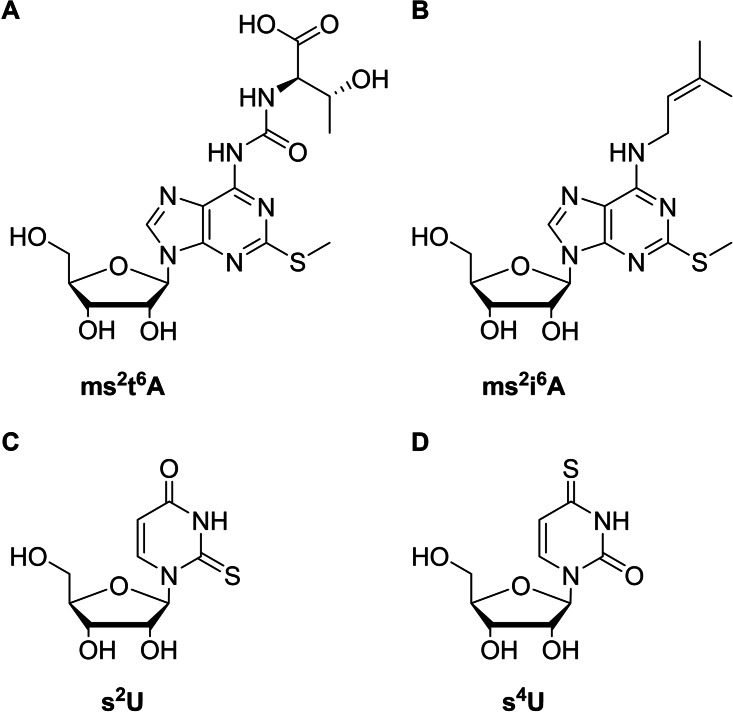
Examples of thionucleosides found in tRNA. A) 2‐Methylthio‐*N*6‐threonylcarbamoyladenosine, B) 2‐methylthio‐*N*6‐isopentenyladenosine, C) 2‐thiouridine, and D) 4‐thiouridine.

[(*N*‐Acryloylamino)phenyl]mercuric chloride (APM) gel electrophoresis[^20^, ^21^] remains one of the most popular methods for the enrichment and detection of thiolated RNA.[^22^, ^23^, ^24^] The method is based on the very high affinity of sulfur for mercury, causing slower migration of sulfur‐containing compounds. This retardation is highly dependent on the type of the thio modification but much less so on its position. For example, thioether nucleosides, such as 2‐methylthio‐*N*6‐threonylcarbamoyladenosine (Figure 1A, ms^2^t^6^A) and 2‐methylthio‐*N*6‐isopentenyladenosine (Figure 1B, ms^2^i^6^A), pass through readily, whereas thioketone nucleosides, such as 2‐thiouridine (Figure 1C, s^2^U) and 4‐thiouridine (Figure 1D, s^4^U) are retained strongly.^[25]^

**Figure 2:**
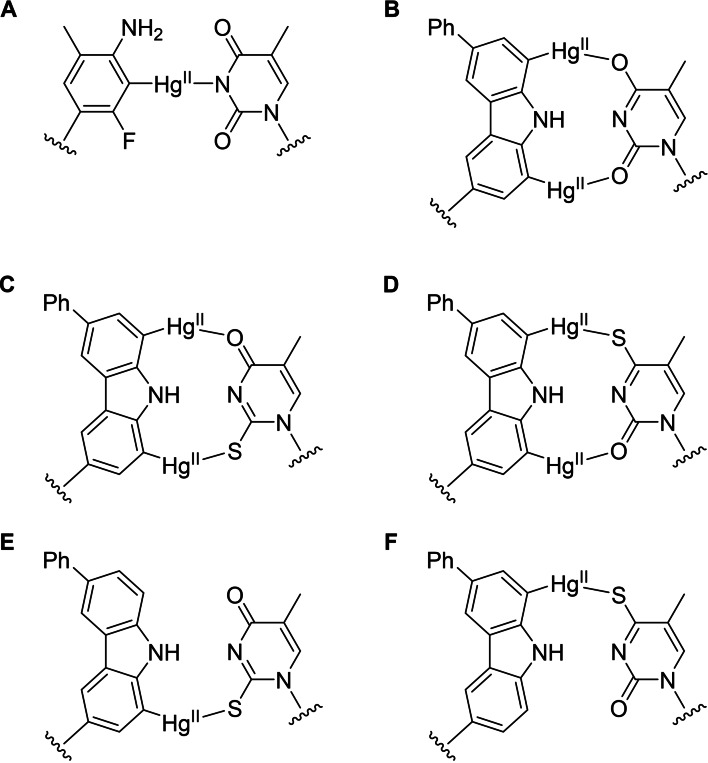
Hg^II^‐mediated base pairing between artificial organomercury nucleobase surrogates and naturally occurring thymine, 2‐thiothymine and 4‐thiothymine nucleobases. A) 3‐Fluoro‐2‐mercuri‐6‐methylaniline‐thymine, B) 1,8‐dimercuri‐6‐phenylcarbazole‐thymine, C) 1,8‐dimercuri‐6‐phenylcarbazole‐2‐thiothymine, D) 1,8‐dimercuri‐6‐phenylcarbazole‐4‐thiothymine, E) 1‐mercuri‐6‐phenylcarbazole‐2‐thiothymine and F) 8‐mercuri‐6‐phenylcarbazole‐4‐thiothymine.

Over the past six years, we have been studying oligonucleotides incorporating organomercury nucleobase surrogates for their potential as hybridization probes.[^26^, ^27^] Structures facilitating coordination of the Hg^II^ ion to pyrimidine‐N3 or purine‐N1 within a complementary strand typically lead to duplex (or triplex) stabilization and sometimes to improved discrimination of the canonical nucleobase (Figure 2A).[^28^, ^29^, ^30^, ^31^] We have also tested the opposite strategy, namely simultaneous coordination of the two Hg^II^ ions of 1,8‐dimercuri‐6‐phenylcarbazole to thymine‐O2 and O4 (Figure 2B). This unusual binding mode was supported by DFT calculations carried out at the PBE0DH level of theory but the observed duplex stabilization was actually somewhat lower than with mononuclear coordination of Hg^II^ to thymine‐N3.^[32]^

While affinity of 1,8‐dimercuri‐6‐phenylcarbazole for the four canonical nucleobases left something to be desired, one could expect this organomercury nucleobase analogue to be well suited for pairing with thionucleobases, especially 2‐ and 4‐thiopyrimidines (Figure 2C and D). For reference, dinuclear Ag(I)‐mediated homo base pairs of 2‐ and 4‐thiothymine have been shown to stabilize oligonucleotide duplexes much more than canonical Watson‐Crick base pairs.^[33]^ Furthermore, the monomercurated analogues 1‐mercuri‐6‐phenylcarbazole and 8‐mercuri‐6‐phenylcarbazole should be able to discriminate between 2‐ and 4‐thiopyrimidines (Figure 2E and F). In this article we present the synthesis of short oligonucleotides incorporating either of the monomercurated carbazole residues in the middle of the sequence and demonstrate their potential as hybridization probes for nucleic acids containing 2‐ or 4‐thiopyrimidine modifications. Like APM gel electrophoresis, our method harnesses the high affinity between mercury and sulfur but has the distinct advantage of also making use of sequence information.

## Results and Discussion

### Oligonucleotide synthesis

**Scheme 1:**
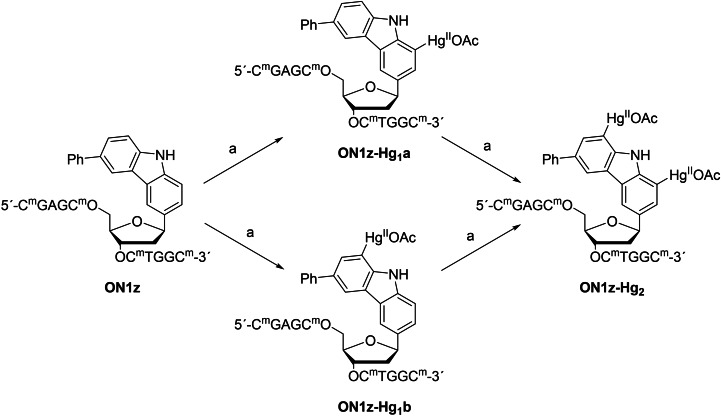
Mercuration of oligonucleotide **ON1z**. a) Hg(OAc)_2_, NaOAc, H_2_O, 55 °C, 12 h.

To facilitate comparison of the results with those of our earlier studies,[^28^, ^30^, ^31^, ^32^] the same oligonucleotide sequences were used (Table 1). Preparation of **ON1z‐Hg_2_**, incorporating a 1,8‐dimercuri‐6‐phenylcarbazole C‐nucleoside in the middle of the chain, by post‐synthetic mercuration of the respective unmetallated oligonucleotide **ON1z** has been reported previously.^[32]^ Two of the carbon atoms of carbazole are ortho and two para to the activating NH group and, hence, prone to mercuration. In the 6‐phenylcarbazole C‐nucleoside, however, the para positions are blocked by the phenyl and the sugar substituents. The two ortho positions are mercurated at comparable rates so we reasoned that incomplete mercuration of **ON1z** should afford a mixture of the dimercurated product **ON1z‐Hg_2_** and the monomercurated intermediates **ON1z‐Hg_1_a** and **ON1z‐Hg_1_b** (Scheme 1). In the flanking sequences 5‐methylcytosines were used instead of cytosines to prevent off‐target mercuration.

**Table 1:** Sequences of the oligonucleotides used in this study.

Oligonucleotide	Sequence^[a]^
**ON1a**	5′‐d(CGAGCACTGGC)‐3′
**ON1z**	5′‐d(C^m^GAGC^m^ ZC^m^TGGC^m^)‐3′
**ON1z‐Hg_1_a**	5′‐d(C^m^GAGC^m^ Z ^Hg1a^C^m^TGGC^m^)‐3′
**ON1z‐Hg_1_b**	5′‐d(C^m^GAGC^m^ Z ^Hg1b^C^m^TGGC^m^)‐3′
**ON1z‐Hg_2_**	5′‐d(C^m^GAGC^m^ Z ^Hg2^C^m^TGGC^m^)‐3′
**ON2a**	5′‐d(GCCAGAGCTCG)‐3′
**ON2c**	5′‐d(GCCAGCGCTCG)‐3′
**ON2g**	5′‐d(GCCAGGGCTCG)‐3′
**ON2t**	5′‐d(GCCAGTGCTCG)‐3′
**ON2s^2^t**	5′‐d(GCCAGs ^2^ TGCTCG)‐3′
**ON2s^4^t**	5′‐d(GCCAGs ^4^ TGCTCG)‐3′

[a] C^m^ refers to 5‐methylcytosine, Z to 6‐phenylcarbazole, Z^Hg1a^ to 1‐mercuri‐6‐phenylcarbazole, Z^Hg1b^ to 8‐mercuri‐6‐phenylcarbazole, Z^Hg2^ to 1,8‐dimercuri‐6‐phenylcarbazole, s^2^T to 2‐thiothymine and s^4^T to 4‐thiothymine. In each sequence, the residue varied in the hybridization experiments has been underlined.

Mercuration of **ON1z** (30 nmol) was carried out by treatment with an aqueous solution of mercuric acetate (10 μmol) and sodium acetate (20 μmol) at 55 °C. After 12 h, saturated aqueous solution of sodium chloride was added to precipitate out excess Hg^II^, effectively quenching the reaction. The product mixture was fractioned by RP‐HPLC with elution buffers containing 10 mM ethanethiol to suppress ligand exchange at Hg^II^. Four well‐resolved major peaks were observed. According to ESI‐TOF‐MS analysis, the first eluting peak contained the unreacted starting material **ON1z**, the second and third eluting peaks the monomercurated intermediates **ON1z‐Hg_1_a** and **ON1z‐Hg_1_b** and the fourth eluting peak the dimercurated product **ON1z‐Hg_2_**. We have previously shown by enzymatic digestion that with the final product **ON1z‐Hg_2_** mercuration is strictly limited to the 6‐phenylcarbazole residue^[32]^ and undoubtedly this is the case also for the monomercurated intermediates. Unfortunately, the site of mercuration of the monomercurated intermediates could not be assigned unambiguously but hybridization properties of these oligonucleotides (see below) strongly suggest that the faster eluting one (**ON1z‐Hg_1_a**) was mercurated at C1 and the slower eluting one (**ON1z‐Hg_1_b**) at C8 of the carbazole ring.

### Hybridization studies

Stabilities of duplexes formed by the modified oligonucleotides **ON1z**, **ON1z‐Hg_1_a**, **ON1z‐Hg_1_b** and **ON1z‐Hg_2_** with their counterparts **ON2a**, **ON2c**, **ON2g**, **ON2t**, **ON2s^2^t** and **ON2s^4^t** were determined by conventional UV melting temperature (*T*_m_) measurements. In each of the duplexes, one modified nucleobase surrogate was paired with one natural nucleobase, including the rare nucleobases 2‐thiothymine and 4‐thiothymine. Duplexes formed by **ON1a**, with otherwise identical sequence but replacing the modified nucleobase surrogate with adenine, were studied for reference. The experiments were carried out under otherwise the same conditions used previously^[32]^ with **ON1z** and **ON1z‐Hg_2_** (pH 7.4, 20 mM cacodylate buffer, *I*=0.10 M) but the ionic strength was adjusted with sodium chloride rather than sodium perchlorate to rule out oxidation of the thioketone functions.

**Figure 3:**
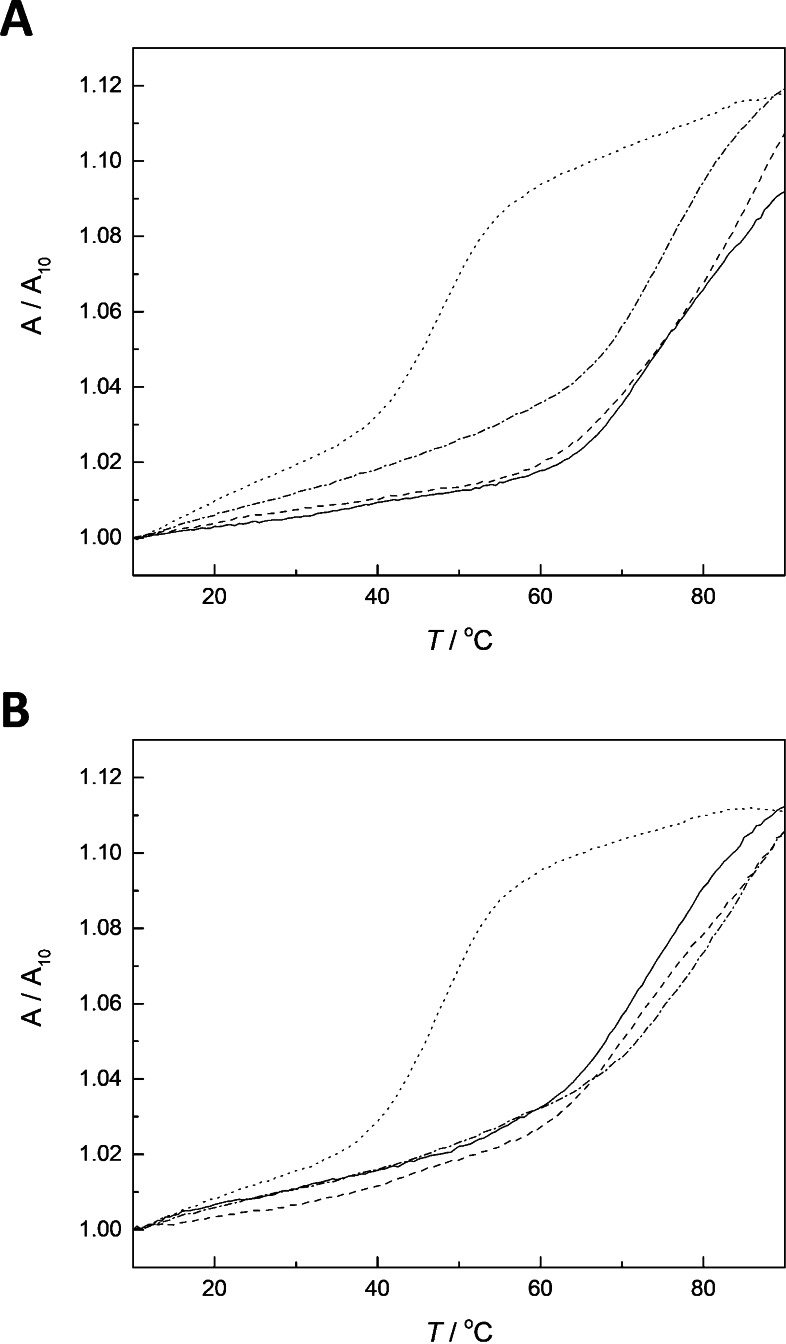
UV melting profiles of duplexes between oligonucleotides A) **ON2s^2^t** or B) **ON2s^4^t** and **ON1z** (dotted line), **ON1z‐Hg_1_a** (dashed line), **ON1z‐Hg_1_b** (dot‐dashed line) and **ON1z‐Hg_2_** (solid line); pH 7.4 (20 mM cacodylate buffer); [oligonucleotides]=1.0 μM; *I*(NaCl)=0.10 M.

**Figure 4:**
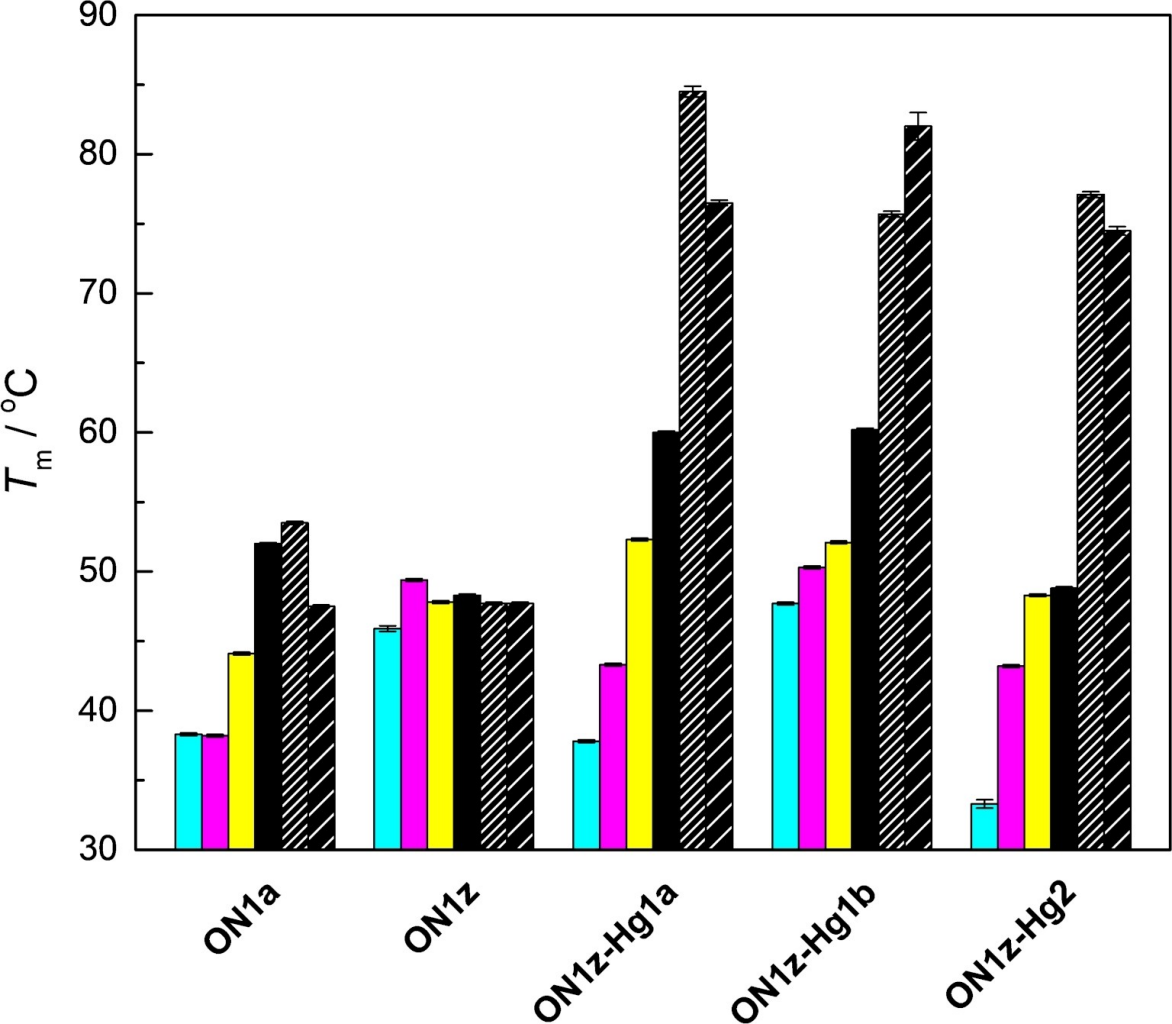
Melting temperatures of duplexes formed by the modified oligonucleotides **ON1a**, **ON1z**, **ON1z‐Hg_1_a**, **ON1z‐Hg_1_b** and **ON1z‐Hg_2_** with the natural oligonucleotides **ON2a** (cyan), **ON2c** (magenta), **ON2g** (yellow), **ON2t** (black), **ON2s^2^t** (black with dense hash), and **ON2s^4^t** (black with light hash); pH 7.4 (20 mM cacodylate buffer); [oligonucleotides]=1.0 μM; *I*(NaCl)=0.10 M. The error bars represent standard errors of the average of three experiments.

Figure 3 shows UV melting profiles of duplexes formed by the modified oligonucleotides with the 2‐thiothymine‐ and 4‐thiothymine‐containing oligonucleotides **ON2s^2^t** and **ON2s^4^t** (all melting profiles are provided in Figures S6–S35 in the Supporting Information). Monophasic sigmoidal curves were observed in nearly all cases, although with the most stable duplexes the curves did not level off even at the high end of the temperature range. The most notable exception was duplex **ON1z‐Hg_2_⋅ON2t**, the melting of which was biphasic (Figure S33). In line with previous reports,[^28^, ^30^, ^34^, ^35^, ^36^] the melting curves of duplexes incorporating a Hg^II^‐mediated base pair were broad. Melting temperatures of all duplexes studied are summarized in Figure 4 and tabulated in numerical form in Table S1.

Melting temperatures of duplexes formed by the unmodified oligonucleotide **ON1a** revealed the expected pattern of low stability (*T*_m_=38.2–44.1 °C) of the mispairs and a much higher (*T*_m_=52.0 °C) stability of the canonical A**⋅**T base pair. The base pair formed with 2‐thiothymine was slightly more stable than the A**⋅**T base pair whereas the one formed with 4‐thiothymine was considerably less stable, in line with sulfur being sterically bulkier and a weaker hydrogen bond acceptor than oxygen. Similar pseudocomplementarity of base pairing has been reported previously with 2,6‐diaminopurine and 2‐thiouracil.^[37]^

Melting temperatures of duplexes formed by the modified but unmercurated oligonucleotide **ON1z** were similar to those obtained previously^[32]^ with sodium perchlorate as the background electrolyte, ranging from 45.9 to 49.4 °C. Differences between duplexes placing different natural nucleobases opposite to the 6‐phenylcarbazole residue were small and in the case of thymine, 2‐thiothymine and 4‐thiothymine almost nonexistent. Accordingly, the relatively high thermal stabilities (compared to the mismatched unmodified duplexes **ON1a⋅ON2a**, **ON1a⋅ON2c** and **ON1a⋅ON2g**) probably stem from enhanced base stacking and hydrophobic interactions of the 6‐phenylcarbazole moiety and the methyl substituents of the cytosine bases, rather than base pairing.

In striking contrast to its unmercurated counterpart, hybridization affinity of the faster eluting monomercurated oligonucleotide **ON1z‐Hg_1_a** was highly dependent on the variable nucleobase of the complementary strand. Melting temperatures of duplexes pairing the mercurated carbazole residue with a canonical nucleobase ranged from 37.8 to 60.0 °C and were separated from one another by a margin of at least 5.5 °C. As expected, the thiothymine‐containing duplexes were much more stable, the melting temperatures of **ON1z‐Hg_1_a⋅ON2s^2^t** and **ON1z‐Hg_1_a⋅ON2s^4^t** being 84.5 and 76.5 °C, respectively. The higher affinity for **ON2s^2^t** than for **ON2s^4^t** suggests that the 6‐phenylcarbazole residue of **ON1z‐Hg_1_a** is mercurated at C1, resulting in nearly optimal geometry for Hg^II^‐mediated base pairing with 2‐thiothymine (Figure 2E).

The slower eluting monomercurated oligonucleotide **ON1z‐Hg_1_b** showed similar hybridization preferences for **ON2a**, **ON2c**, **ON2g** and **ON2t** as its faster eluting counterpart. Affinities for **ON2c** and especially **ON2a**, however, were markedly higher, translating into poorer overall discrimination between the four canonical nucleobases. Melting temperatures of the thiothymine‐containing duplexes were very high (75.7 °C for **ON1z‐Hg_1_b⋅ON2s^2^t** and 82.0 °C for **ON1z‐Hg_1_b⋅ON2s^4^t**) and, gratifyingly, complementary to those of the corresponding duplexes formed by **ON1z‐Hg_1_a**. In all likelihood, the 6‐phenylcarbazole residue of **ON1z‐Hg_1_b** is mercurated at C8, conducive to Hg^II^‐mediated base pairing with 4‐thiothymine (Figure 2F).

Affinity of the dimercurated oligonucleotide **ON1z‐Hg_2_** for complementary sequences comprising only canonical nucleobases followed a pattern similar to those observed with **ON1z‐Hg_1_a** and **ON1z‐Hg_1_b** (T>G>C>A) but duplexes **ON1z‐Hg_2_⋅ON2a** and **ON1z‐Hg_2_⋅ON2t** melted at considerably lower temperatures than either of their monomercurated counterparts. The melting temperatures were also consistently lower than those reported previously^[32]^ for the same duplexes, probably owing to the relatively high affinity of the chloride ion (compared to the previously employed perchlorate ion) for Hg^II^. The two thiothymine‐containing duplexes were again the most stable by a wide margin but the difference between their melting temperatures (77.1 °C for **ON1z‐Hg_2_⋅ON2s^2^t** and 74.5 °C for **ON1z‐Hg_2_⋅ON2s^4^t**) was much smaller than with the corresponding monomercurated duplexes. This result was expected given the symmetric nature of the 1,8‐dimercuri‐6‐phenylcarbazole residue. Curiously, and in contrast to previous reports on related purely coordinative dinuclear Hg^II^‐mediated base pairs,[^38^, ^39^] the effect of the “extra” Hg^II^ ion, presumably coordinated to the 4‐ or 2‐oxo substituent (Figure 2C and D), was somewhat destabilizing.

Denaturation of the modified duplexes was also studied by CD spectropolarimetry for more information on their secondary structures. The samples were identical to those used in the UV melting temperature measurements and the spectra were acquired between 10 and 90 °C at 10 °C intervals. Representative examples of the CD spectra of duplexes formed by each of the modified oligonucleotides are presented in Figure 5 and spectra of all modified duplexes in the Supporting Information (Figures S38–S61).

**Figure 5:**
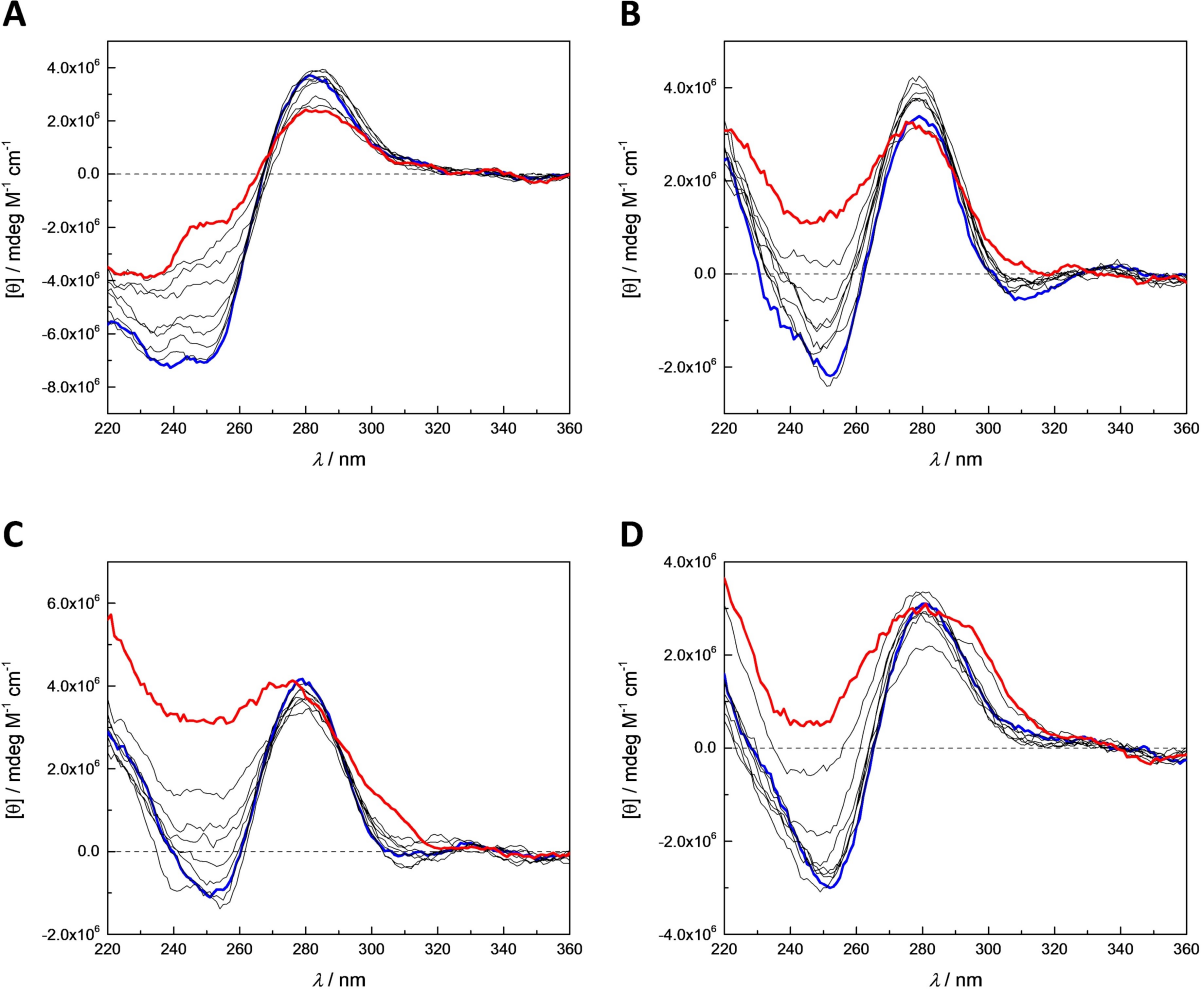
CD spectra of duplexes A) **ON1z⋅ON2s^2^t**, B) **ON1z‐Hg_1_a⋅ON2s^2^t**, C) **ON1z‐Hg_1_b⋅ON2s^2^t**, and D) **ON1z‐Hg_2_⋅ON2s^2^t**, recorded at 10 °C intervals between 10 and 90 °C (thick blue and red lines, respectively); pH 7.4 (20 mM cacodylate buffer); [oligonucleotides]=1.0 μM; *I*(NaCl)=0.10 M.

At 10 °C, CD spectra of the mercurated duplexes **ON1z‐Hg_1_a⋅ON2s^2^t** (Figure 5B), **ON1z‐Hg_1_b⋅ON2s^2^t** (Figure 5C) and **ON1z‐Hg_2_⋅ON2s^2^t** (Figure 5D) were characteristic of an ideal B‐type double helix, with sharp and symmetric positive and negative Cotton effects at 280 and 250 nm, respectively. With the unmercurated duplex **ON1z⋅ON2s^2^t** (Figure 5A), however, the negative Cotton effect was broader, extending further towards shorter wavelengths. The same general pattern was observed with all duplexes studied: while all CD spectra were consistent with a right‐handed double helix, only duplexes incorporating 2‐thiothymine opposite to one of the organometallic residues showed clearly B‐type spectra. Notably, the spectrum of duplex **ON1z‐Hg_1_b⋅ON2s^4^t** was somewhat distorted despite its very high UV melting temperature. In all cases, the negative Cotton effect diminished with increasing temperature, consistent with unwinding of the double helix. The positive Cotton effect also diminished in most cases but remained almost unchanged with duplexes incorporating a Hg^II^‐mediated base pair with either 2‐thiothymine or 4‐thiothymine, consistent with the high melting temperature of these duplexes.

### DFT calculations

All of the mercurated oligonucleotides hybridized much more strongly with both of the thiothymine‐containing complementary sequences than with any of the canonical sequences, strongly suggesting coordination of Hg^II^ to S2 or S4 as the driving force. Pairing of 1‐mercuri‐6‐phenylcarbazole with 2‐thiothymine (Figure 2E) or 8‐mercuri‐6‐phenylcarbazole with 4‐thiothymine (Figure 2F) would allow such coordination with little deviation from the geometry of a canonical Watson‐Crick base pair. The opposite pairings, on the other hand, would require either less favorable coordination of Hg^II^ to O4 or O2 or highly distorted geometry of the Hg^II^‐mediated base pair. A set of DFT calculations was carried out to help identify the most likely binding mode for each Hg^II^‐mediated base pair and to further elucidate the ability of the monomercurated carbazoles to discriminate between 2‐ and 4‐thiothymines. The calculations were performed at the PBE0DH level of theory^[40]^ employing def‐2SVP basis set and pseudopotential for Hg,^[41]^ 6‐31+G(d,p) basis set for N, O and S^[42]^ and 6‐31G(d,p) basis set for C and H atoms.^[43]^ The structures shown in Figure 2C−F were used as starting geometries but the sugar moieties were replaced by methyl groups and the phenyl substituent of carbazole by a hydrogen atom to simplify the system. At neutral pH, thymidine and uridine are known to deprotonate on coordination of Hg^II[44]^ and their 2‐ and 4‐thio analogues are even more acidic.[^45^, ^46^] Accordingly, both 2‐ and 4‐thiothymine are in all likelihood monoanionic in the Hg^II^‐mediated base pairs studied, resulting in an overall charge of +1 for the dinuclear and 0 for the mononuclear ones.

**Figure 6:**
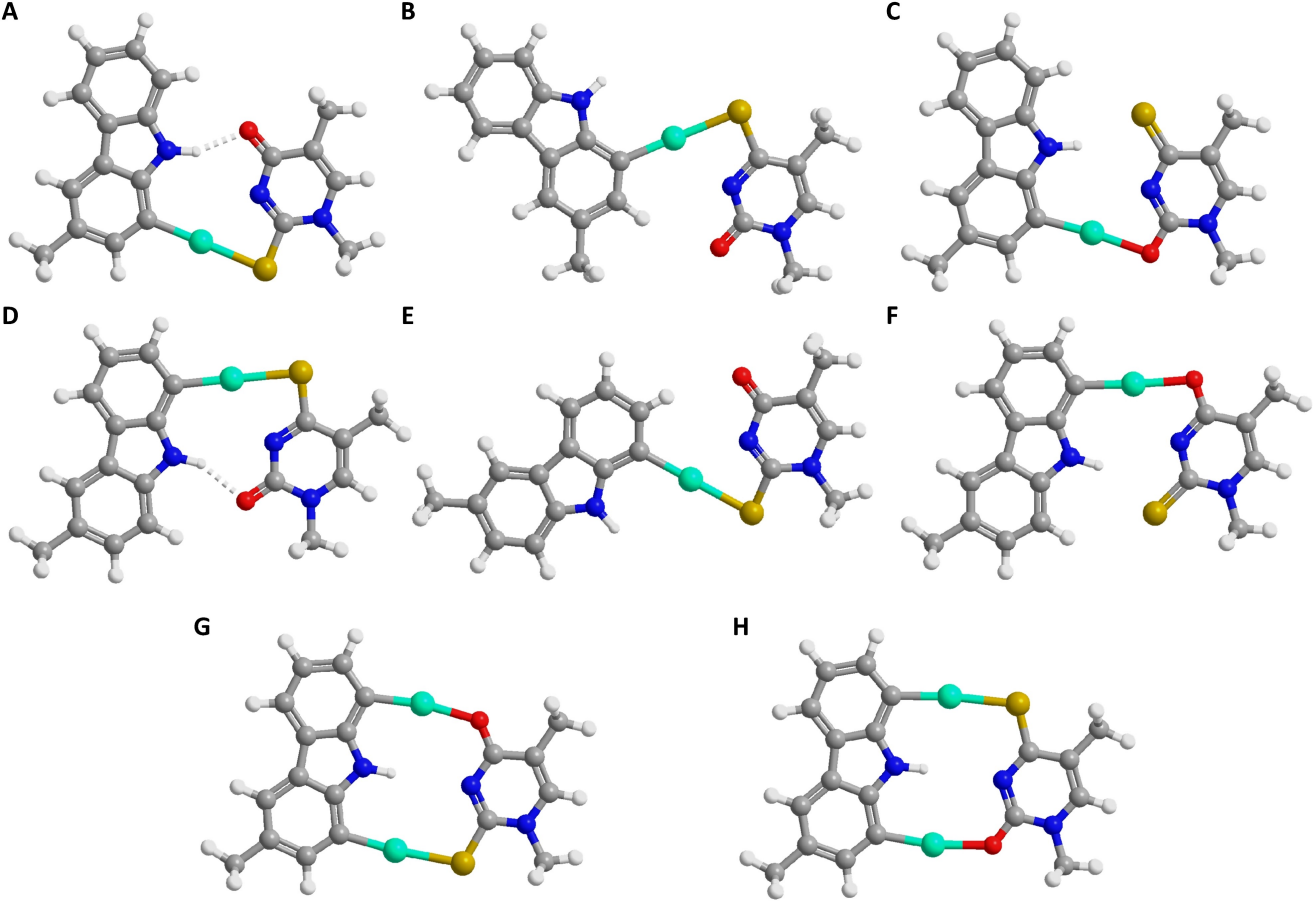
Optimized geometries for Hg^II^‐mediated base pairing between 1‐mercuri‐3‐methyl‐carbazole and A) 2‐thio‐ or B) and C) 4‐thio‐1‐methylthymine, 8‐mercuri‐3‐methyl‐carbazole and D) 4‐thio‐ or E) and F) 2‐thio‐1‐methylthymine, and 1,8‐dimercuri‐3‐methyl‐carbazole and G) 2‐thio‐ or H) 4‐thio‐1‐methylthymine.

The optimized geometry of the Hg^II^‐mediated base pair between 1‐mercuri‐carbazole and 2‐thiothymine (Figure 6A) was strictly planar with minimal shearing and thus appeared compatible with a B‐type double helix. Distance between the anomeric carbons was 10.6, the canonical value being 10.7 Å.^[47]^ In all likelihood the Hg^II^‐mediated base pair within duplex **ON1z‐Hg_1_a⋅ON2s^2^t** also adopts this geometry. Pairing with 4‐thiothymine, on the other hand, is less clear‐cut as formation of the strong Hg−S bond results in a highly distorted structure (Figure 6B, interanomeric distance 7.5 Å) while the more compatible structure (Figure 6C) would involve a weaker Hg−O bond. In the absence of constraints imposed by the double‐helical environment, the former base pair is 48 kJ mol^−1^ more stable than the latter. Furthermore, the 16 °C higher melting temperature of duplex **ON1z‐Hg_1_a⋅ON2s^4^t** compared to **ON1z‐Hg_1_a⋅ON2t** would be difficult to explain in terms of the latter structure. We therefore propose that Hg^II^ coordinates to sulfur also in duplex **ON1z‐Hg_1_a⋅ON2s^4^t** and that the difference in the melting temperatures of **ON1z‐Hg_1_a⋅ON2s^2^t** and **ON1z‐Hg_1_a⋅ON2s^4^t** stems from the distorted geometry of the Hg^II^‐mediated base pair and the absence of a stabilizing hydrogen bond in the latter. This interpretation receives support also from the CD experiments – at 10 °C, the spectrum of **ON1z‐Hg_1_a⋅ON2s^2^t** was essentially identical to that of an ideal B‐type double helix whereas in the case of **ON1z‐Hg_1_a⋅ON2s^4^t** the negative Cotton effect was less intensive but broader.

While also planar, the Hg^II^‐mediated base pair of 8‐mercuri‐carbazole with 4‐thiothymine (Figure 6D) was somewhat more distorted than that of 1‐mercuri‐carbazole with 2‐thiothymine, featuring considerable opening as well as shearing. Distance between the anomeric carbons was 9.4 Å. When pairing with 2‐thiothymine, S‐coordination (Figure 6E) was favored over O‐coordination (Figure 6F) by 14 kJ mol^−1^. For reasons discussed above, we propose that Hg^II^ coordinates to sulfur in both **ON1z‐Hg_1_b⋅ON2s^2^t** and **ON1z‐Hg_1_b⋅ON2s^4^t** and that distorted geometry of the Hg^II^‐mediated base pair (interanomeric distance 14.1 Å) and the absence of a stabilizing hydrogen bond accounts for the lower melting temperature of the former. Similarly, the slightly different thermal stabilities of duplexes **ON1z‐Hg_1_b⋅ON2s^4^t** and **ON1z‐Hg_1_a⋅ON2s^2^t** can be explained in terms of compatibility of the geometries of the Hg^II^‐mediated base pairs (Figure 6D and A, respectively) with that of a B‐type double helix.

The geometries of the dinuclear Hg^II^‐mediated base pairs between 1,8‐dimercuri‐carbazole and 2‐thiothymine (Figure 6G) or 4‐thiothymine (Figure 6H) closely resembled the one obtained previously for the analogous base pair with thymine.^[32]^ Both base pairs were strictly planar, with 10.8 and 10.1 Å distances between the anomeric carbon atoms, respectively. In other words, the nearly 30 °C higher melting temperature of duplexes **ON1z‐Hg_2_⋅ON2s^2^t** and **ON1z‐Hg_2_⋅ON2s^4^t** compared to **ON1z‐Hg_2_⋅ON2t** must stem from the strength of the single Hg−S bond, rather than differences in the shape of the three dinuclear Hg^II^‐mediated base pairs. Such differences could, however, account for the much smaller difference between the melting temperatures of **ON1z‐Hg_2_⋅ON2s^2^t** and **ON1z‐Hg_2_⋅ON2s^4^t**. The somewhat lower thermal stability of **ON1z‐Hg_2_⋅ON2s^2^t** and **ON1z‐Hg_2_⋅ON2s^4^t** compared to the “matched” duplexes formed by the monomercurated oligonucleotides (**ON1z‐Hg_1_a⋅ON2s^2^t** and **ON1z‐Hg_1_b⋅ON2s^4^t**) was unexpected but could be related to a higher entropic penalty of hybridization involving formation of a more rigid dinuclear Hg^II^‐mediated base pair or the loss of a stabilizing hydrogen bond between O2 or O4 and the carbazole nitrogen. Unfortunately, the very high melting temperatures of all of these duplexes precluded a more detailed thermodynamic analysis of the melting curves, as leveling‐off was not complete even at 90 °C.

## Conclusion

Oligonucleotides incorporating an organometallic derivative of 6‐phenylcarbazole as a nucleobase surrogate hybridize very strongly with complementary sequences placing a 2‐ or 4‐thiothymine residue opposite the modified site, undoubtedly owing to the very high affinity of mercury for sulfur. Furthermore, monomercurated derivatives facilitating Hg^II^ coordination to either S2 or S4 allowed reliable discrimination between 2‐ and 4‐thiothymine‐containing sequences based on the different melting temperatures of the duplexes formed. 1‐Mercuri‐6‐phenylcarbazole, in particular, appeared promising as a modification for oligonucleotide hybridization probes, differentiating between all canonical nucleobases as well as 2‐ and 4‐thiothymine by a margin of at least 5.5 °C. In addition to application as hybridization probes for detecting single‐nucleotide polymorphismis within the epitranscriptome, the very high affinity of the mono‐ and dimercurated 6‐phenylcarbazoles for thiopyrimidine bases should allow sequence‐specific pull‐down isolation of corresponding nucleic acids, notably tRNAs, from complex mixtures.

## Experimental Section

**General methods**: HPLC elution buffers (pH 7.0) were prepared by using freshly distilled analytical grade Et_3_N. All other reagents and solvents, as well as the unmodified oligonucleotides **ON1a**, **ON2a**, **ON2c**, **ON2g** and **ON2t**, were commercial products and were used as received. Mass spectra were recorded on a Bruker Daltonics micrOTOF−Q ESI‐TOF mass spectrometer.

**Oligonucleotide synthesis**: Oligonucleotides **ON2s^2^t**, **ON2s^4^t** and **ON1z** were assembled by an ÄKTA oligopilot plus 10 DNA/RNA synthesizer on a CPG support following conventional phosphoramidite strategy, with 5‐(benzylthio)‐1*H*‐tetrazole as the activator. Based on the trityl response, all couplings proceeded with normal, near‐quantitative, efficiency. After synthesis, **ON2s^2^t** and **ON1z** were incubated in 25 % aqueous NH_3_ at 55 °C for 16 h to cleave the linker as well as the phosphate and base protections. With **ON2s^4^t**, the cyanoethyl protecting groups of the phosphate linkages were first removed by treatment with 1.0 M DBU in anhydrous acetonitrile at 25 °C for 2 h, followed by complete deprotection with 50 mM NaSH in 25 % aqueous NH_3_ at 25 °C for 24 h. This treatment is recommended by the manufacturer to avoid ammonolysis of the *S*‐cyanoethyl moiety. The crude oligonucleotides were purified by RP‐HPLC on a Hypersil ODS C_18_ column (250×4.6 mm, 5 μm) eluting with a linear gradient of MeCN (5–45 % over 20 min, flow rate=1.0 mL min^−1^) in 50 mM triethylammonium acetate buffer (pH 7.0). The detection wavelength was 260 nm.

To prepare the mercurated oligonucleotides **ON1z‐Hg_1_a**, **ON1z‐Hg_1_b** and **ON1z‐Hg_2_**, **ON1z** (30 nmol) was incubated with Hg(OAc)_2_ (10 μmol) and NaOAc (20 μmol) in H_2_O (250 μL) at 55 °C for 12 h. After 12 h, saturated aqueous NaCl (100 μL) was added to precipitate the excess mercury. The crude product mixture was centrifuged and the supernatant fractioned by RP‐HPLC on a Hypersil ODS C18 column (250×4.6 mm, 5 μm) eluting with a linear gradient of MeCN (5–40 % over 25 min, flow rate=1.0 mL min^−1^) in 50 mM triethylammonium acetate buffer (pH 7.0) containing a 10 mM concentration of EtSH. Inclusion of EtSH in the elution buffers was crucial for good separation of **ON1z‐Hg_1_a**, **ON1z‐Hg_1_b** and **ON1z‐Hg_2_**, but over time caused demercuration at room temperature. Keeping the collected fractions at 0 °C and lyophilizing them promptly after purification largely eliminated this problem. The purified oligonucleotides were characterized mass spectrometrically and quantified UV spectrophotometrically based on molar absorptivities calculated by an implementation of the nearest‐neighbors method. A previously determined^[32]^ value of *ϵ*=124 000 L mol^−1^ cm^−1^ was used for the 6‐phenylcarbazole residue. Isolated yields of **ON1z‐Hg_1_a**, **ON1z‐Hg_1_b** and **ON1z‐Hg_2_** were 8.6, 4.8 and 6.9 %, respectively.

**UV melting temperature measurements**: UV melting temperature measurements were carried out on a PerkinElmer Lambda 35 UV/vis spectrophotometer equipped with a Peltier temperature control unit. Samples were prepared by mixing appropriate oligonucleotides (1.0 μM), cacodylate buffer (20 mM, pH 7.4) and NaCl (total *I*=0.10 M) to a final volume of 400 μL and placed in quartz cuvettes with 10 mm optical path length. NaCl was used instead of NaClO_4_ for adjusting the ionic strength to avoid oxidation of the thio‐modified oligonucleotides **ON2s^2^t** and **ON2s^4^t**. UV melting profiles were measured by recording the absorbance at 260 nm at 0.5 °C intervals over a temperature range of 10–90 °C . Three heating and cooling ramps (0.5 °C min^−1^) were run for each sample. The melting temperatures (*T*_m_) were determined as the maxima of the first derivates of the melting curves.

**CD spectropolarimetric measurements**: CD spectra were recorded on an Applied Photophysics Chirascan spectropolarimeter equipped with a Peltier temperature control unit. The sample preparation procedure and the cuvettes were the same as described above for the UV melting temperature measurements. Spectra were recorded between 200 to 400 nm over a temperature range of 10–90 °C at 10 °C intervals. Samples were allowed to equilibrate for 2 min at each temperature before measuring.

**DFT calculations**: Geometry optimizations were performed with Gaussian 16 software^[48]^ utilizing PBE0DH double hybrid method^[40]^ and def‐2VSP basis set and pseudopotential for Hg,^[41]^ 6‐31+G(d,p) basis set for N, O and S^[42]^ and 6‐31G(d,p) basis set for C and H atoms.^[43]^ For comparison, geometry optimizations were performed also with a computationally lighter hybrid functional PBE0^[49]^ utilizing the same basis sets as with the double hybrid method. The results were found to be essentially the same. Coordinates are presented in Tables S2–S7 and a comparison of the calculated energies in Table S8.

## Conflict of interest

The authors declare no conflict of interest.

1

## Supporting information

As a service to our authors and readers, this journal provides supporting information supplied by the authors. Such materials are peer reviewed and may be re‐organized for online delivery, but are not copy‐edited or typeset. Technical support issues arising from supporting information (other than missing files) should be addressed to the authors.

Supporting Information

## Data Availability

The data that support the findings of this study are available in the supplementary material of this article.
