# Supplementary material for: Oligonucleotides Featuring a Covalently Mercurated 6‐Phenylcarbazole Residue as High‐Affinity Hybridization Probes for Thiopyrimidine‐Containing Sequences
Source: Chemistry. 2022 Oct 19;28(69):e202202530. doi: 10.1002/chem.202202530 (PMC10092508; doi:10.1002/chem.202202530)
Supplement: Supplementary file 1 — Supporting Information [file CHEM-28-0-s001.pdf]

Chemistry–A European Journal

Supporting Information

Oligonucleotides Featuring a Covalently Mercurated 6-Phenylcarbazole Residue as High-Affinity Hybridization Probes for Thiopyrimidine-Containing Sequences

Tharun K. Kotammagari, Petri Tähtinen, and Tuomas Lönnberg*

Contents

Figure S1. HPLC traces of reaction and product mixtures of mercuration of oligonucleotide ON1z and purified oligonucleotides ON1z-Hg_1a, ON1z-Hg_1b and ON1z-Hg_2.	S3
Figure S2. Measured and simulated mass spectra of oligonucleotide ON1z.	S4
Figure S3. Measured and simulated mass spectra of oligonucleotide ON1z-Hg_1a.	S5
Figure S4. Measured and simulated mass spectra of oligonucleotide ON1z-Hg_1b.	S6
Figure S5. Measured and simulated mass spectra of oligonucleotide ON1z-Hg_2.	S7
Figure S6. UV melting curve and its first derivative of duplex ON1a•ON2a.	S8
Figure S7. UV melting curve and its first derivative of duplex ON1a•ON2c.	S8
Figure S8. UV melting curve and its first derivative of duplex ON1a•ON2g.	S9
Figure S9. UV melting curve and its first derivative of duplex ON1a•ON2t.	S9
Figure S10. UV melting curve and its first derivative of duplex ON1a•ON2s^2t.	S10
Figure S11. UV melting curve and its first derivative of duplex ON1a•ON2s^4t.	S10
Figure S12. UV melting curve and its first derivative of duplex ON1z•ON2a.	S11
Figure S13. UV melting curve and its first derivative of duplex ON1z•ON2c.	S11
Figure S14. UV melting curve and its first derivative of duplex ON1z•ON2g.	S12
Figure S15. UV melting curve and its first derivative of duplex ON1z•ON2t.	S12
Figure S16. UV melting curve and its first derivative of duplex ON1z•ON2s^2t.	S13
Figure S17. UV melting curve and its first derivative of duplex ON1z•ON2s^4t.	S13
Figure S18. UV melting curve and its first derivative of duplex ON1z-Hg_1a•ON2a.	S14
Figure S19. UV melting curve and its first derivative of duplex ON1z-Hg_1a•ON2c.	S14
Figure S20. UV melting curve and its first derivative of duplex ON1z-Hg_1a•ON2g.	S15
Figure S21. UV melting curve and its first derivative of duplex ON1z-Hg_1a•ON2t.	S15
Figure S22. UV melting curve and its first derivative of duplex ON1z-Hg_1a•ON2s^2t.	S16
Figure S23. UV melting curve and its first derivative of duplex ON1z-Hg_1a•ON2s^4t.	S16
Figure S24. UV melting curve and its first derivative of duplex ON1z-Hg_1b•ON2a.	S17
Figure S25. UV melting curve and its first derivative of duplex ON1z-Hg_1b•ON2c.	S17
Figure S26. UV melting curve and its first derivative of duplex ON1z-Hg_1b•ON2g.	S18
Figure S27. UV melting curve and its first derivative of duplex ON1z-Hg_1b•ON2t.	S18
Figure S28. UV melting curve and its first derivative of duplex ON1z-Hg_1b•ON2s^2t.	S19
Figure S29. UV melting curve and its first derivative of duplex ON1z-Hg_1b•ON2s^4t.	S19
Figure S30. UV melting curve and its first derivative of duplex ON1z-Hg_2•ON2a.	S20
Figure S31. UV melting curve and its first derivative of duplex ON1z-Hg_2•ON2c.	S20
Figure S32. UV melting curve and its first derivative of duplex ON1z-Hg_2•ON2g.	S21
Figure S33. UV melting curve and its first derivative of duplex ON1z-Hg_2•ON2t.	S21
Figure S34. UV melting curve and its first derivative of duplex ON1z-Hg_2•ON2s^2t.	S22
Figure S35. UV melting curve and its first derivative of duplex ON1z-Hg_2•ON2s^4t.	S22
Figure S36. Temperature-dependent absorbance of oligonucleotide ON1z-Hg_1a.	S23
Figure S37. Temperature-dependent absorbance of oligonucleotide ON1z-Hg_2.	S23
Table S1. Melting temperatures of the duplexes studied.	S24
Figure S38. CD spectra of duplex ON1z•ON2a.	S25
Figure S39. CD spectra of duplex ON1z•ON2c.	S25
Figure S40. CD spectra of duplex ON1z•ON2g.	S26

Contents (continued)

Figure S41. CD spectra of duplex ON1z•ON2t.	S26
Figure S42. CD spectra of duplex ON1z•ON2s^{2}t.	S27
Figure S43. CD spectra of duplex ON1z•ON2s^{4}t.	S27
Figure S44. CD spectra of duplex ON1z-Hg$_1$a•ON2a.	S28
Figure S45. CD spectra of duplex ON1z-Hg$_1$a•ON2c.	S28
Figure S46. CD spectra of duplex ON1z-Hg$_1$a•ON2g.	S29
Figure S47. CD spectra of duplex ON1z-Hg$_1$a•ON2t.	S29
Figure S48. CD spectra of duplex ON1z-Hg$_1$a•ON2s^{2}t.	S30
Figure S49. CD spectra of duplex ON1z-Hg$_1$a•ON2s^{4}t.	S30
Figure S50. CD spectra of duplex ON1z-Hg$_b$a•ON2a.	S31
Figure S51. CD spectra of duplex ON1z-Hg$_b$a•ON2c.	S31
Figure S52. CD spectra of duplex ON1z-Hg$_b$a•ON2g.	S32
Figure S53. CD spectra of duplex ON1z-Hg$_b$a•ON2t.	S32
Figure S54. CD spectra of duplex ON1z-Hg$_b$a•ON2s^{2}t.	S33
Figure S55. CD spectra of duplex ON1z-Hg$_b$a•ON2s^{4}t.	S33
Figure S56. CD spectra of duplex ON1z-Hg$_2$•ON2a.	S34
Figure S57. CD spectra of duplex ON1z-Hg$_2$•ON2c.	S34
Figure S58. CD spectra of duplex ON1z-Hg$_2$•ON2g.	S35
Figure S59. CD spectra of duplex ON1z-Hg$_2$•ON2t.	S35
Figure S60. CD spectra of duplex ON1z-Hg$_2$•ON2s^{2}t.	S36
Figure S61. CD spectra of duplex ON1z-Hg$_2$•ON2s^{4}t.	S36
Table S2. Cartesian coordinates of the optimized (PBE0DH) Hg(II)-mediated base pairs between 1-mercuri-3-methylcarbazole and either 1-methyl-2-thiothymine or 1-methyl-4-thiothymine.	S37
Table S3. Cartesian coordinates of the optimized (PBE0DH) Hg(II)-mediated base pairs between 8-mercuri-3-methylcarbazole and either 1-methyl-2-thiothymine or 1-methyl-4-thiothymine.	S38
Table S4. Cartesian coordinates of the optimized (PBE0DH) Hg(II)-mediated base pairs between 1,8-dimercuri-3-methylcarbazole and either 1-methyl-2-thiothymine or 1-methyl-4-thiothymine.	S39
Table S5. Cartesian coordinates of the optimized (PBE0) Hg(II)-mediated base pairs between 1-mercuri-3-methylcarbazole and either 1-methyl-2-thiothymine or 1-methyl-4-thiothymine.	S40
Table S6. Cartesian coordinates of the optimized (PBE0) Hg(II)-mediated base pairs between 8-mercuri-3-methylcarbazole and either 1-methyl-2-thiothymine or 1-methyl-4-thiothymine.	S41
Table S7. Cartesian coordinates of the optimized (PBE0) Hg(II)-mediated base pairs between 1,8-dimercuri-3-methylcarbazole and either 1-methyl-2-thiothymine or 1-methyl-4-thiothymine.	S42
Table S8. Calculated energies of the Hg(II)-mediated base pairs.	S43

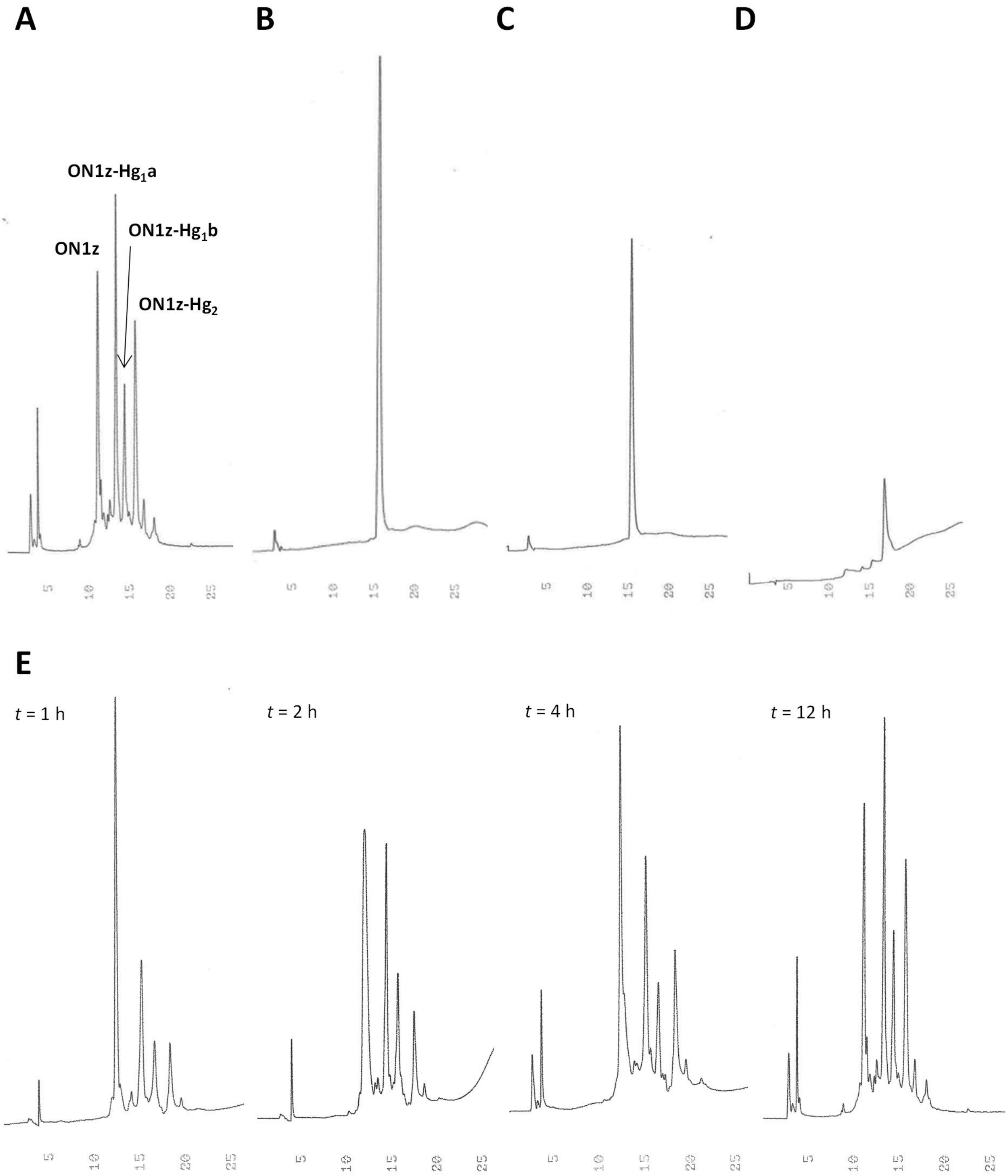

Figure S1. HPLC traces of A) product mixture of mercuration of oligonucleotide ON1z, B) purified oligonucleotide ON1z-Hg$_1$a, C) purified oligonucleotide ON1z-Hg$_1$b, D) purified oligonucleotide ON1z-Hg$_2$ and E) the mercuration reaction mixture at various time points; Hypersil ODS C18 column (250 x 4.6 mm, 5 µm); linear gradient of MeCN (5—40% over 25 min, flow rate = 1.0 mL min^{-1}) in 50 mM triethylammonium acetate buffer (pH = 7.0) containing a 10 mM concentration of EtSH; detection wavelength = 260 nm.

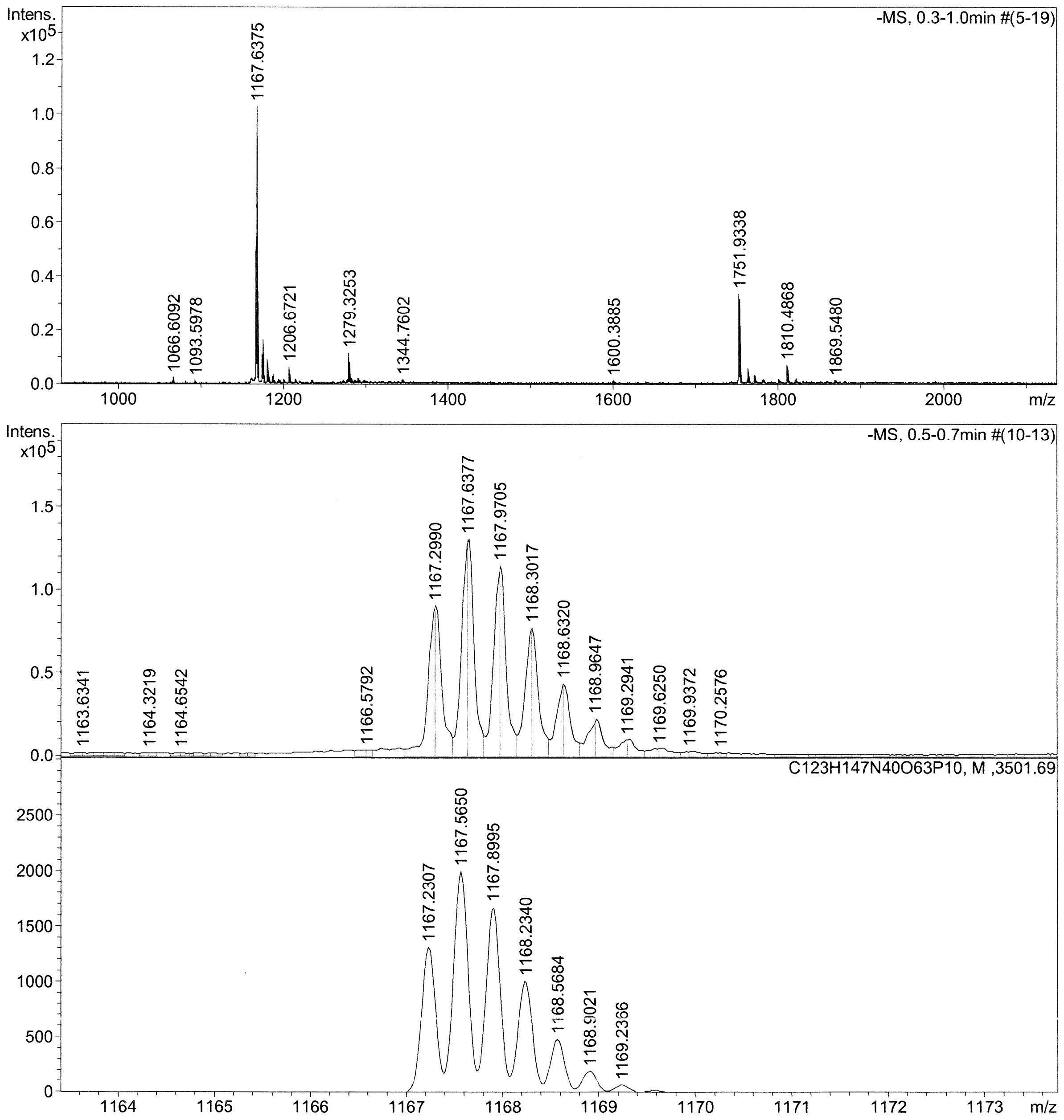

Figure S2. Measured (above) and simulated (below) mass spectra of oligonucleotide ON1z; *m*/*z*: calcd for $C_{123}H_{147}N_{40}O_{63}P_{10}$ [M – 3H]$^{3-}$: 1167.5650 and for $C_{123}H_{147}N_{40}O_{63}P_{10}$ [M – 2H]$^{2-}$: 1751.8514; found: 1167.6377 and 1751.9338.

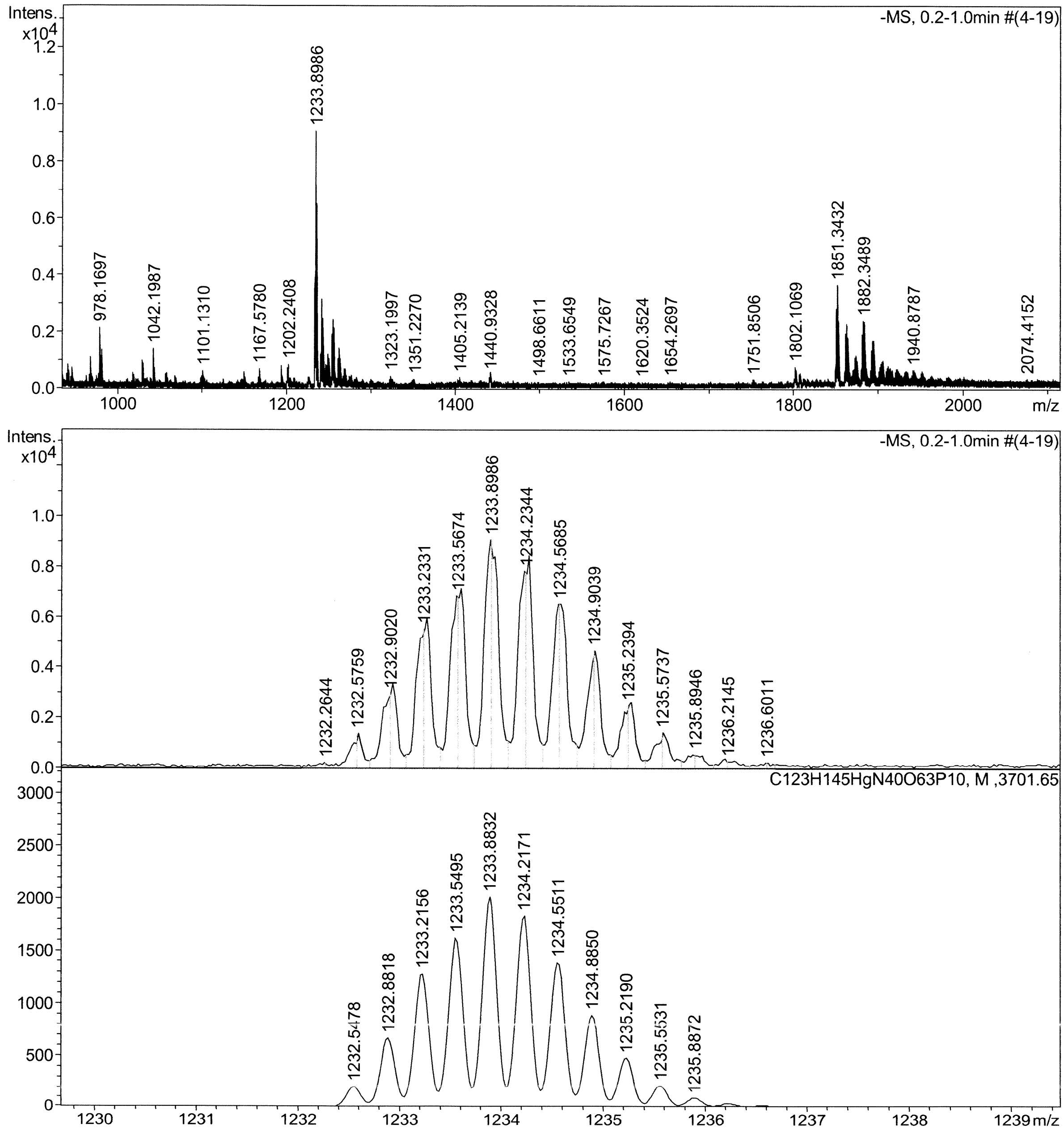

Figure S3. Measured (above) and simulated (below) mass spectra of oligonucleotide ON1z-Hg$_1$a; *m*/*z*: calcd for $C_{123}H_{145}HgN_{40}O_{63}P_{10}$ [M – 4H]$^{3-}$: 1233.8832 and for $C_{123}H_{146}HgN_{40}O_{63}P_{10}$ [M – 3H]$^{2-}$: 1851.8288; found: 1233.8986 and 1851.3432.

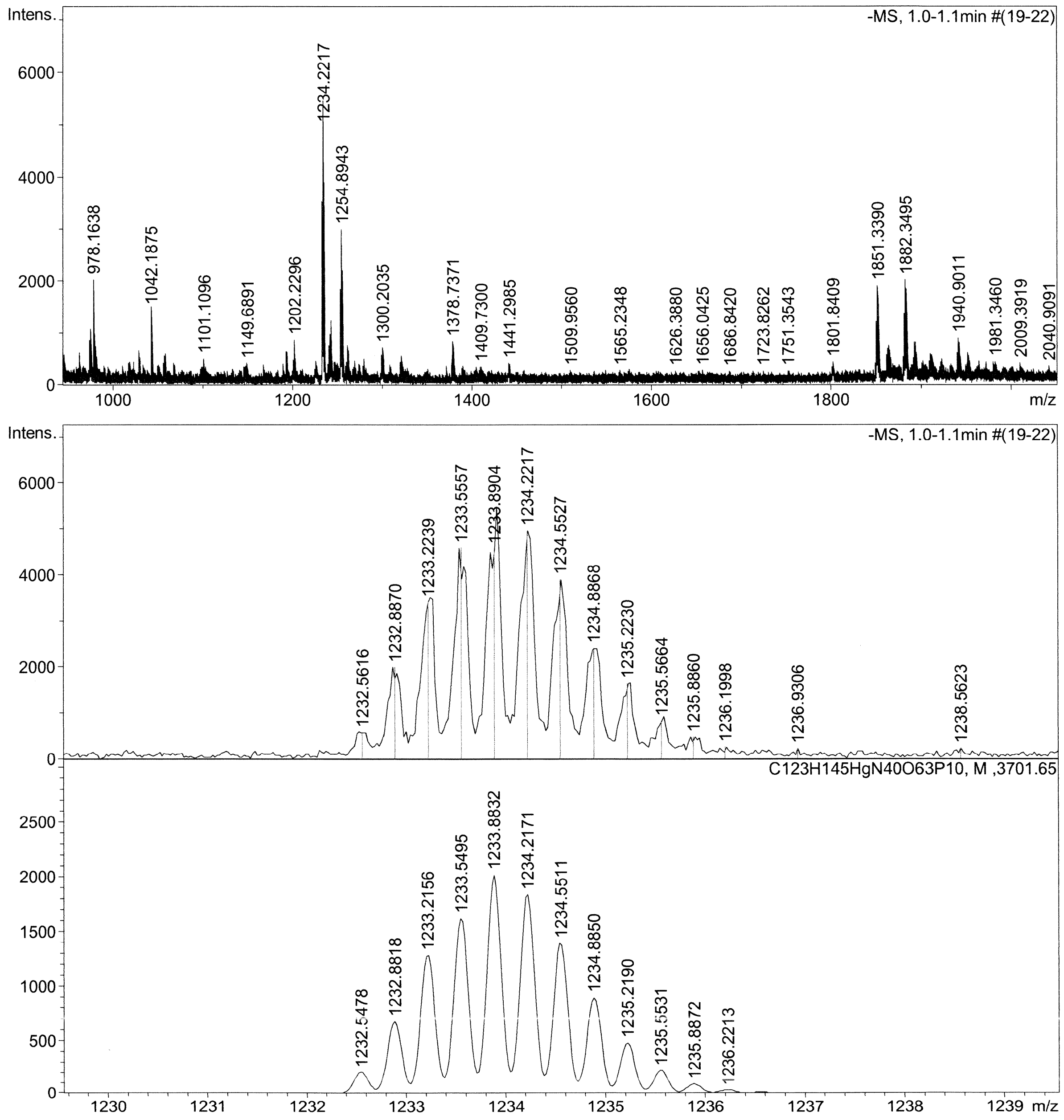

Figure S4. Measured (above) and simulated (below) mass spectra of oligonucleotide ON1z-Hg$_1$b; *m/z*: calcd for $C_{123}H_{145}HgN_{40}O_{63}P_{10}$ [M – 4H]$^{3-}$: 1233.8832 and for $C_{123}H_{146}HgN_{40}O_{63}P_{10}$ [M – 3H]$^{2-}$: 1851.8288; found: 1233.8904 and 1851.3390. The peaks at 60 Da higher molecular weight correspond to a complex with residual EtSH.

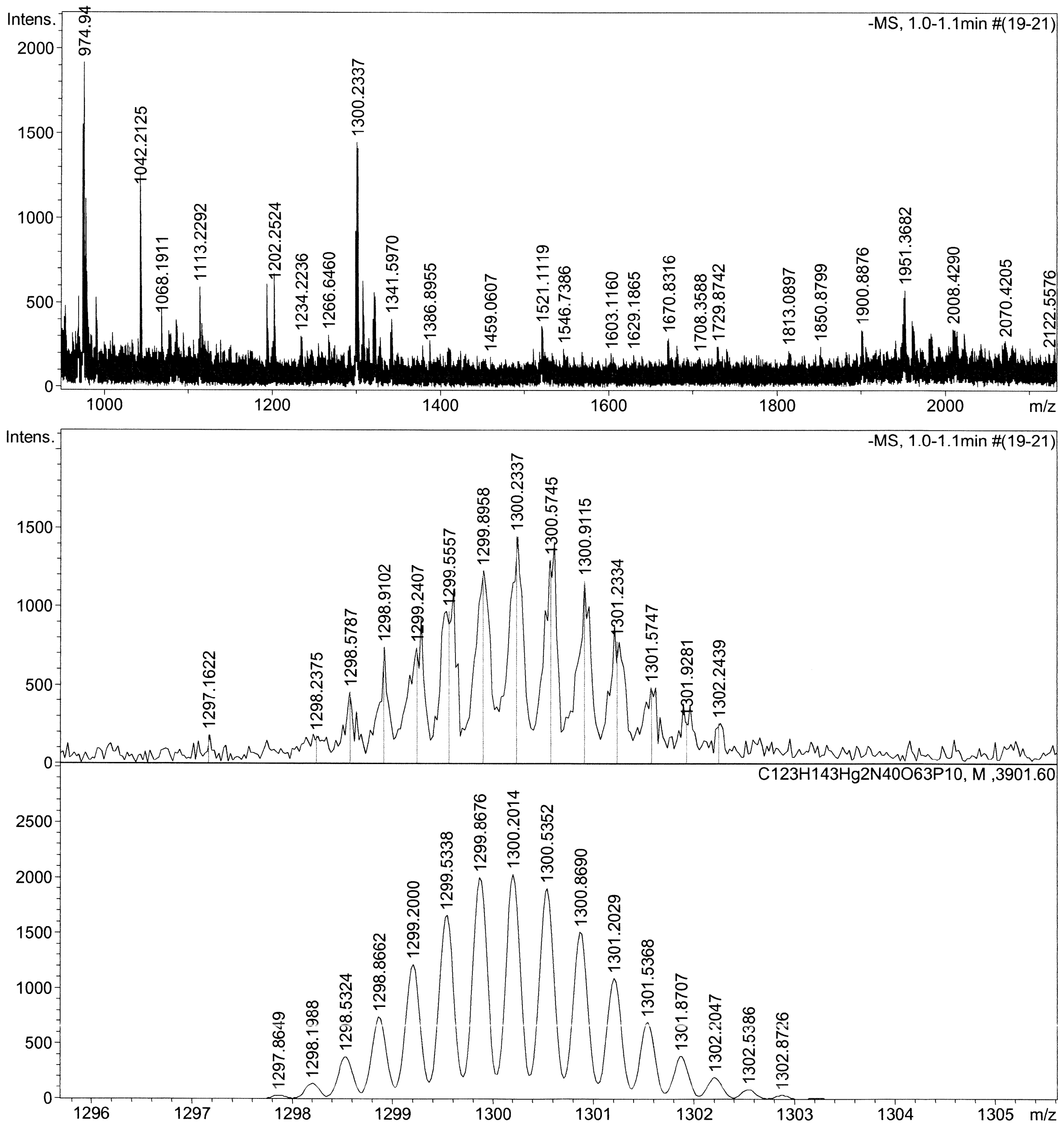

Figure S5. Measured (above) and simulated (below) mass spectra of oligonucleotide ON1z-Hg_2; *m/z*: calcd for $C_{123}H_{143}Hg_2N_{40}O_{63}P_{10}$ [M – 5H]$^{3-}$: 1300.2014 and for $C_{123}H_{143}Hg_2N_{40}O_{63}P_{10}$ [M – 4H]$^{2-}$: 1950.8052; found: 1300.2337 and 1951.3682. The peaks at 60 and 120 Da higher molecular weight correspond to complexes with one or two molecules of residual EtSH, respectively.

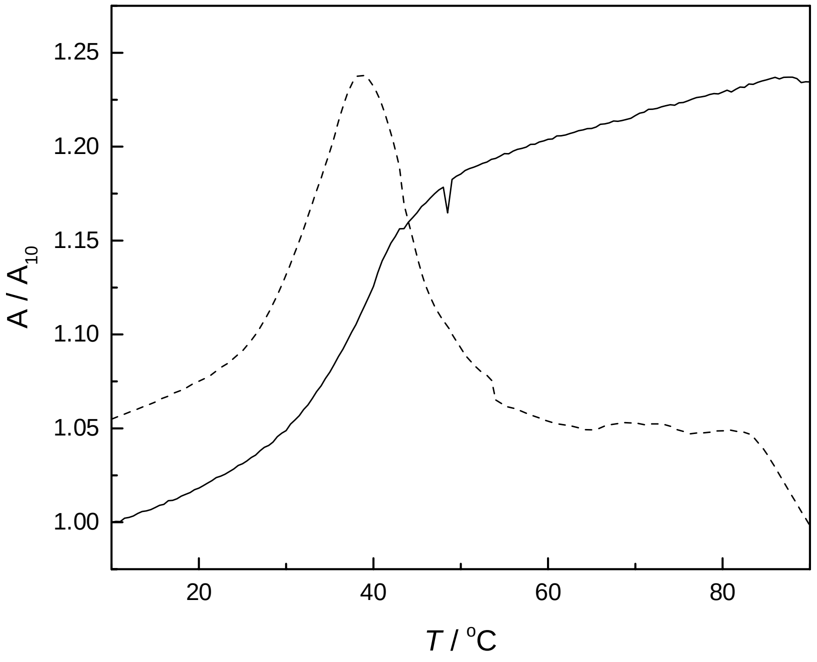

Figure S6. UV melting curve (solid line) and its first derivative (dashed line) of duplex ON1a•ON2a; pH = 7.4 (20 mM cacodylate buffer); [oligonucleotides] = 1.0 µM; *I*(NaCl) = 0.10 M.

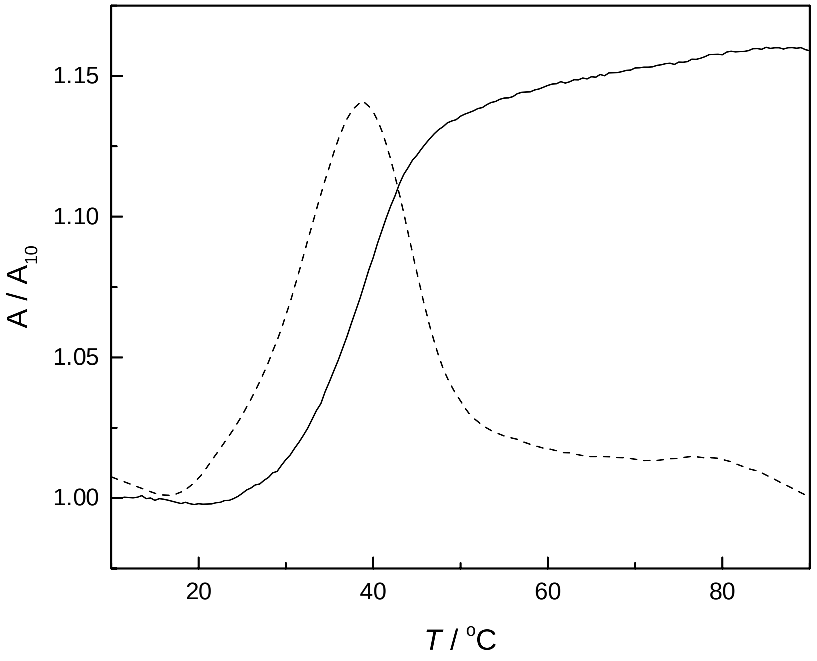

Figure S7. UV melting curve (solid line) and its first derivative (dashed line) of duplex ON1a•ON2c; pH = 7.4 (20 mM cacodylate buffer); [oligonucleotides] = 1.0 µM; *I*(NaCl) = 0.10 M.

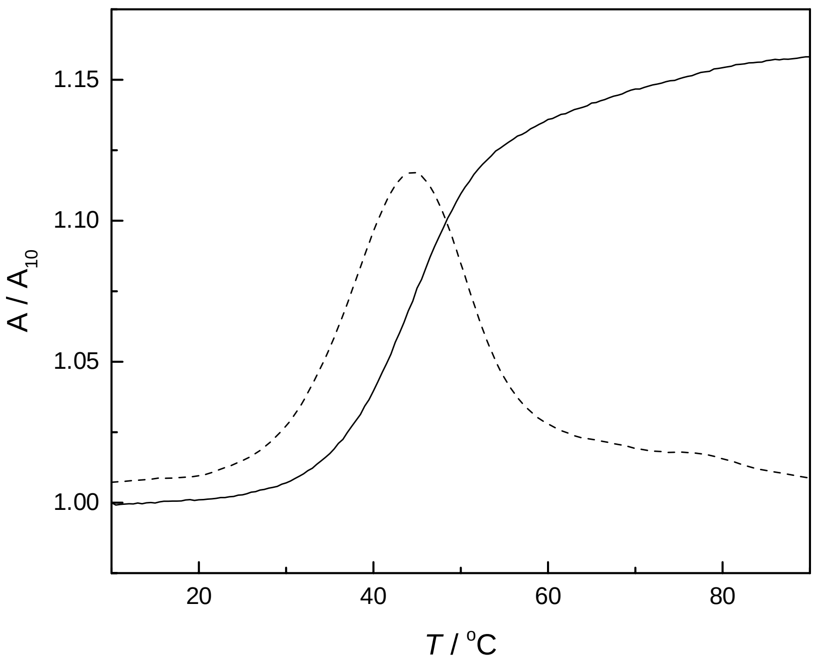

Figure S8. UV melting curve (solid line) and its first derivative (dashed line) of duplex ON1a•ON2g; pH = 7.4 (20 mM cacodylate buffer); [oligonucleotides] = 1.0 µM; *I*(NaCl) = 0.10 M.

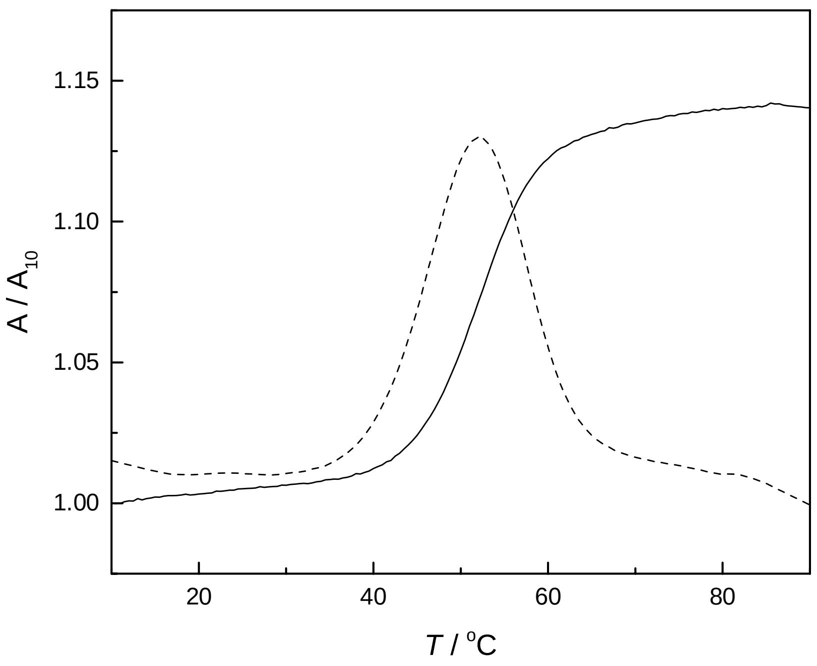

Figure S9. UV melting curve (solid line) and its first derivative (dashed line) of duplex ON1a•ON2t; pH = 7.4 (20 mM cacodylate buffer); [oligonucleotides] = 1.0 µM; *I*(NaCl) = 0.10 M.

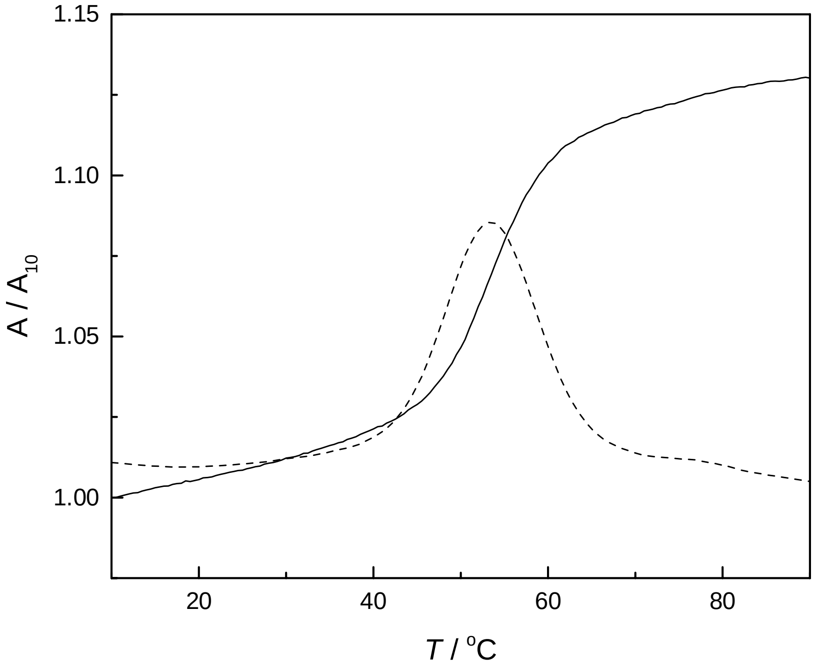

Figure S10. UV melting curve (solid line) and its first derivative (dashed line) of duplex ON1a•ON2s[2]t; pH = 7.4 (20 mM cacodylate buffer); [oligonucleotides] = 1.0 μM; *I*(NaCl) = 0.10 M.

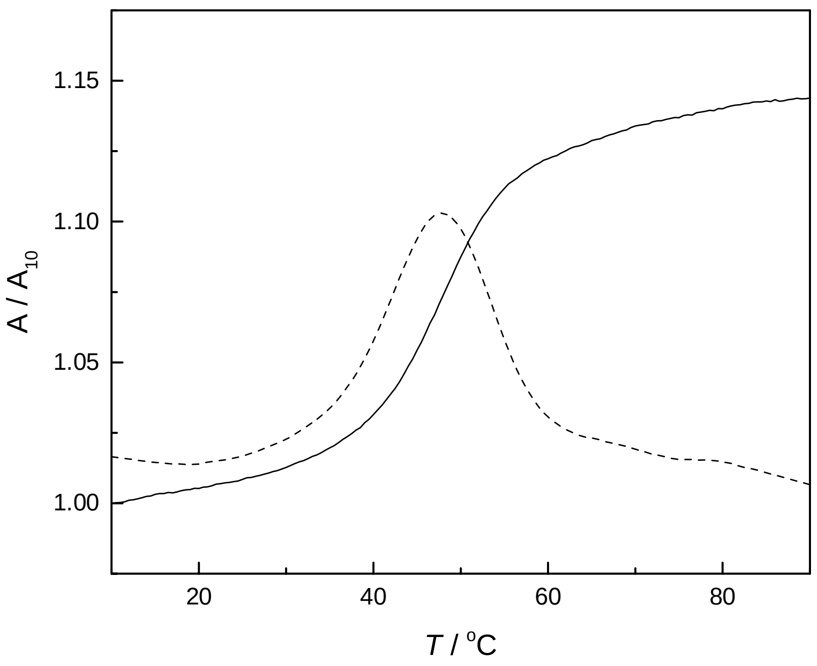

Figure S11. UV melting curve (solid line) and its first derivative (dashed line) of duplex ON1a•ON2s[4]t; pH = 7.4 (20 mM cacodylate buffer); [oligonucleotides] = 1.0 μM; *I*(NaCl) = 0.10 M.

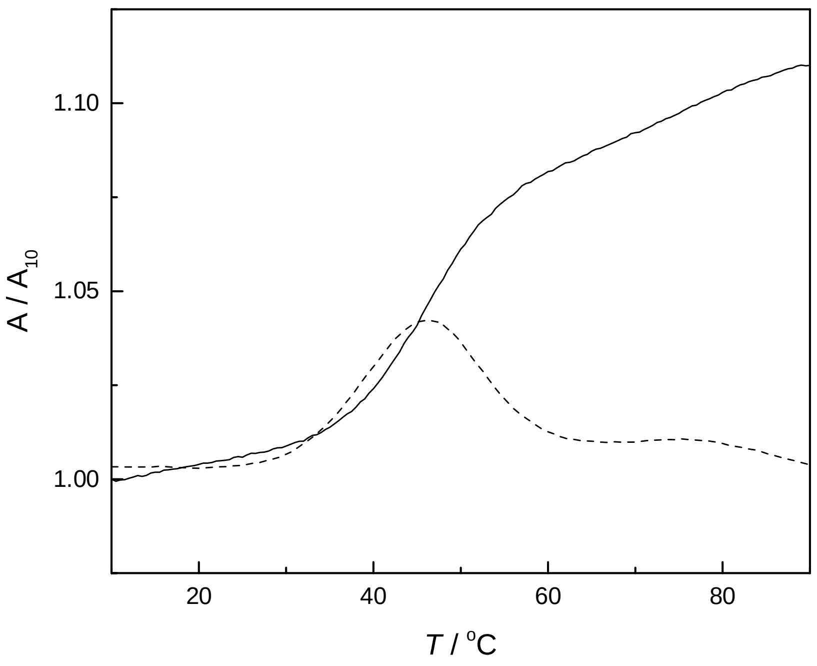

Figure S12. UV melting curve (solid line) and its first derivative (dashed line) of duplex ON1z•ON2a; pH = 7.4 (20 mM cacodylate buffer); [oligonucleotides] = 1.0 µM; *I*(NaCl) = 0.10 M.

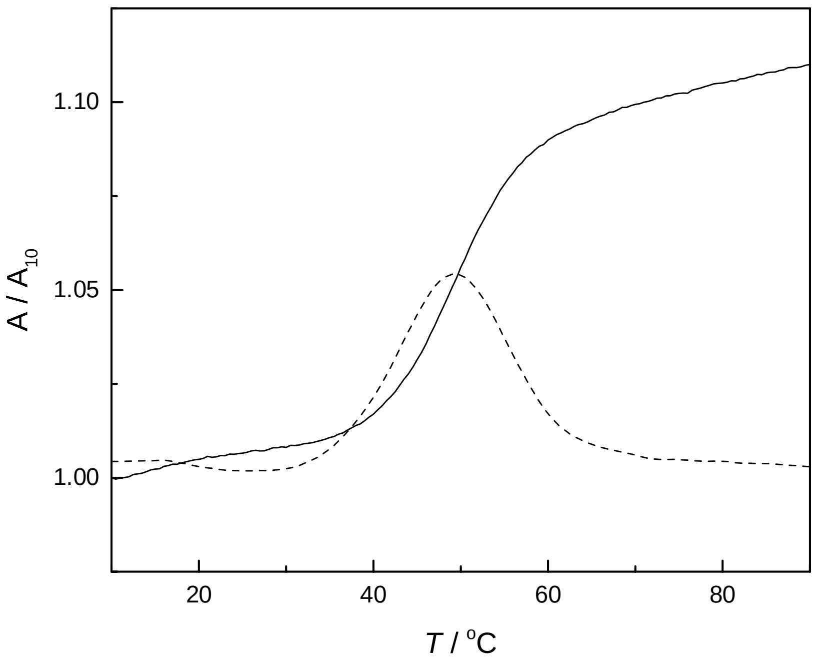

Figure S13. UV melting curve (solid line) and its first derivative (dashed line) of duplex ON1z•ON2c; pH = 7.4 (20 mM cacodylate buffer); [oligonucleotides] = 1.0 µM; *I*(NaCl) = 0.10 M.

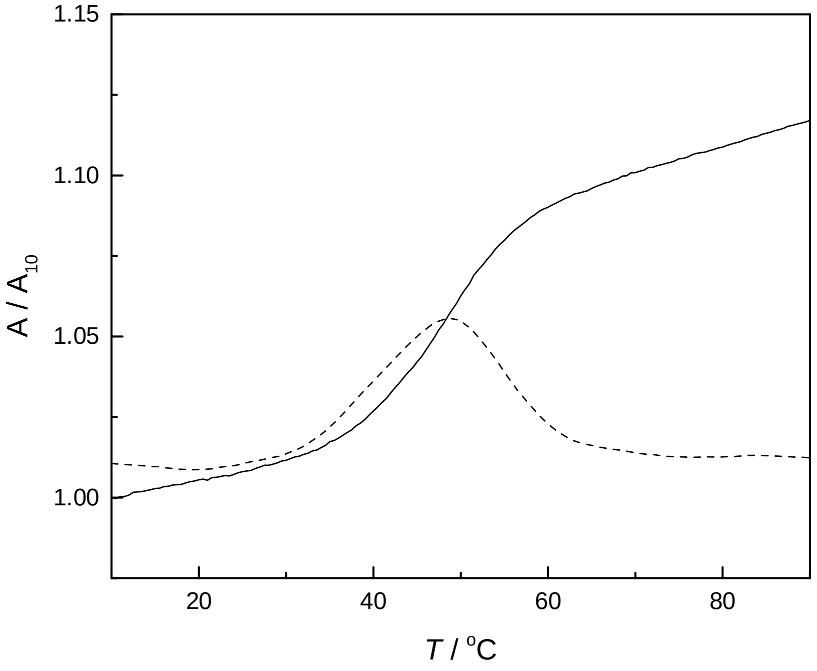

Figure S14. UV melting curve (solid line) and its first derivative (dashed line) of duplex ON1z•ON2g; pH = 7.4 (20 mM cacodylate buffer); [oligonucleotides] = 1.0 µM; *I*(NaCl) = 0.10 M.

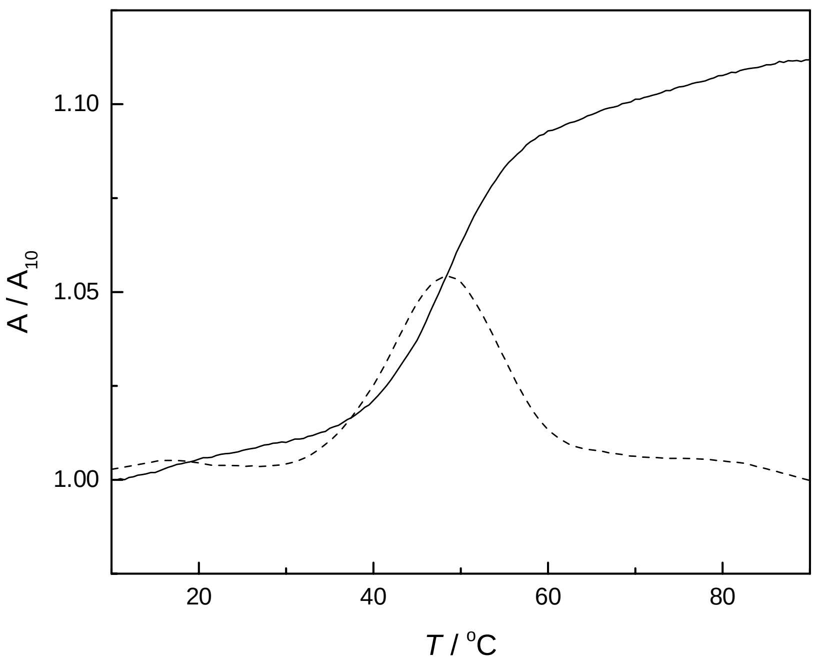

Figure S15. UV melting curve (solid line) and its first derivative (dashed line) of duplex ON1z•ON2t; pH = 7.4 (20 mM cacodylate buffer); [oligonucleotides] = 1.0 µM; *I*(NaCl) = 0.10 M.

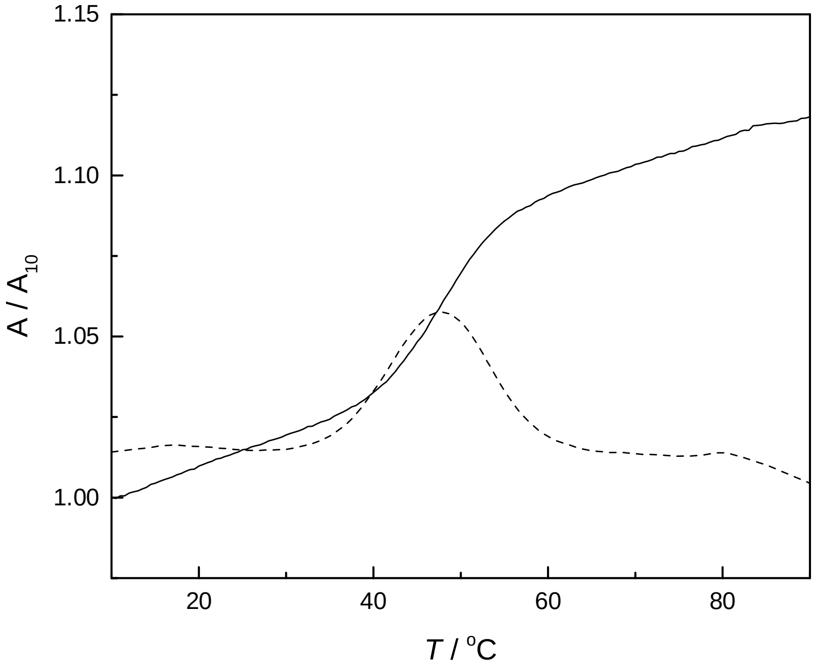

Figure S16. UV melting curve (solid line) and its first derivative (dashed line) of duplex ON1z•ON2s[2]t; pH = 7.4 (20 mM cacodylate buffer); [oligonucleotides] = 1.0 µM; *I*(NaCl) = 0.10 M.

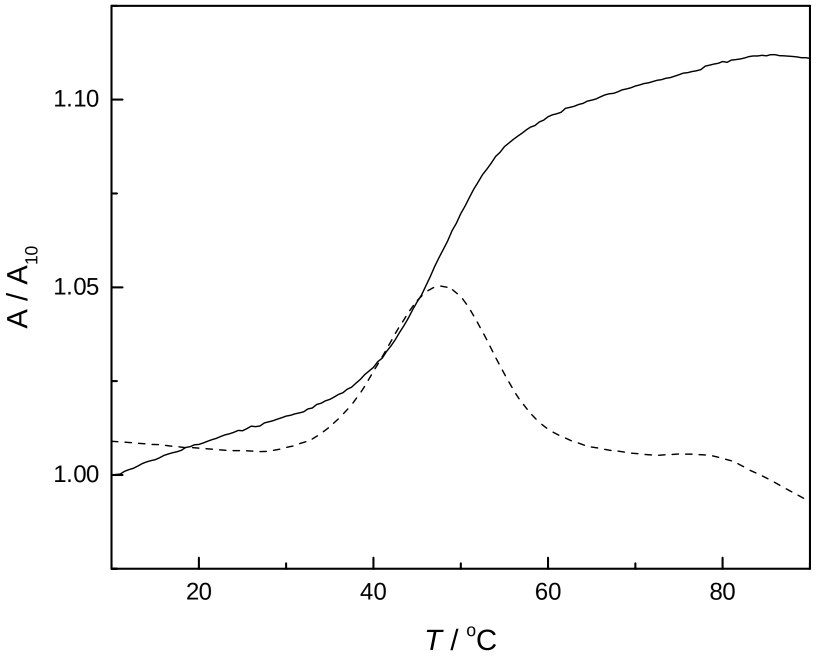

Figure S17. UV melting curve (solid line) and its first derivative (dashed line) of duplex ON1z•ON2s[4]t; pH = 7.4 (20 mM cacodylate buffer); [oligonucleotides] = 1.0 µM; *I*(NaCl) = 0.10 M.

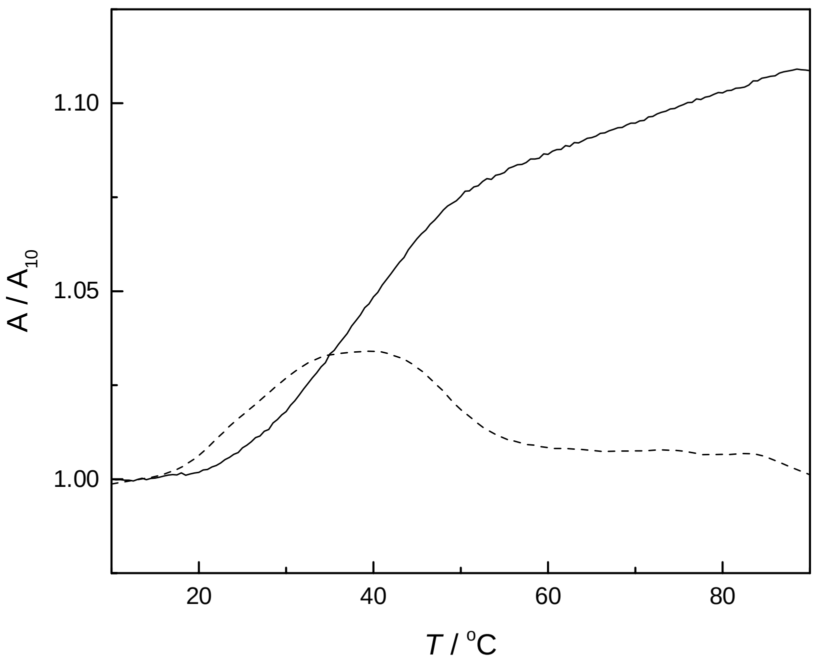

Figure S18. UV melting curve (solid line) and its first derivative (dashed line) of duplex ON1z-Hg_1a•ON2a; pH = 7.4 (20 mM cacodylate buffer); [oligonucleotides] = 1.0 µM; I(NaCl) = 0.10 M.

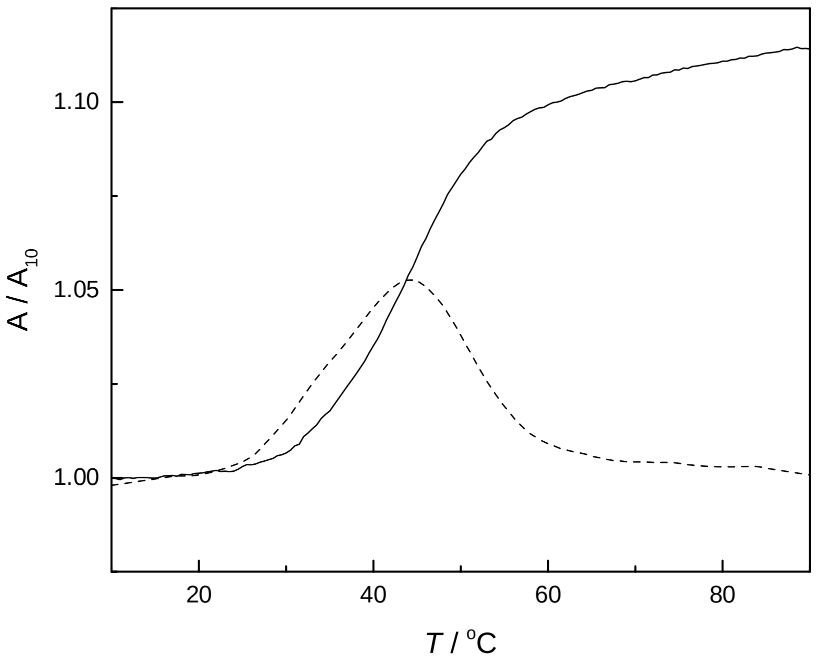

Figure S19. UV melting curve (solid line) and its first derivative (dashed line) of duplex ON1z-Hg_1a•ON2c; pH = 7.4 (20 mM cacodylate buffer); [oligonucleotides] = 1.0 µM; I(NaCl) = 0.10 M.

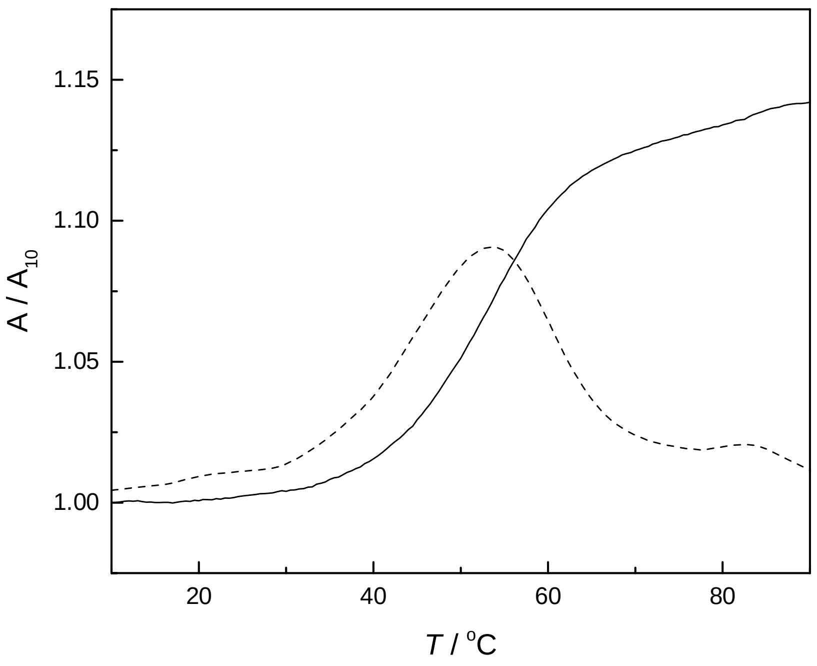

Figure S20. UV melting curve (solid line) and its first derivative (dashed line) of duplex ON1z-Hg$_1$a•ON2g; pH = 7.4 (20 mM cacodylate buffer); [oligonucleotides] = 1.0 µM; *I*(NaCl) = 0.10 M.

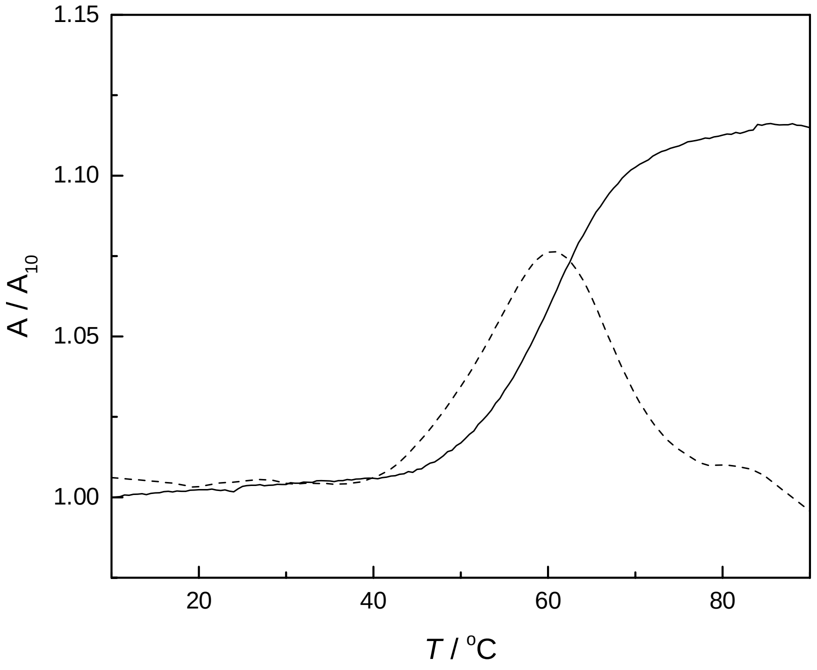

Figure S21. UV melting curve (solid line) and its first derivative (dashed line) of duplex ON1z-Hg$_1$a•ON2t; pH = 7.4 (20 mM cacodylate buffer); [oligonucleotides] = 1.0 µM; *I*(NaCl) = 0.10 M.

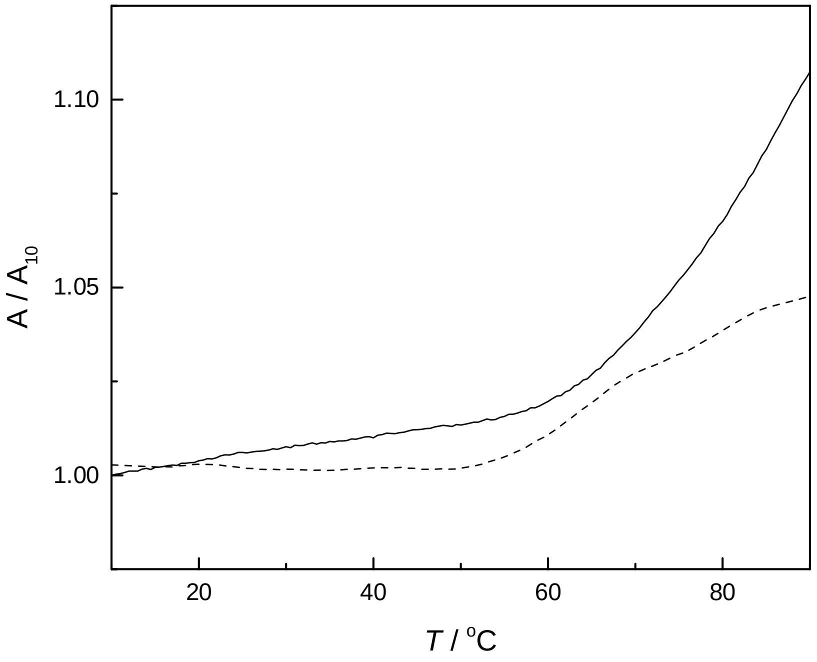

Figure S22. UV melting curve (solid line) and its first derivative (dashed line) of duplex ON1z-Hg_1a•ON2s^{2}t; pH = 7.4 (20 mM cacodylate buffer); [oligonucleotides] = 1.0 µM; *I*(NaCl) = 0.10 M.

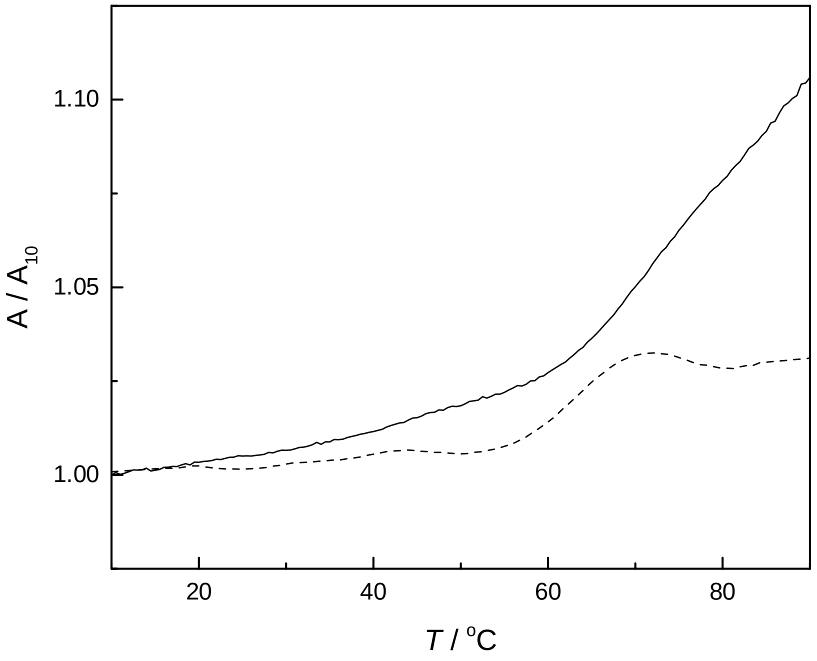

Figure S23. UV melting curve (solid line) and its first derivative (dashed line) of duplex ON1z-Hg_1a•ON2s^{4}t; pH = 7.4 (20 mM cacodylate buffer); [oligonucleotides] = 1.0 µM; *I*(NaCl) = 0.10 M.

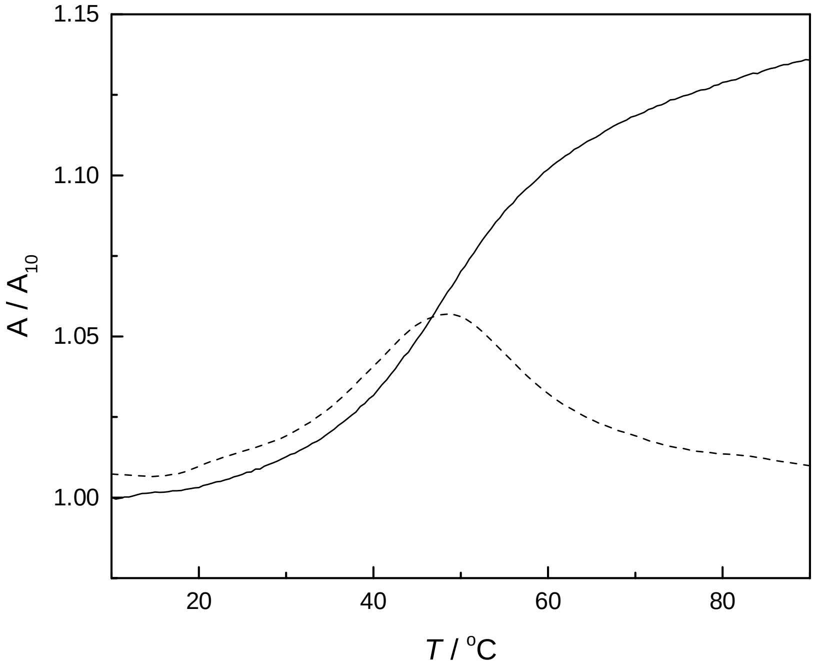

Figure S24. UV melting curve (solid line) and its first derivative (dashed line) of duplex ON1z-Hg$_1$b•ON2a; pH = 7.4 (20 mM cacodylate buffer); [oligonucleotides] = 1.0 µM; *I*(NaCl) = 0.10 M.

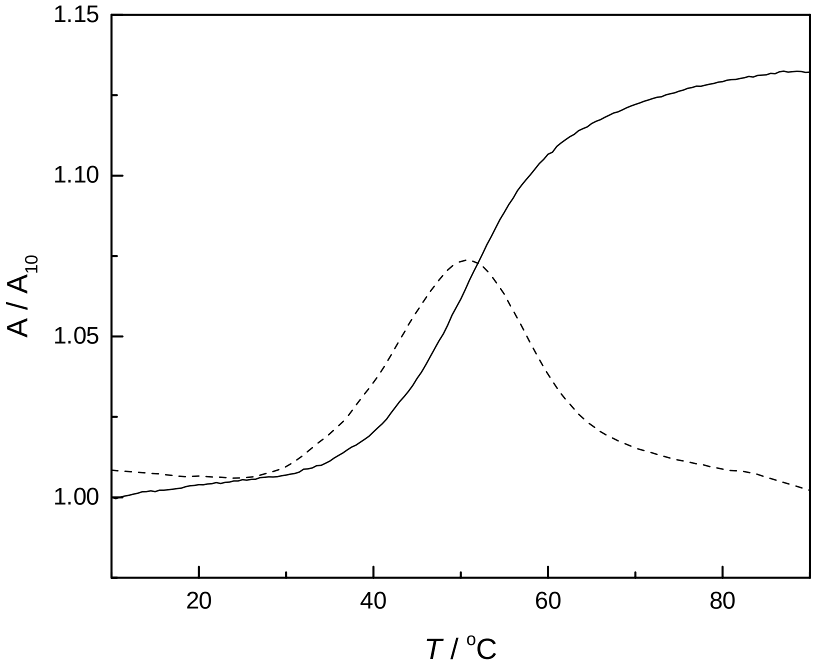

Figure S25. UV melting curve (solid line) and its first derivative (dashed line) of duplex ON1z-Hg$_1$b•ON2c; pH = 7.4 (20 mM cacodylate buffer); [oligonucleotides] = 1.0 µM; *I*(NaCl) = 0.10 M.

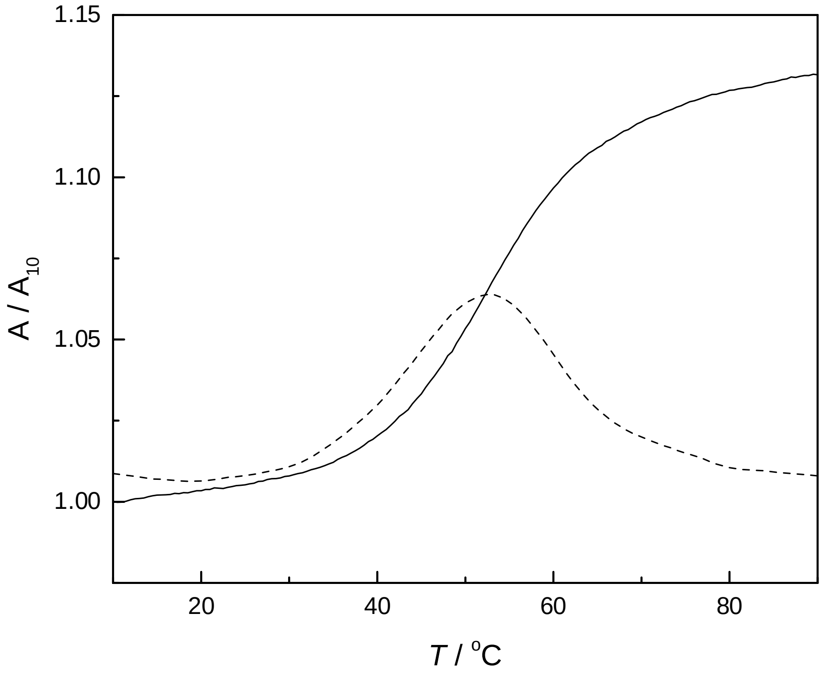

Figure S26. UV melting curve (solid line) and its first derivative (dashed line) of duplex ON1z-Hg$_1$b•ON2g; pH = 7.4 (20 mM cacodylate buffer); [oligonucleotides] = 1.0 µM; *I*(NaCl) = 0.10 M.

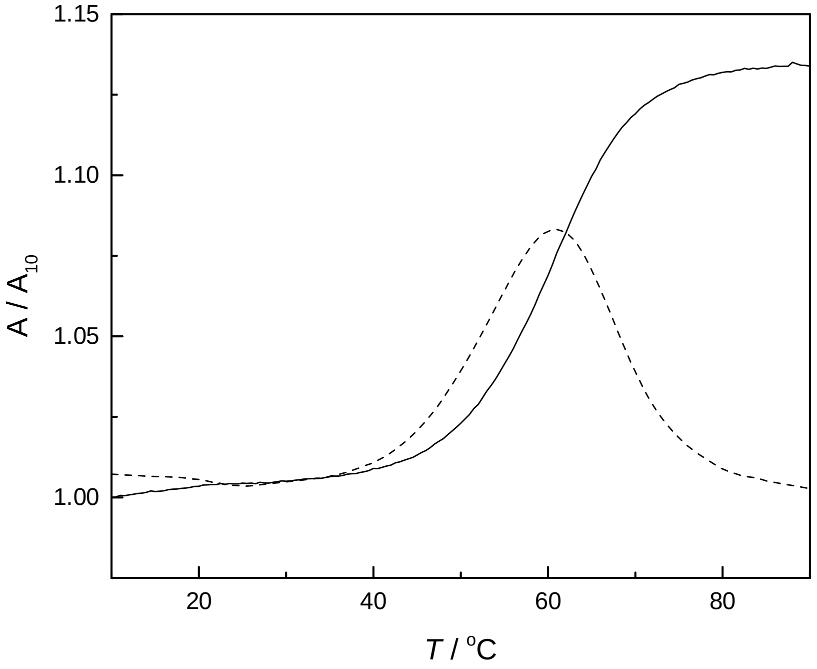

Figure S27. UV melting curve (solid line) and its first derivative (dashed line) of duplex ON1z-Hg$_1$b•ON2t; pH = 7.4 (20 mM cacodylate buffer); [oligonucleotides] = 1.0 µM; *I*(NaCl) = 0.10 M.

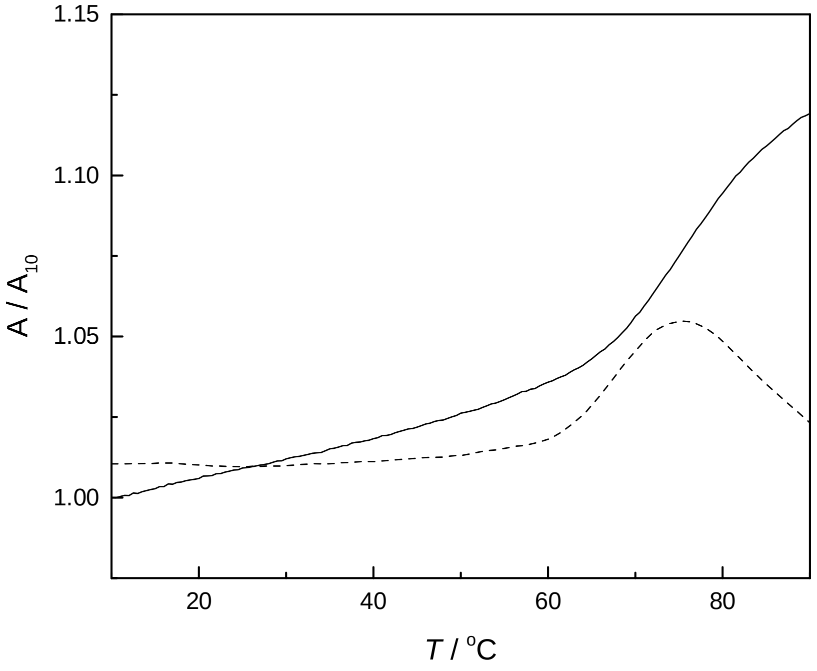

Figure S28. UV melting curve (solid line) and its first derivative (dashed line) of duplex ON1z-Hg$_1$b•ON2s^{2}t; pH = 7.4 (20 mM cacodylate buffer); [oligonucleotides] = 1.0 µM; *I*(NaCl) = 0.10 M.

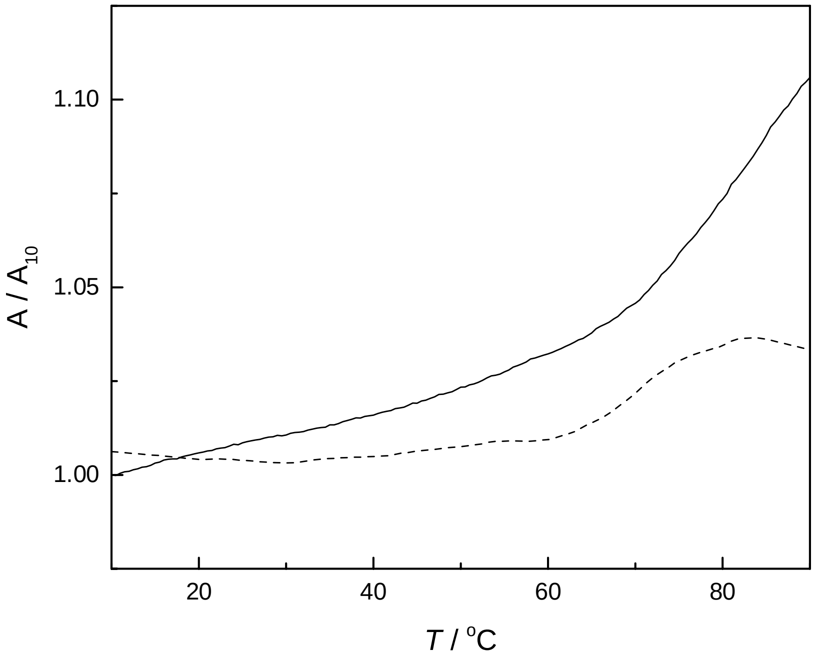

Figure S29. UV melting curve (solid line) and its first derivative (dashed line) of duplex ON1z-Hg$_1$b•ON2s^{4}t; pH = 7.4 (20 mM cacodylate buffer); [oligonucleotides] = 1.0 µM; *I*(NaCl) = 0.10 M.

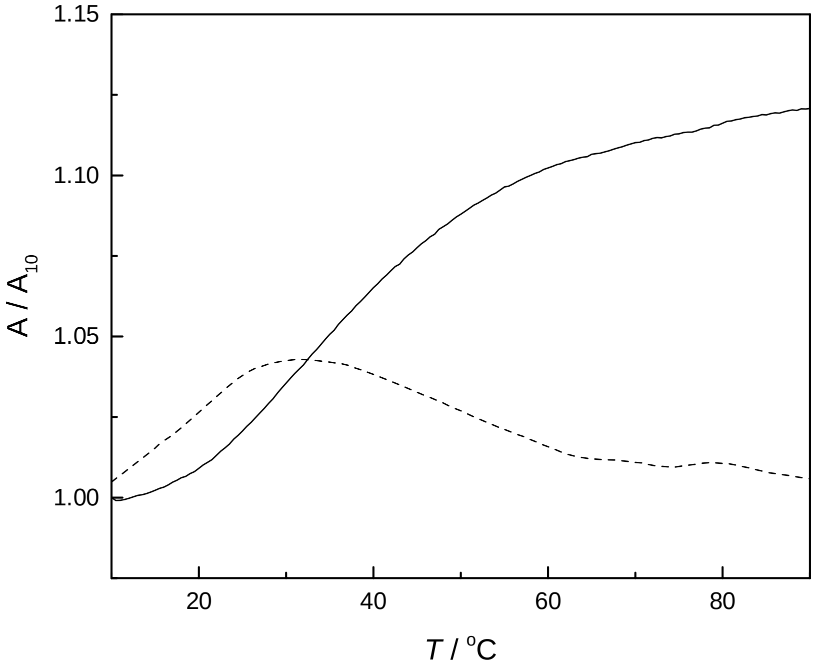

Figure S30. UV melting curve (solid line) and its first derivative (dashed line) of duplex ON1z-Hg$_2$•ON2a; pH = 7.4 (20 mM cacodylate buffer); [oligonucleotides] = 1.0 µM; *I*(NaCl) = 0.10 M.

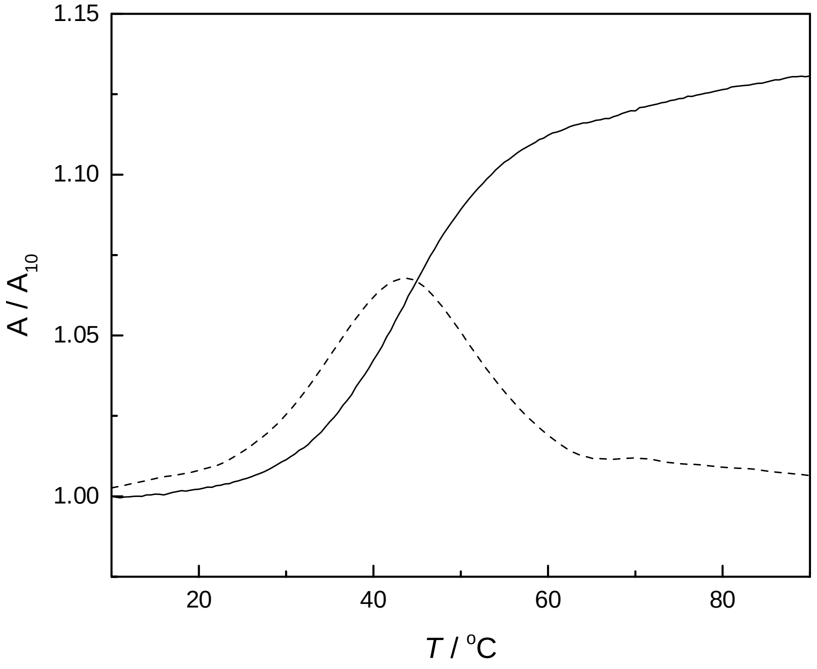

Figure S31. UV melting curve (solid line) and its first derivative (dashed line) of duplex ON1z-Hg$_2$•ON2c; pH = 7.4 (20 mM cacodylate buffer); [oligonucleotides] = 1.0 µM; *I*(NaCl) = 0.10 M.

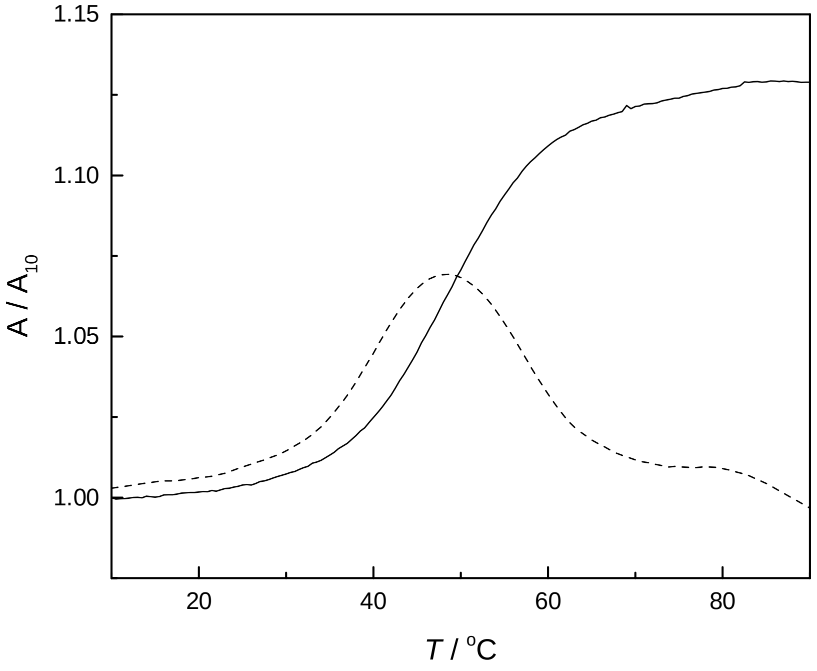

Figure S32. UV melting curve (solid line) and its first derivative (dashed line) of duplex ON1z-Hg_2•ON2g; pH = 7.4 (20 mM cacodylate buffer); [oligonucleotides] = 1.0 µM; *I*(NaCl) = 0.10 M.

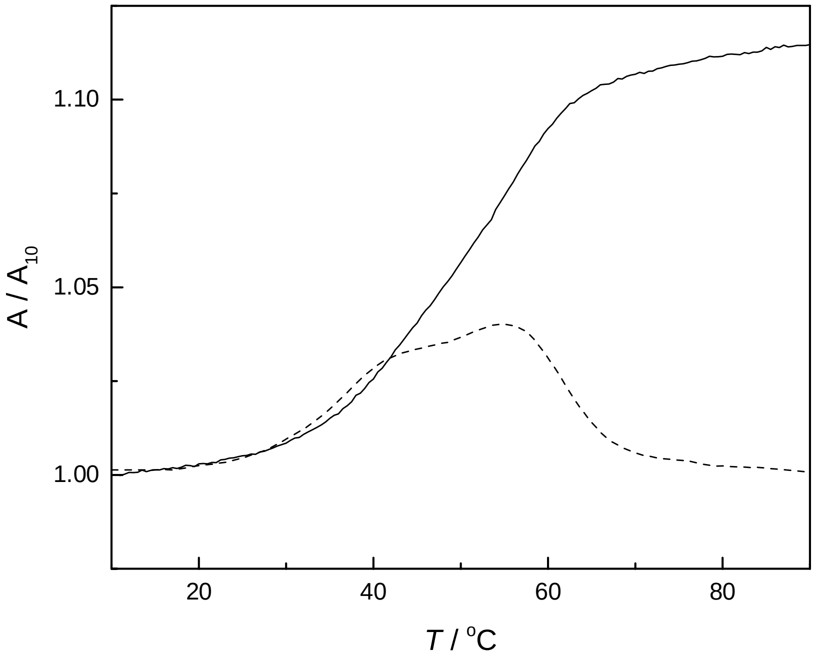

Figure S33. UV melting curve (solid line) and its first derivative (dashed line) of duplex ON1z-Hg_2•ON2t; pH = 7.4 (20 mM cacodylate buffer); [oligonucleotides] = 1.0 µM; *I*(NaCl) = 0.10 M.

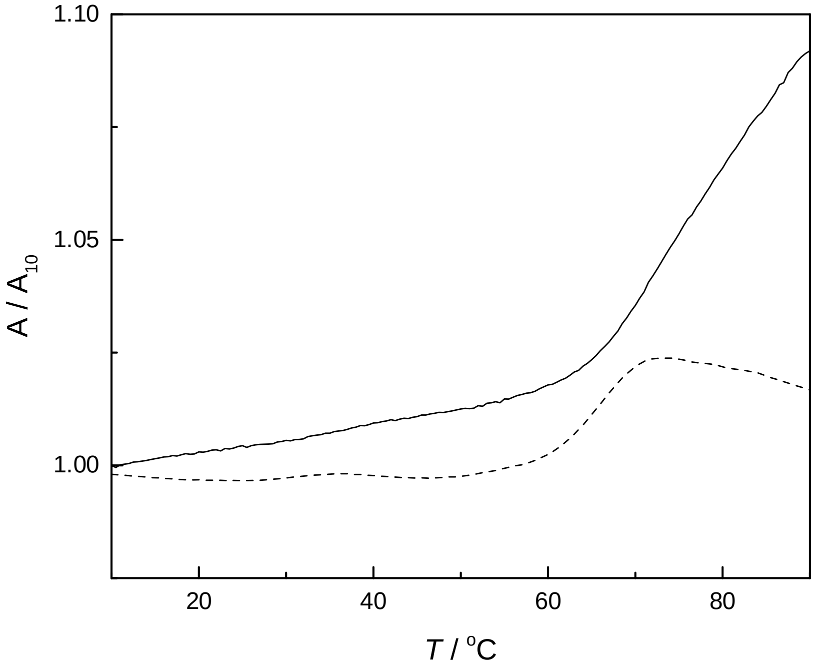

Figure S34. UV melting curve (solid line) and its first derivative (dashed line) of duplex ON1z-Hg_2•ON2s^{2}t; pH = 7.4 (20 mM cacodylate buffer); [oligonucleotides] = 1.0 μM; *I*(NaCl) = 0.10 M.

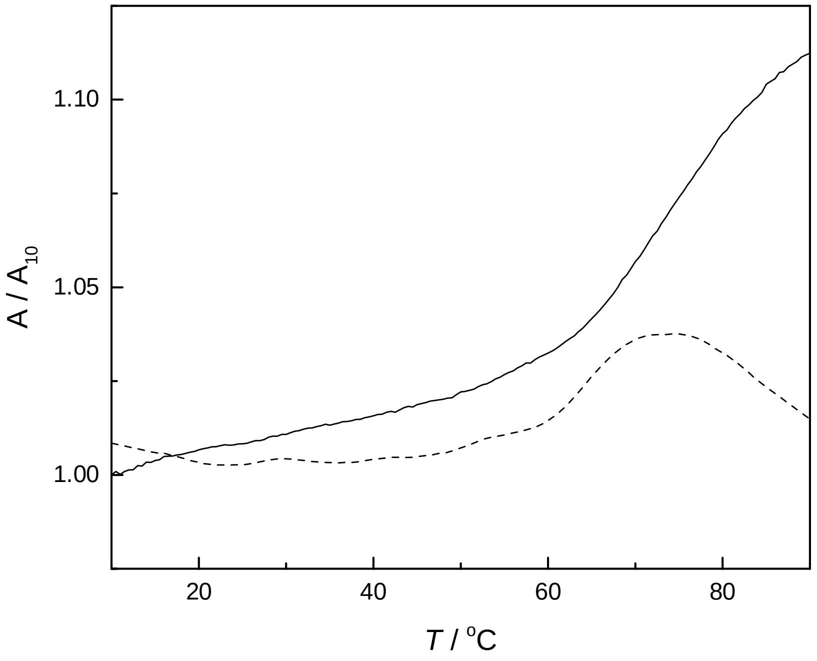

Figure S35. UV melting curve (solid line) and its first derivative (dashed line) of duplex ON1z-Hg_2•ON2s^{4}t; pH = 7.4 (20 mM cacodylate buffer); [oligonucleotides] = 1.0 μM; *I*(NaCl) = 0.10 M.

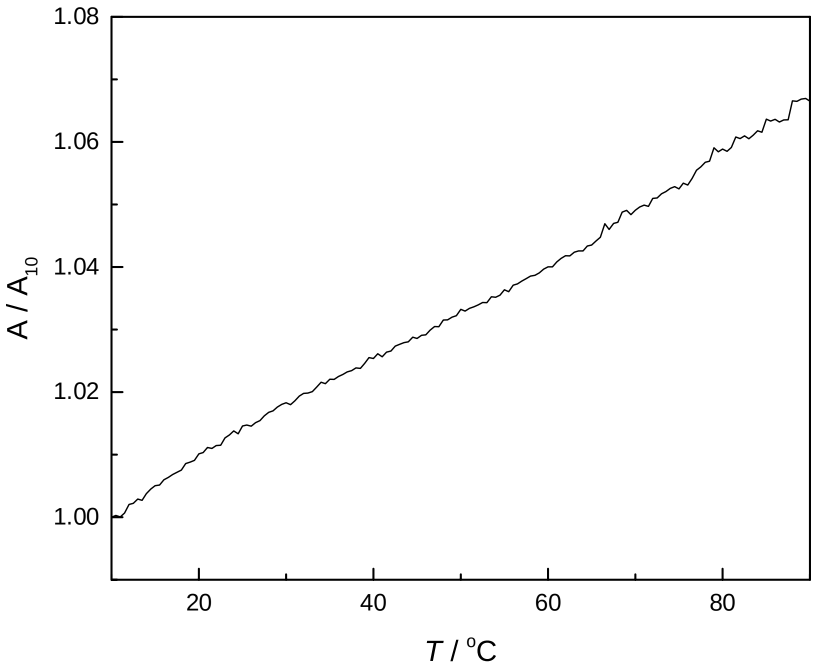

Figure S36. Temperature-dependent absorbance of oligonucleotide ON1z-Hg_1a; pH = 7.4 (20 mM cacodylate buffer); [oligonucleotides] = 1.0 µM; *I*(NaCl) = 0.10 M.

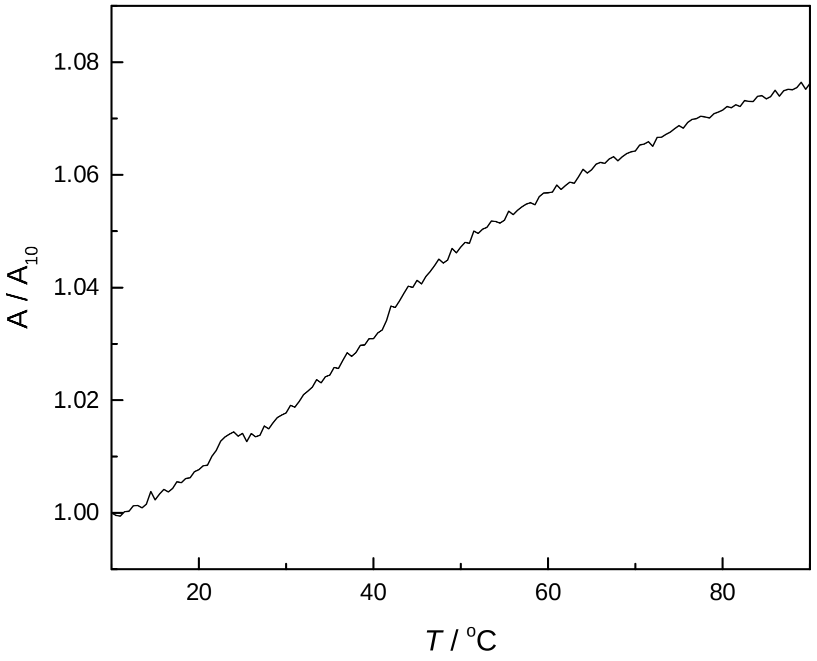

Figure S37. Temperature-dependent absorbance of oligonucleotide ON1z-Hg_2; pH = 7.4 (20 mM cacodylate buffer); [oligonucleotides] = 1.0 µM; *I*(NaCl) = 0.10 M.

Table S1. Melting temperatures of the duplexes studied; pH = 7.4 (20 mM cacodylate buffer); [oligonucleotides] = 1.0 µM; *I*(NaCl) = 0.10 M. The error limits represent standard errors of the average of three experiments.

	ON2a	ON2c	ON2g	ON2t	$ON2s^2t$	$ON2s^4t$
ON1a	38.3 ± 0.1	38.2 ± 0.1	44.1 ± 0.1	52.0 ± 0.1	53.5 ± 0.1	47.5 ± 0.1
ON1z	45.9 ± 0.2	49.4 ± 0.1	47.8 ± 0.1	48.3 ± 0.1	47.7 ± 0.1	47.7 ± 0.1
$ON1z\text{-}Hg_1a$	37.8 ± 0.1	43.3 ± 0.1	52.3 ± 0.1	60.0 ± 0.1	84.5 ± 0.4	76.5 ± 0.2
$ON1z\text{-}Hg_1b$	47.7 ± 0.1	50.3 ± 0.1	52.1 ± 0.1	60.2 ± 0.1	75.7 ± 0.2	82 ± 1
$ON1z\text{-}Hg_2$	33.3 ± 0.1	43.2 ± 0.1	48.3 ± 0.1	48.8 ± 0.1	77.1 ± 0.2	74.5 ± 0.3

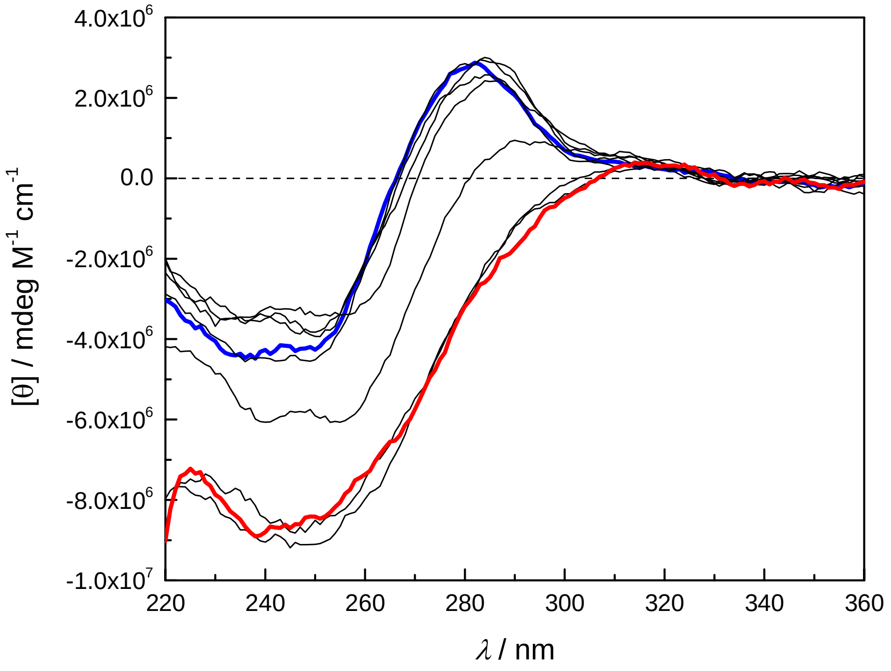

Figure S38. CD spectra of duplex ON1z•ON2a, recorded at 10 °C intervals between 10 and 90 °C (thick blue and red lines, respectively); pH = 7.4 (20 mM cacodylate buffer); [oligonucleotides] = 1.0 µM; *I*(NaCl) = 0.10 M.

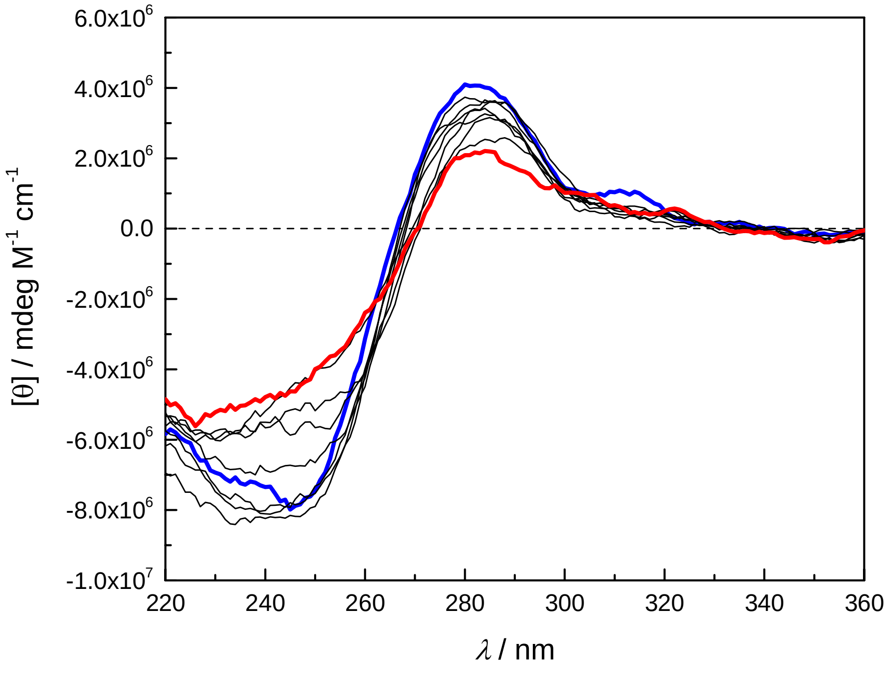

Figure S39. CD spectra of duplex ON1z•ON2c, recorded at 10 °C intervals between 10 and 90 °C (thick blue and red lines, respectively); pH = 7.4 (20 mM cacodylate buffer); [oligonucleotides] = 1.0 µM; *I*(NaCl) = 0.10 M.

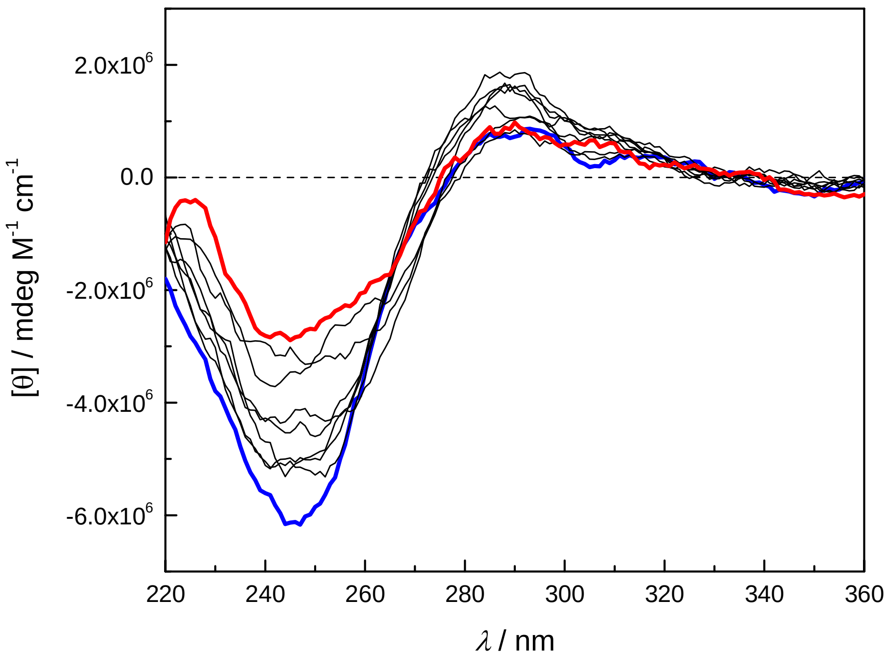

Figure S40. CD spectra of duplex ON1z•ON2g, recorded at 10 °C intervals between 10 and 90 °C (thick blue and red lines, respectively); pH = 7.4 (20 mM cacodylate buffer); [oligonucleotides] = 1.0 µM; *I*(NaCl) = 0.10 M.

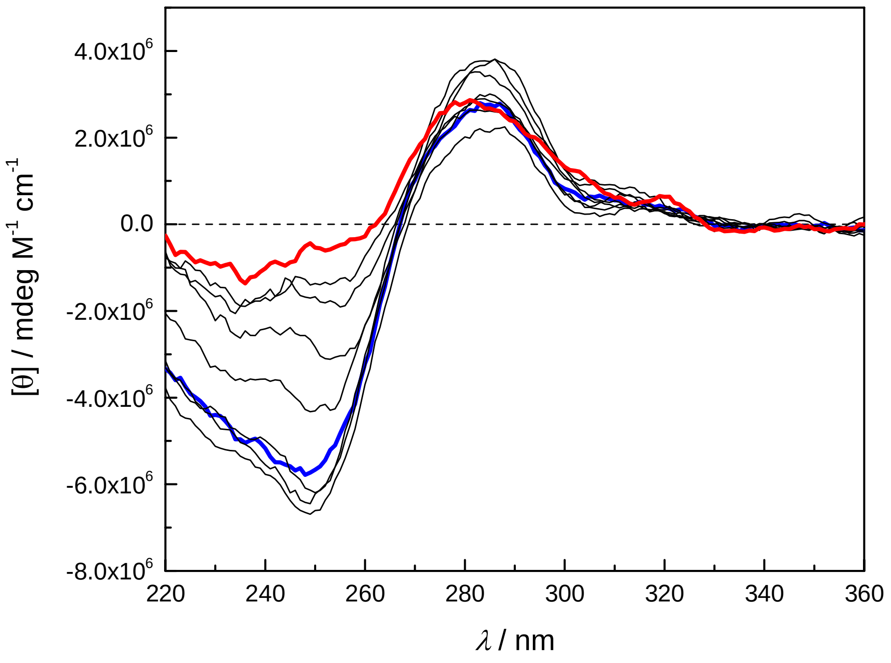

Figure S41. CD spectra of duplex ON1z•ON2t, recorded at 10 °C intervals between 10 and 90 °C (thick blue and red lines, respectively); pH = 7.4 (20 mM cacodylate buffer); [oligonucleotides] = 1.0 µM; *I*(NaCl) = 0.10 M.

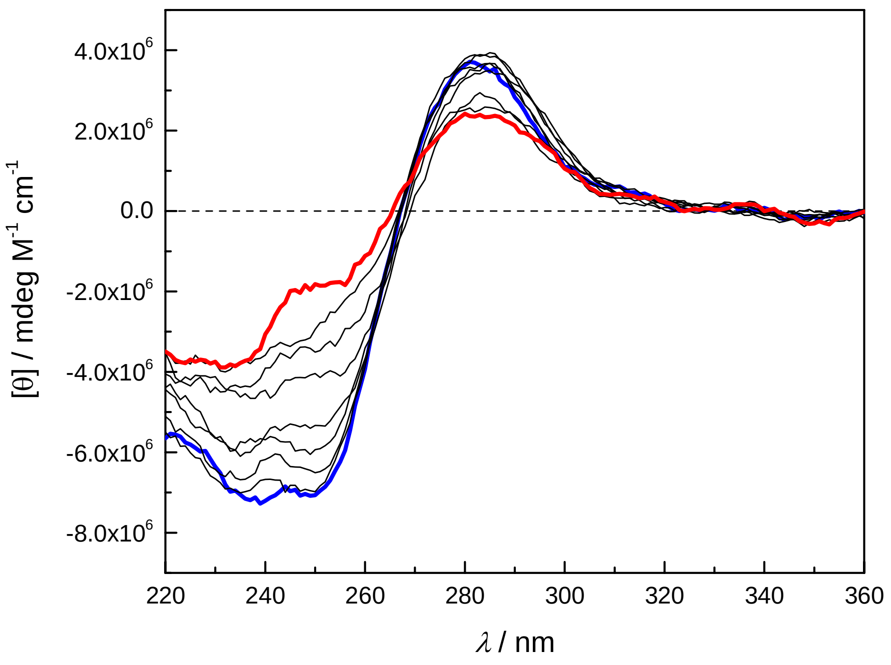

Figure S42. CD spectra of duplex ON1z•ON2s^{2}t, recorded at 10 °C intervals between 10 and 90 °C (thick blue and red lines, respectively); pH = 7.4 (20 mM cacodylate buffer); [oligonucleotides] = 1.0 μM; *I*(NaCl) = 0.10 M.

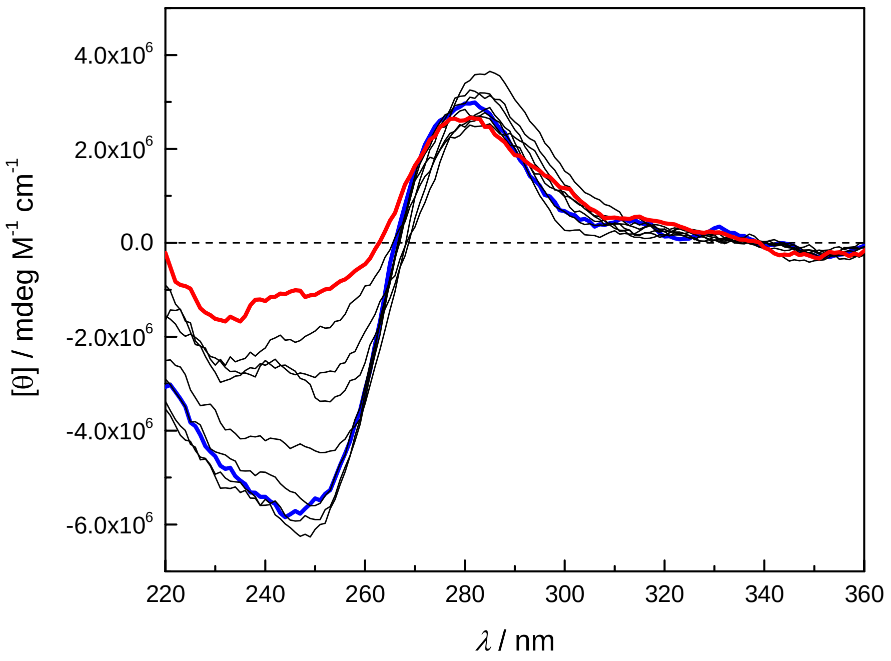

Figure S43. CD spectra of duplex ON1z•ON2s^{4}t, recorded at 10 °C intervals between 10 and 90 °C (thick blue and red lines, respectively); pH = 7.4 (20 mM cacodylate buffer); [oligonucleotides] = 1.0 μM; *I*(NaCl) = 0.10 M.

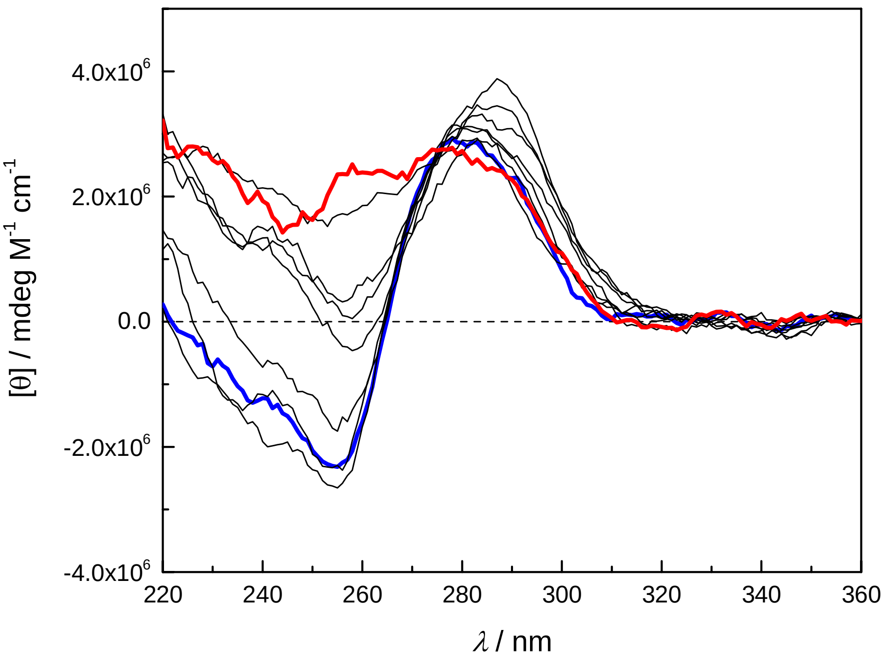

Figure S44. CD spectra of duplex ON1z-Hg$_1$a•ON2a, recorded at 10 °C intervals between 10 and 90 °C (thick blue and red lines, respectively); pH = 7.4 (20 mM cacodylate buffer); [oligonucleotides] = 1.0 µM; *I*(NaCl) = 0.10 M.

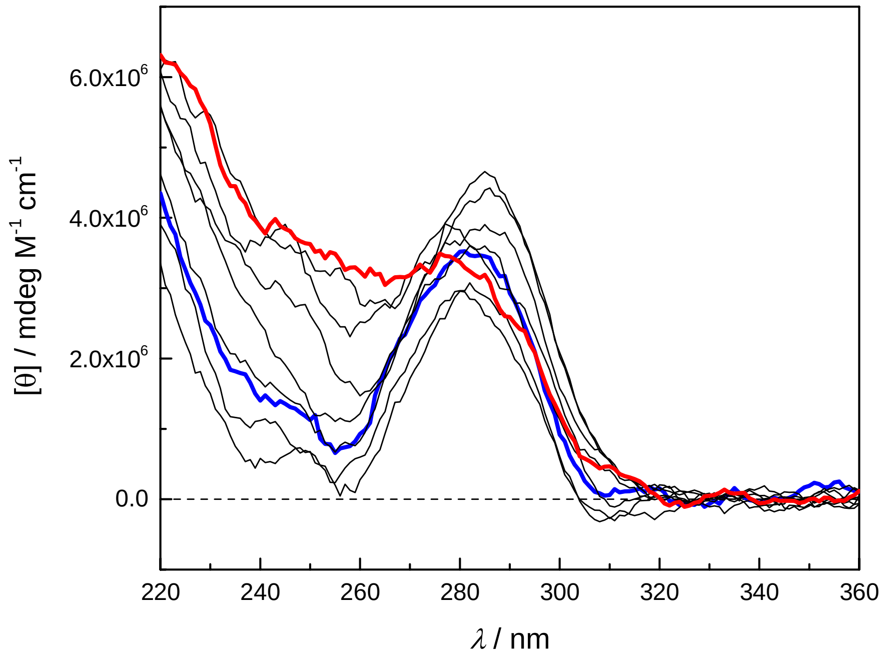

Figure S45. CD spectra of duplex ON1z-Hg$_1$a•ON2c, recorded at 10 °C intervals between 10 and 90 °C (thick blue and red lines, respectively); pH = 7.4 (20 mM cacodylate buffer); [oligonucleotides] = 1.0 µM; *I*(NaCl) = 0.10 M.

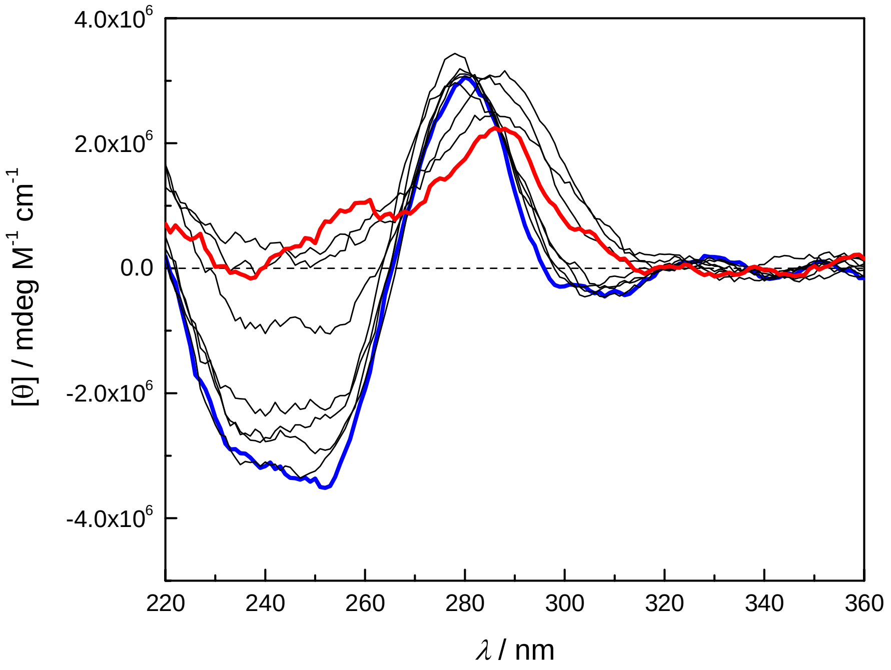

Figure S46. CD spectra of duplex ON1z-Hg$_1$a•ON2g, recorded at 10 °C intervals between 10 and 90 °C (thick blue and red lines, respectively); pH = 7.4 (20 mM cacodylate buffer); [oligonucleotides] = 1.0 μM; *I*(NaCl) = 0.10 M.

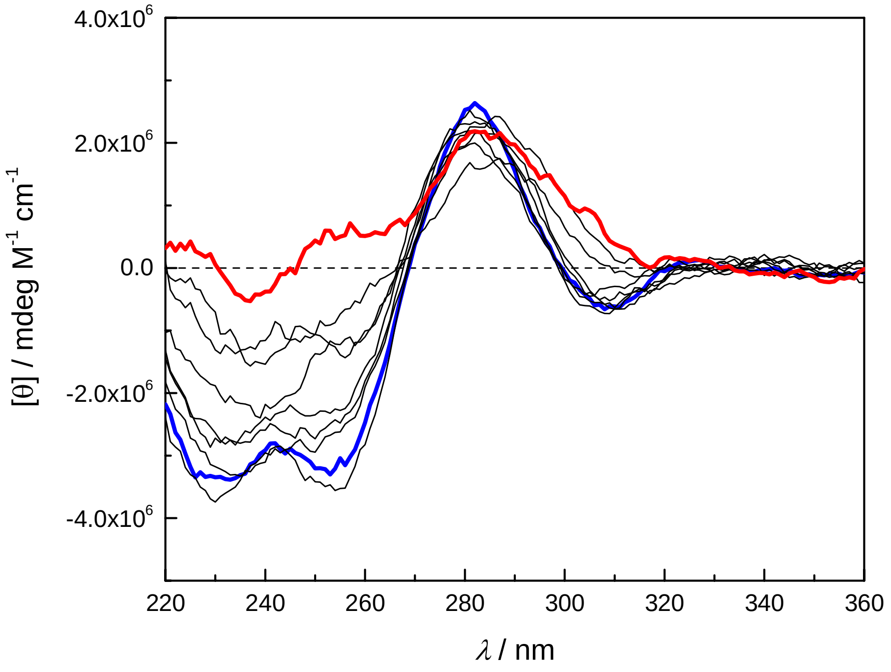

Figure S47. CD spectra of duplex ON1z-Hg$_1$a•ON2t, recorded at 10 °C intervals between 10 and 90 °C (thick blue and red lines, respectively); pH = 7.4 (20 mM cacodylate buffer); [oligonucleotides] = 1.0 μM; *I*(NaCl) = 0.10 M.

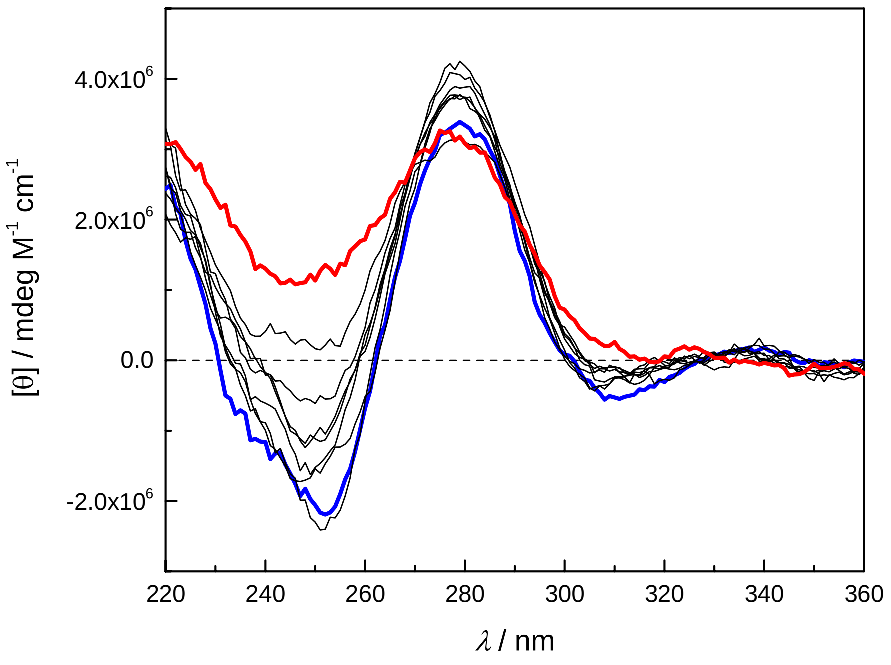

Figure S48. CD spectra of duplex ON1z-Hg$_1$a•ON2s^{2}t, recorded at 10 °C intervals between 10 and 90 °C (thick blue and red lines, respectively); pH = 7.4 (20 mM cacodylate buffer); [oligonucleotides] = 1.0 µM; *I*(NaCl) = 0.10 M.

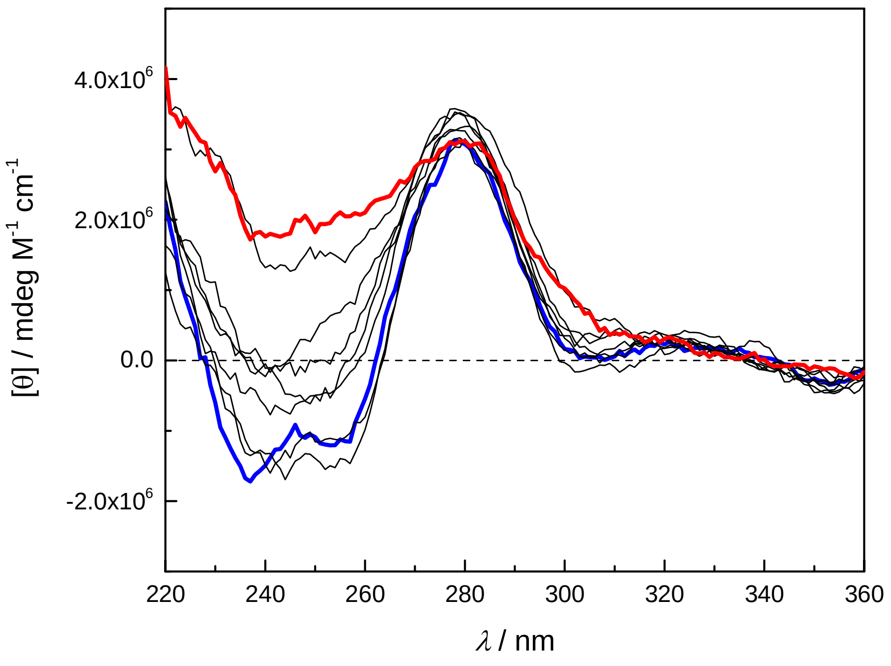

Figure S49. CD spectra of duplex ON1z-Hg$_1$a•ON2s^{4}t, recorded at 10 °C intervals between 10 and 90 °C (thick blue and red lines, respectively); pH = 7.4 (20 mM cacodylate buffer); [oligonucleotides] = 1.0 µM; *I*(NaCl) = 0.10 M.

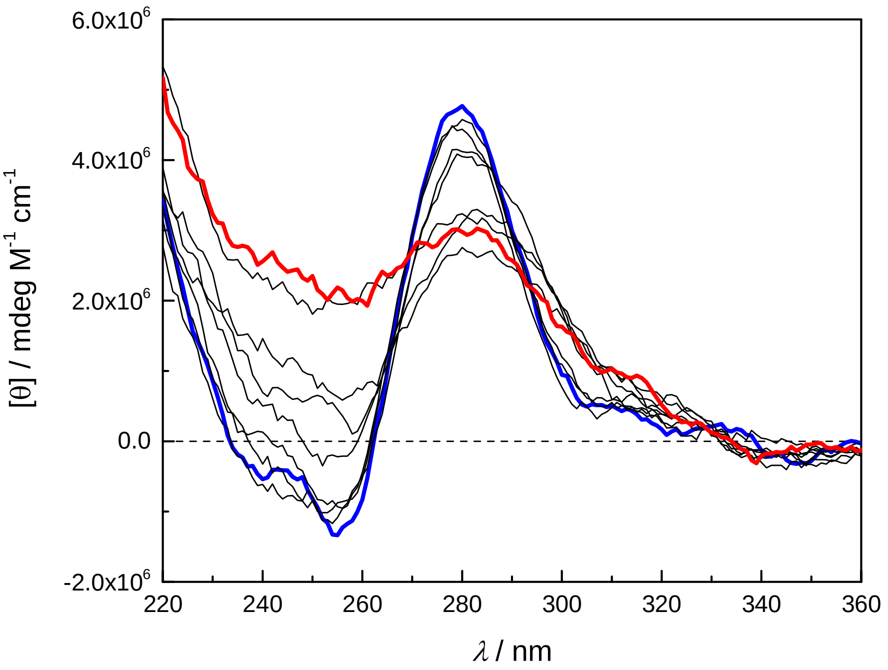

Figure S50. CD spectra of duplex ON1z-Hg$_1$b•ON2a, recorded at 10 °C intervals between 10 and 90 °C (thick blue and red lines, respectively); pH = 7.4 (20 mM cacodylate buffer); [oligonucleotides] = 1.0 μM; *I*(NaCl) = 0.10 M.

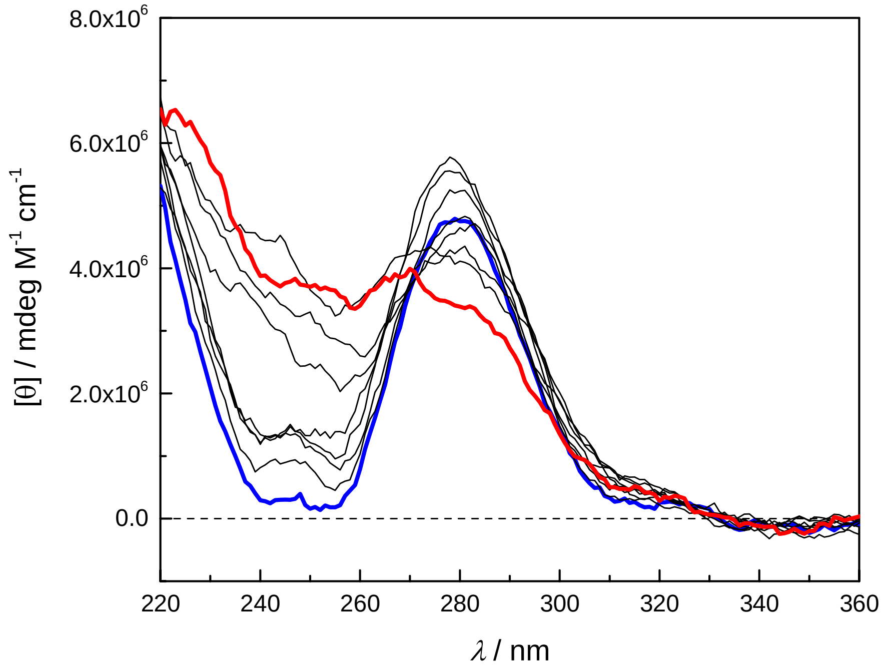

Figure S51. CD spectra of duplex ON1z-Hg$_1$b•ON2c, recorded at 10 °C intervals between 10 and 90 °C (thick blue and red lines, respectively); pH = 7.4 (20 mM cacodylate buffer); [oligonucleotides] = 1.0 μM; *I*(NaCl) = 0.10 M.

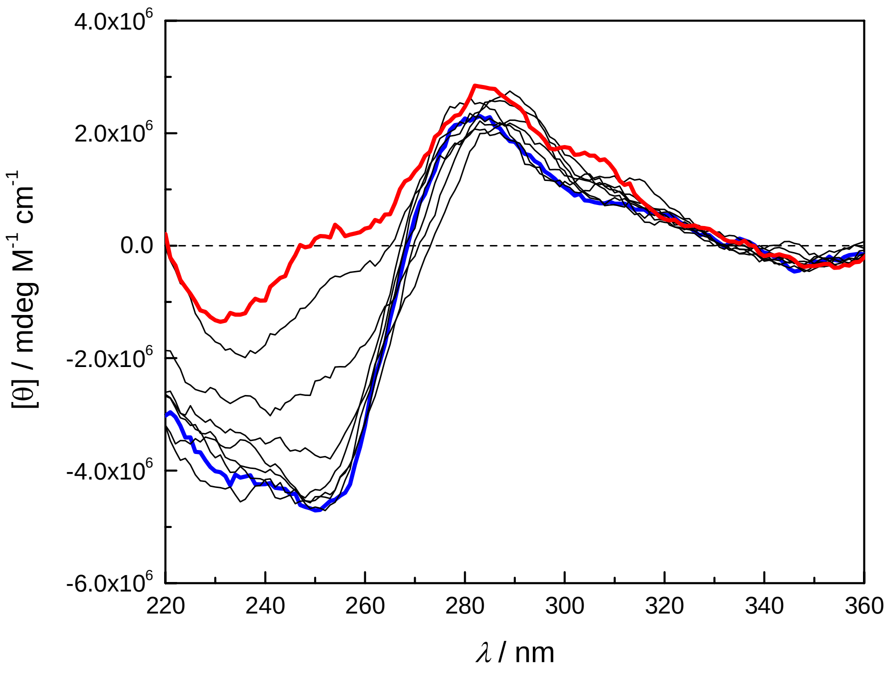

Figure S52. CD spectra of duplex ON1z-Hg$_1$b•ON2g, recorded at 10 °C intervals between 10 and 90 °C (thick blue and red lines, respectively); pH = 7.4 (20 mM cacodylate buffer); [oligonucleotides] = 1.0 μM; *I*(NaCl) = 0.10 M.

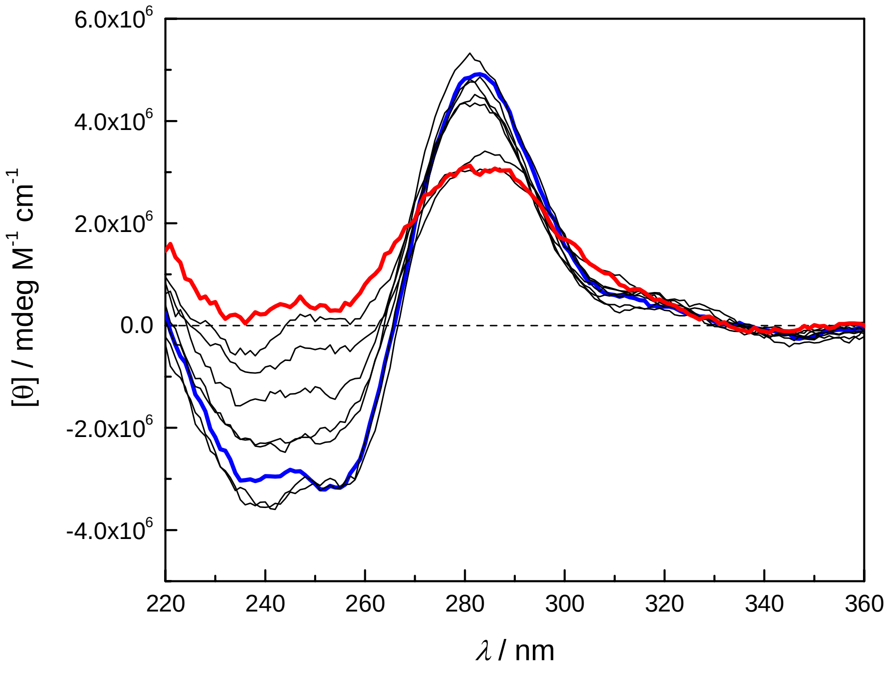

Figure S53. CD spectra of duplex ON1z-Hg$_1$b•ON2t, recorded at 10 °C intervals between 10 and 90 °C (thick blue and red lines, respectively); pH = 7.4 (20 mM cacodylate buffer); [oligonucleotides] = 1.0 μM; *I*(NaCl) = 0.10 M.

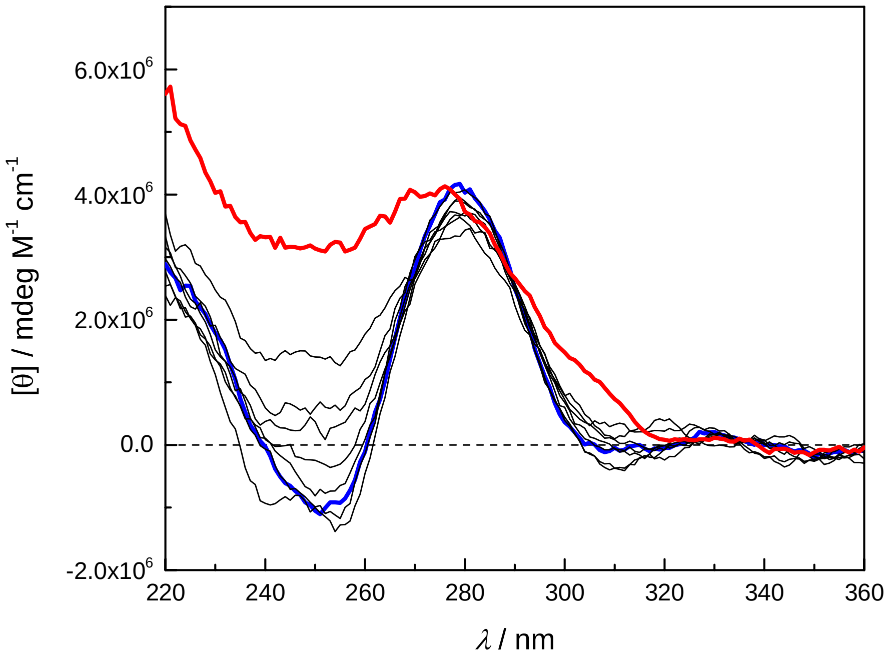

Figure S54. CD spectra of duplex ON1z-Hg_1b•ON2s^2t, recorded at 10 °C intervals between 10 and 90 °C (thick blue and red lines, respectively); pH = 7.4 (20 mM cacodylate buffer); [oligonucleotides] = 1.0 μM; *I*(NaCl) = 0.10 M.

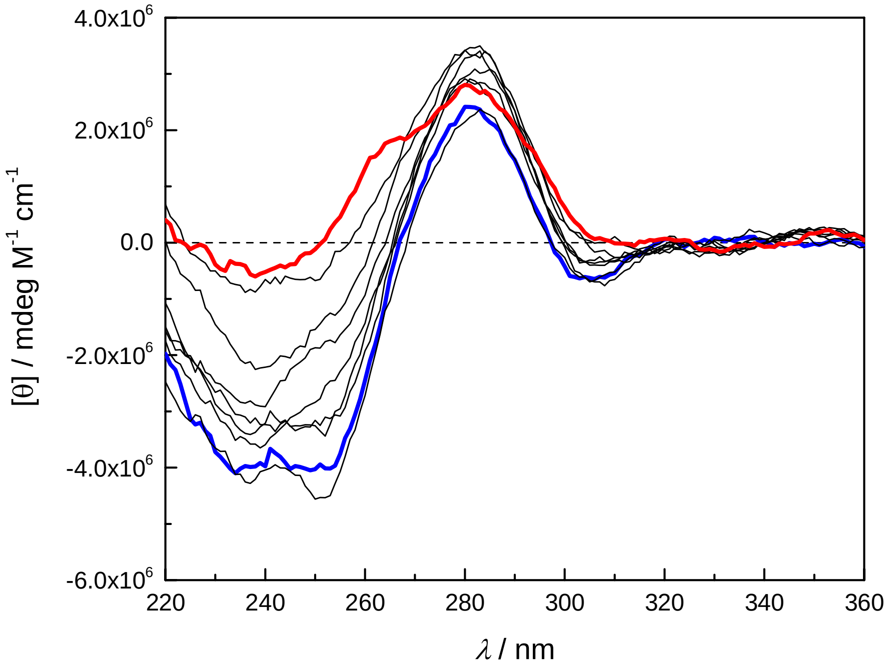

Figure S55. CD spectra of duplex ON1z-Hg_1b•ON2s^4t, recorded at 10 °C intervals between 10 and 90 °C (thick blue and red lines, respectively); pH = 7.4 (20 mM cacodylate buffer); [oligonucleotides] = 1.0 μM; *I*(NaCl) = 0.10 M.

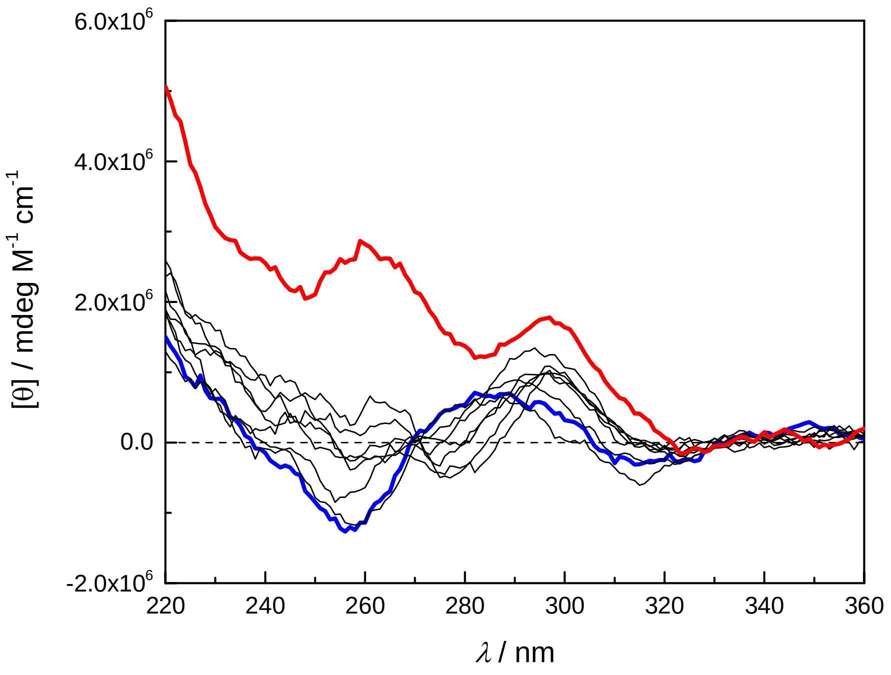

Figure S56. CD spectra of duplex ON1z-Hg_2•ON2a, recorded at 10 °C intervals between 10 and 90 °C (thick blue and red lines, respectively); pH = 7.4 (20 mM cacodylate buffer); [oligonucleotides] = 1.0 μM; *I*(NaCl) = 0.10 M.

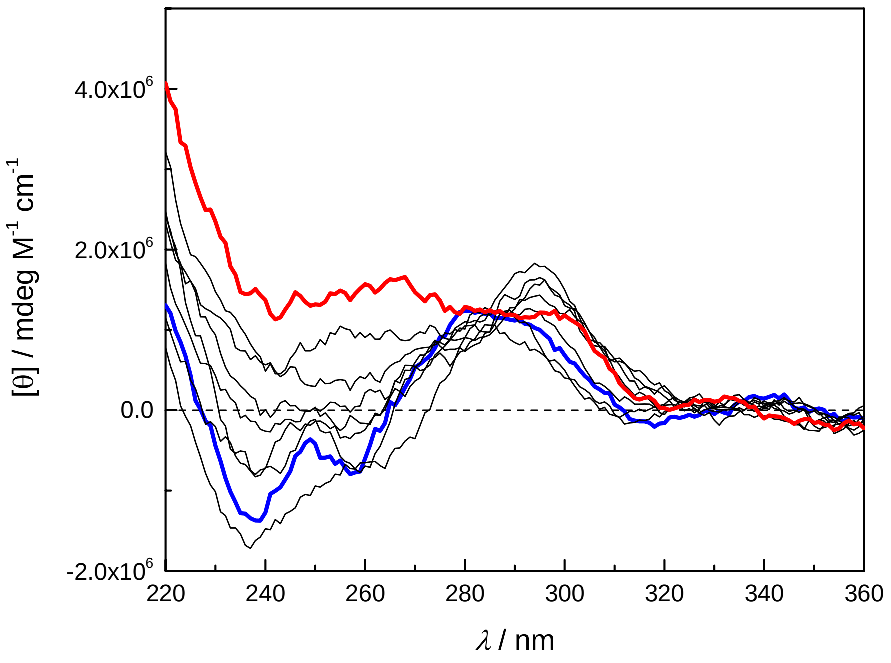

Figure S57. CD spectra of duplex ON1z-Hg_2•ON2c, recorded at 10 °C intervals between 10 and 90 °C (thick blue and red lines, respectively); pH = 7.4 (20 mM cacodylate buffer); [oligonucleotides] = 1.0 μM; *I*(NaCl) = 0.10 M.

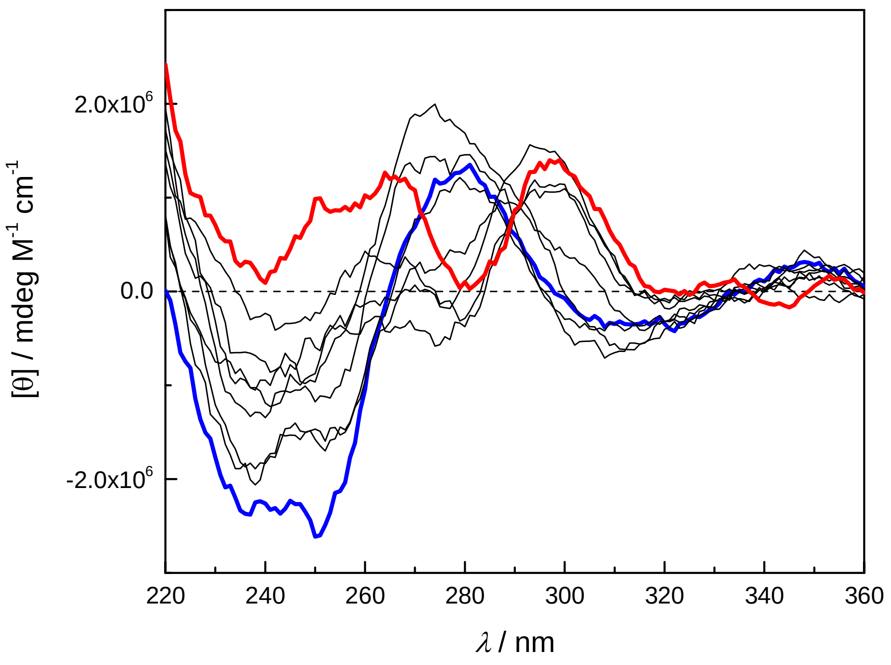

Figure S58. CD spectra of duplex ON1z-Hg_2•ON2g, recorded at 10 °C intervals between 10 and 90 °C (thick blue and red lines, respectively); pH = 7.4 (20 mM cacodylate buffer); [oligonucleotides] = 1.0 μM; *I*(NaCl) = 0.10 M.

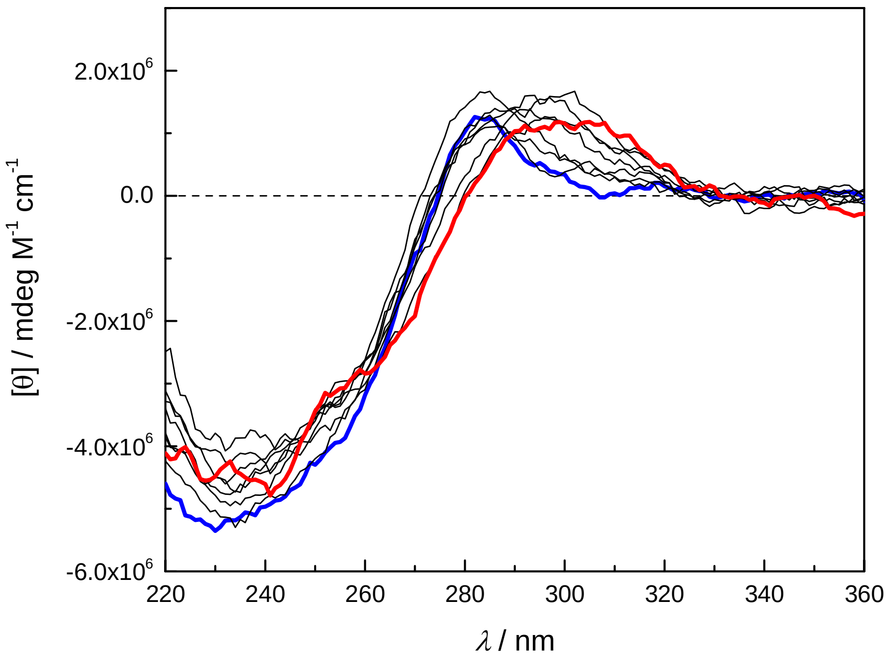

Figure S59. CD spectra of duplex ON1z-Hg_2•ON2t, recorded at 10 °C intervals between 10 and 90 °C (thick blue and red lines, respectively); pH = 7.4 (20 mM cacodylate buffer); [oligonucleotides] = 1.0 μM; *I*(NaCl) = 0.10 M.

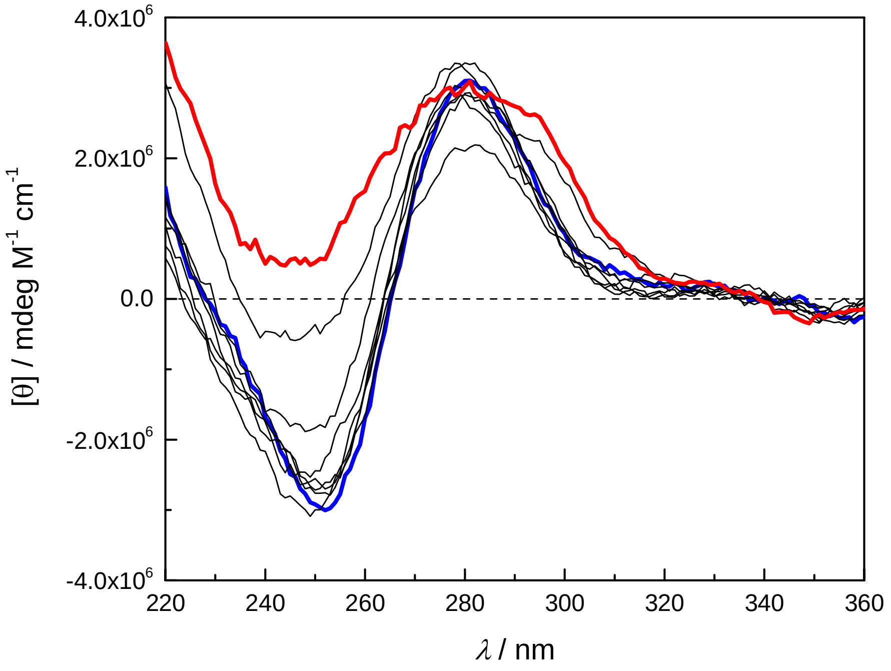

Figure S60. CD spectra of duplex ON1z-Hg_2•ON2s^{2}t, recorded at 10 °C intervals between 10 and 90 °C (thick blue and red lines, respectively); pH = 7.4 (20 mM cacodylate buffer); [oligonucleotides] = 1.0 µM; *I*(NaCl) = 0.10 M.

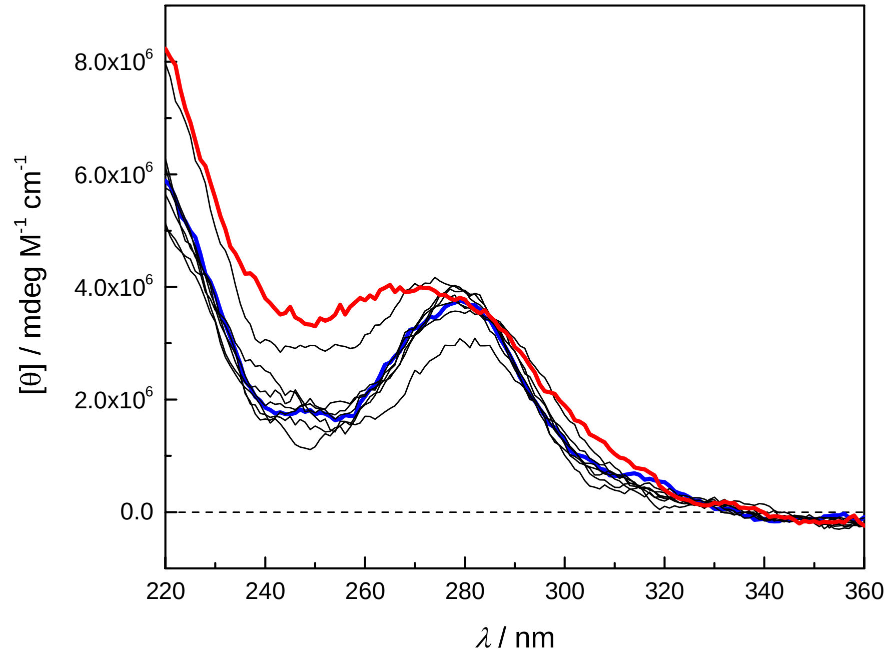

Figure S61. CD spectra of duplex ON1z-Hg_2•ON2s^{4}t, recorded at 10 °C intervals between 10 and 90 °C (thick blue and red lines, respectively); pH = 7.4 (20 mM cacodylate buffer); [oligonucleotides] = 1.0 µM; *I*(NaCl) = 0.10 M.

Table S2. Cartesian coordinates of the optimized (PBE0DH) Hg(II)-mediated base pairs between 1-mercuri-3-methylcarbazole and either 1-methyl-2-thiothymine or 1-methyl-4-thiothymine.

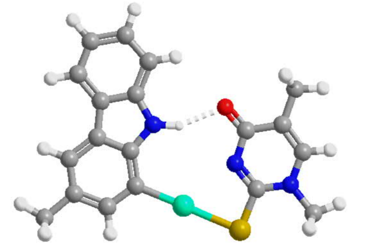

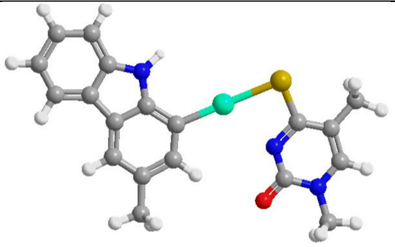

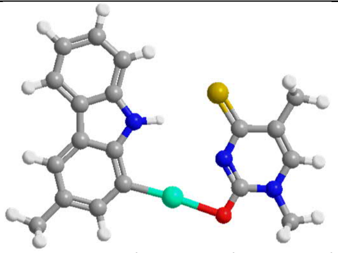

Atom	X / Å	Y / Å	Z / Å	Atom	X / Å	Y / Å	Z / Å	Atom	X / Å	Y / Å	Z / Å
C(1)	4.887	3.536	-0.000	C(1)	-7.154	-0.261	-0.000	C(1)	5.014	3.432	0.001
C(2)	3.644	4.183	-0.000	C(2)	-6.790	-1.616	-0.001	C(2)	3.787	4.109	0.001
C(3)	2.457	3.472	0.000	C(3)	-5.458	-2.008	-0.001	C(3)	2.583	3.427	0.001
C(4)	2.544	2.082	0.000	C(4)	-4.490	-1.005	-0.000	C(4)	2.634	2.036	0.000
C(5)	3.784	1.413	-0.000	C(5)	-4.836	0.368	0.000	C(5)	3.859	1.338	0.000
C(6)	4.966	2.155	-0.000	C(6)	-6.185	0.733	-0.000	C(6)	5.058	2.050	0.001
H(7)	5.794	4.126	-0.000	H(7)	-8.205	0.010	-0.000	H(7)	5.935	4.000	0.001
H(8)	3.611	5.265	-0.000	H(8)	-7.566	-2.377	-0.001	H(8)	3.780	5.191	0.001
H(9)	5.928	1.658	-0.000	H(9)	-6.471	1.781	0.000	H(9)	6.008	1.529	0.001
C(10)	3.484	0.006	-0.000	C(10)	-3.596	1.108	0.000	C(10)	3.527	-0.063	0.000
C(11)	4.273	-1.142	-0.000	C(11)	-3.282	2.469	0.000	C(11)	4.289	-1.228	0.000
C(12)	2.073	-0.106	0.000	C(12)	-2.552	0.153	0.000	C(12)	2.115	-0.142	0.000
C(13)	3.680	-2.395	0.000	C(13)	-1.953	2.879	0.000	C(13)	3.669	-2.469	-0.000
H(14)	5.353	-1.056	-0.000	H(14)	-4.077	3.212	0.001	H(14)	5.371	-1.167	0.000
C(15)	1.455	-1.362	0.000	C(15)	-1.209	0.537	0.000	C(15)	1.475	-1.385	-0.000
C(16)	2.278	-2.483	0.000	C(16)	-0.934	1.904	0.000	C(16)	2.266	-2.528	-0.000
H(17)	1.834	-3.472	0.000	H(17)	0.100	2.244	0.000	H(17)	1.798	-3.505	-0.000
N(18)	1.530	1.155	0.000	N(18)	-3.115	-1.108	0.000	N(18)	1.599	1.130	0.000
H(19)	0.550	1.423	0.000	H(19)	-2.597	-1.971	-0.001	H(19)	0.623	1.407	0.000
Hg(20)	-0.588	-1.530	0.000	Hg(20)	0.396	-0.761	0.000	Hg(20)	-0.555	-1.467	-0.000
C(21)	-4.450	1.922	-0.000	C(21)	5.537	0.125	-0.000	C(21)	-4.871	1.028	-0.000
C(22)	-3.147	-0.028	0.000	C(22)	3.572	1.399	-0.030	C(22)	-3.114	-0.515	-0.000
C(23)	-2.053	2.053	0.000	C(23)	3.458	-0.904	-0.126	C(23)	-2.589	1.774	-0.000
C(24)	-3.356	2.708	0.000	C(24)	4.816	-1.034	0.000	C(24)	-4.011	2.071	-0.000
H(25)	-5.453	2.327	-0.000	H(25)	6.624	0.118	-0.000	H(25)	-5.944	1.164	-0.000
N(26)	-2.043	0.677	0.000	N(26)	2.833	0.293	-0.116	N(26)	-2.230	0.465	-0.000
N(27)	-4.377	0.547	-0.000	N(27)	4.957	1.349	-0.000	N(27)	-4.450	-0.279	-0.000
C(28)	-3.412	4.196	0.000	C(28)	5.463	-2.383	0.000	C(28)	-4.498	3.480	-0.000
H(29)	-2.895	4.593	0.874	H(29)	5.169	-2.964	0.881	H(29)	-4.121	4.013	0.873
H(30)	-2.895	4.593	-0.874	H(30)	5.169	-2.964	-0.880	H(30)	-4.121	4.012	-0.874
H(31)	-4.438	4.558	0.000	H(31)	6.553	-2.293	-0.000	H(31)	-5.587	3.520	-0.000
C(32)	-5.587	-0.257	-0.000	C(32)	5.740	2.574	-0.001	C(32)	-5.375	-1.396	-0.000
H(33)	-5.622	-0.888	-0.886	H(33)	5.498	3.169	-0.884	H(33)	-5.221	-2.012	-0.884
H(34)	-5.622	-0.888	0.886	H(34)	5.498	3.170	0.882	H(34)	-5.221	-2.012	0.883
H(35)	-6.444	0.411	-0.000	H(35)	6.801	2.319	-0.000	H(35)	-6.388	-1.005	-0.000
C(36)	4.506	-3.648	0.000	C(36)	-1.585	4.337	0.001	C(36)	4.469	-3.740	-0.000
H(37)	4.301	-4.262	0.879	H(37)	-0.987	4.598	0.880	H(37)	4.251	-4.349	0.879
H(38)	4.301	-4.262	-0.879	H(38)	-0.987	4.598	-0.880	H(38)	4.251	-4.349	-0.879
H(39)	5.570	-3.416	0.000	H(39)	-2.476	4.972	0.001	H(39)	5.537	-3.530	-0.000
S(40)	-3.020	-1.765	-0.000	H(40)	-5.183	-3.059	-0.001	H(40)	1.636	3.950	0.001
O(41)	-0.995	2.681	0.000	S(41)	2.337	-2.282	-0.384	S(41)	-1.422	2.967	-0.000
H(42)	1.494	3.965	0.000	O(42)	3.025	2.470	0.080	O(42)	-2.700	-1.731	0.000

Table S3. Cartesian coordinates of the optimized (PBE0DH) Hg(II)-mediated base pairs between 8-mercuri-3-methylcarbazole and either 1-methyl-2-thiothymine or 1-methyl-4-thiothymine.

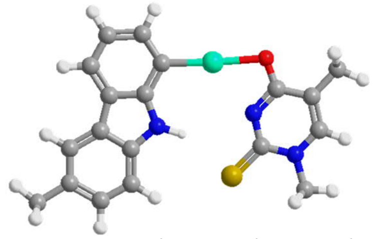

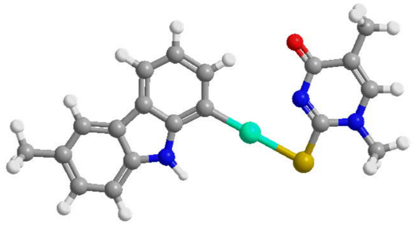

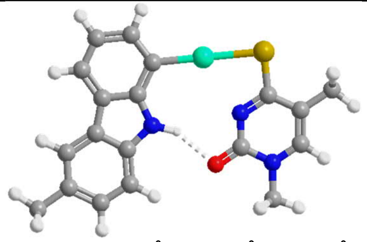

Atom	X / Å	Y / Å	Z / Å	Atom	X / Å	Y / Å	Z / Å	Atom	X / Å	Y / Å	Z / Å
C(1)	-2.845	-3.514	-0.001	C(1)	1.688	3.023	0.004	C(1)	-2.763	-3.539	-0.001
C(2)	-1.470	-3.261	-0.001	C(2)	0.664	2.068	0.004	C(2)	-1.390	-3.270	-0.001
C(3)	-0.971	-1.963	-0.000	C(3)	0.946	0.705	0.003	C(3)	-0.897	-1.968	-0.000
C(4)	-1.891	-0.910	-0.000	C(4)	2.289	0.331	0.002	C(4)	-1.834	-0.928	-0.000
C(5)	-3.287	-1.160	-0.000	C(5)	3.332	1.283	0.001	C(5)	-3.228	-1.191	-0.000
C(6)	-3.755	-2.470	-0.001	C(6)	3.018	2.640	0.003	C(6)	-3.684	-2.506	-0.001
H(7)	-3.195	-4.538	-0.001	H(7)	1.427	4.073	0.005	H(7)	-3.101	-4.567	-0.001
H(8)	-0.789	-4.102	-0.001	H(8)	-0.367	2.406	0.005	H(8)	-0.702	-4.105	-0.001
H(9)	-4.819	-2.670	-0.001	H(9)	3.804	3.385	0.003	H(9)	-4.746	-2.717	-0.001
C(10)	-3.934	0.126	0.000	C(10)	4.570	0.543	-0.000	C(10)	-3.889	0.087	0.000
C(11)	-5.268	0.538	-0.000	C(11)	5.915	0.910	-0.001	C(11)	-5.226	0.488	0.000
C(12)	-2.908	1.087	0.000	C(12)	4.223	-0.821	-0.001	C(12)	-2.872	1.058	0.000
C(13)	-5.571	1.889	0.000	C(13)	6.899	-0.067	-0.003	C(13)	-5.540	1.838	0.000
H(14)	-6.066	-0.195	-0.000	H(14)	6.194	1.957	-0.000	H(14)	-6.019	-0.251	-0.000
C(15)	-3.190	2.450	0.001	C(15)	5.194	-1.816	-0.002	C(15)	-3.165	2.419	0.001
C(16)	-4.520	2.825	0.001	C(16)	6.519	-1.420	-0.003	C(16)	-4.498	2.783	0.001
H(17)	-4.765	3.881	0.001	H(17)	7.293	-2.179	-0.005	H(17)	-4.753	3.837	0.001
N(18)	-1.688	0.447	0.000	N(18)	2.850	-0.926	0.001	N(18)	-1.647	0.432	0.000
H(19)	-0.804	0.942	0.000	H(19)	2.333	-1.785	-0.002	H(19)	-0.774	0.945	-0.000
Hg(20)	1.020	-1.561	0.000	Hg(20)	-0.637	-0.600	0.002	Hg(20)	1.116	-1.573	0.000
C(21)	4.571	1.980	0.000	C(21)	-5.708	0.197	-0.002	C(21)	3.851	2.863	-0.000
C(22)	2.189	2.111	-0.001	C(22)	-3.537	-0.729	-0.259	C(22)	1.530	2.292	-0.001
C(23)	3.275	0.028	0.000	C(23)	-3.664	1.543	0.043	C(23)	3.167	0.615	0.000
C(24)	4.575	0.624	0.000	C(24)	-5.201	1.444	-0.003	C(24)	4.239	1.560	0.000
H(25)	5.492	2.546	0.000	H(25)	-6.772	0.004	-0.003	H(25)	4.572	3.670	-0.000
N(26)	2.171	0.768	-0.000	N(26)	-2.921	0.362	-0.196	N(26)	1.899	0.985	-0.000
N(27)	3.431	2.723	-0.000	N(27)	-4.895	-0.961	0.033	N(27)	2.550	3.240	-0.001
C(28)	5.810	-0.210	0.001	C(28)	-6.043	2.671	-0.003	C(28)	5.674	1.149	0.001
H(29)	5.838	-0.858	0.876	H(29)	-5.817	3.283	0.869	H(29)	5.908	0.545	0.878
H(30)	5.838	-0.858	-0.875	H(30)	-5.817	3.283	-0.877	H(30)	5.909	0.545	-0.877
H(31)	6.703	0.413	0.001	H(31)	-7.105	2.429	-0.003	H(31)	6.327	2.020	0.001
C(32)	3.513	4.174	-0.000	C(32)	-5.504	-2.253	-0.000	C(32)	2.152	4.635	-0.001
H(33)	3.014	4.572	-0.881	H(33)	-5.194	-2.809	-0.884	H(33)	1.550	4.846	-0.882
H(34)	3.013	4.572	0.879	H(34)	-5.201	-2.805	0.888	H(34)	1.550	4.846	0.880
H(35)	4.561	4.461	0.000	H(35)	-6.585	-2.147	-0.005	H(35)	3.043	5.256	-0.001
C(36)	-6.994	2.365	0.000	C(36)	8.355	0.299	-0.004	C(36)	-6.968	2.302	0.000
H(37)	-7.210	2.976	0.879	H(37)	8.867	-0.099	0.874	H(37)	-7.190	2.910	0.879
H(38)	-7.210	2.976	-0.878	H(38)	8.865	-0.098	-0.883	H(38)	-7.190	2.911	-0.878
H(39)	-7.690	1.527	-0.000	H(39)	8.489	1.380	-0.003	H(39)	-7.656	1.457	-0.000
S(40)	0.779	3.013	-0.001	S(40)	-2.523	-2.154	0.001	H(40)	-2.372	3.155	0.001
O(41)	3.130	-1.252	0.000	O(41)	-3.120	2.617	-0.049	O(41)	0.358	2.659	-0.001
H(42)	-2.397	3.185	0.001	H(42)	4.928	-2.866	-0.003	S(42)	3.496	-1.090	0.001

Table S4. Cartesian coordinates of the optimized (PBE0DH) Hg(II)-mediated base pairs between 1,8-dimercuri-3-methylcarbazole and either 1-methyl-2-thiothymine or 1-methyl-4-thiothymine.

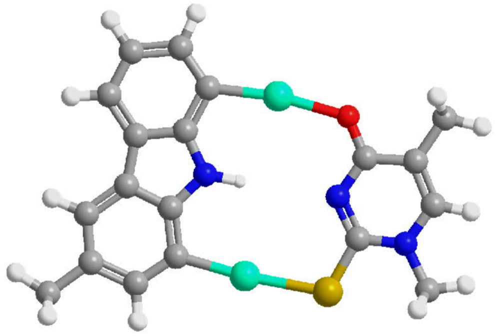

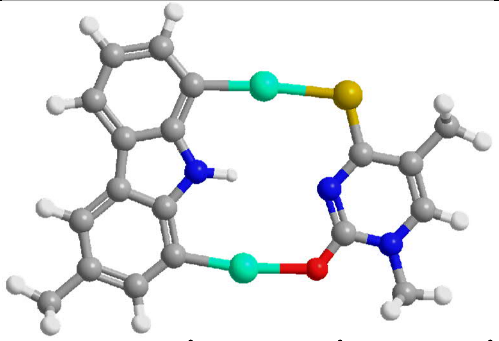

Atom	X / Å	Y / Å	Z / Å	Atom	X / Å	Y / Å	Z / Å
C(1)	2.963	4.406	-0.000	C(1)	2.788	4.473	-0.000
C(2)	1.564	4.314	0.000	C(2)	1.395	4.316	-0.000
C(3)	0.957	3.067	0.000	C(3)	0.830	3.048	-0.000
C(4)	1.779	1.946	0.000	C(4)	1.709	1.972	-0.000
C(5)	3.187	2.014	-0.000	C(5)	3.112	2.098	-0.000
C(6)	3.773	3.279	-0.000	C(6)	3.648	3.385	-0.000
H(7)	3.417	5.387	-0.000	H(7)	3.198	5.474	-0.001
H(8)	0.975	5.220	0.000	H(8)	0.771	5.199	-0.000
H(9)	4.850	3.389	-0.000	H(9)	4.719	3.540	-0.001
C(10)	3.659	0.643	-0.000	C(10)	3.644	0.748	-0.000
C(11)	4.903	0.012	-0.000	C(11)	4.913	0.171	-0.000
C(12)	2.511	-0.168	0.000	C(12)	2.529	-0.110	-0.000
C(13)	4.983	-1.376	-0.000	C(13)	5.059	-1.213	-0.000
H(14)	5.814	0.597	-0.000	H(14)	5.797	0.796	-0.001
C(15)	2.553	-1.556	-0.000	C(15)	2.649	-1.494	0.000
C(16)	3.805	-2.150	-0.000	C(16)	3.920	-2.042	-0.000
H(17)	3.902	-3.228	-0.000	H(17)	4.060	-3.115	-0.000
N(18)	1.395	0.631	0.000	N(18)	1.382	0.639	0.000
H(19)	0.446	0.298	0.000	H(19)	0.444	0.276	0.000
Hg(20)	-1.000	2.533	0.000	Hg(20)	-1.140	2.467	0.000
Hg(21)	0.683	-2.401	0.000	Hg(21)	0.824	-2.380	0.000
C(22)	-4.920	-1.182	-0.000	C(22)	-4.551	-2.150	-0.000
C(23)	-2.652	-1.820	-0.000	C(23)	-2.202	-2.194	-0.000
C(24)	-3.196	0.428	0.000	C(24)	-3.285	-0.154	-0.000
C(25)	-4.597	0.130	-0.000	C(25)	-4.566	-0.792	-0.000
H(26)	-5.945	-1.523	-0.000	H(26)	-5.460	-2.735	-0.000
N(27)	-2.266	-0.550	0.000	O(27)	-1.180	-2.963	0.000
N(28)	-3.972	-2.162	-0.000	N(28)	-2.144	-0.859	-0.000
C(29)	-5.605	1.226	-0.000	N(29)	-3.395	-2.855	-0.000
H(30)	-5.479	1.861	0.876	C(30)	-5.855	-0.038	-0.000
H(31)	-5.479	1.861	-0.876	H(31)	-5.934	0.603	0.878
H(32)	-6.617	0.827	-0.000	H(32)	-5.934	0.603	-0.879
C(33)	-4.409	-3.559	-0.000	H(33)	-6.699	-0.725	-0.000
H(34)	-4.039	-4.067	-0.887	C(34)	-3.396	-4.314	-0.000
H(35)	-4.039	-4.067	0.887	H(35)	-2.884	-4.682	-0.885
H(36)	-5.494	-3.573	-0.000	H(36)	-2.884	-4.682	0.885
C(37)	6.314	-2.070	-0.000	H(37)	-4.426	-4.655	-0.000
H(38)	6.429	-2.704	0.879	C(38)	6.421	-1.843	-0.000
H(39)	6.429	-2.705	-0.879	H(39)	6.567	-2.471	0.879
H(40)	7.132	-1.352	-0.000	H(40)	6.566	-2.472	-0.880
S(41)	-1.583	-3.192	-0.000	H(41)	7.203	-1.087	-0.001
O(42)	-2.887	1.670	0.000	S(42)	-3.361	1.574	-0.000

Table S5. Cartesian coordinates of the optimized (PBE0) Hg(II)-mediated base pairs between 1-mercuri-3-methylcarbazole and either 1-methyl-2-thiothymine or 1-methyl-4-thiothymine.

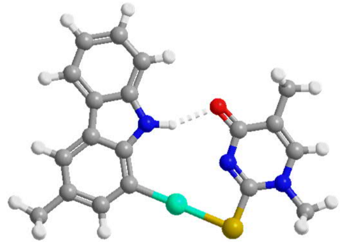

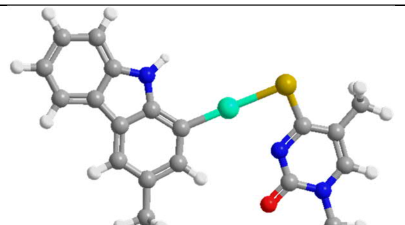

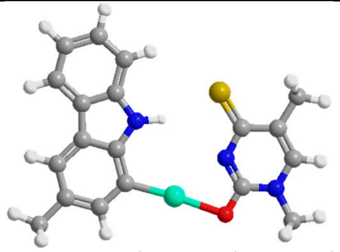

Atom	X / Å	Y / Å	Z / Å	Atom	X / Å	Y / Å	Z / Å	Atom	X / Å	Y / Å	Z / Å
C(1)	4.908	3.547	0.001	C(1)	-7.154	-0.261	-0.000	C(1)	5.036	3.443	0.001
C(2)	3.663	4.198	0.001	C(2)	-6.790	-1.616	-0.001	C(2)	3.807	4.123	0.001
C(3)	2.472	3.486	0.001	C(3)	-5.458	-2.008	-0.001	C(3)	2.599	3.441	0.000
C(4)	2.556	2.093	0.000	C(4)	-4.490	-1.005	-0.000	C(4)	2.648	2.046	0.000
C(5)	3.800	1.420	0.000	C(5)	-4.836	0.368	0.000	C(5)	3.876	1.344	0.001
C(6)	4.985	2.162	0.000	C(6)	-6.185	0.733	-0.000	C(6)	5.079	2.057	0.001
H(7)	5.820	4.138	0.001	H(7)	-8.205	0.010	-0.000	H(7)	5.961	4.011	0.001
H(8)	3.631	5.284	0.001	H(8)	-7.566	-2.377	-0.001	H(8)	3.801	5.209	0.001
H(9)	5.950	1.663	0.000	H(9)	-6.471	1.781	0.000	H(9)	6.031	1.533	0.001
C(10)	3.497	0.010	0.000	C(10)	-3.596	1.108	0.000	C(10)	3.542	-0.059	0.000
C(11)	4.288	-1.140	0.000	C(11)	-3.282	2.469	0.000	C(11)	4.305	-1.228	0.001
C(12)	2.081	-0.101	0.000	C(12)	-2.552	0.153	0.000	C(12)	2.124	-0.138	0.000
C(13)	3.695	-2.398	-0.001	C(13)	-1.953	2.879	0.000	C(13)	3.685	-2.473	0.000
H(14)	5.372	-1.054	0.000	H(14)	-4.077	3.212	0.001	H(14)	5.391	-1.167	0.001
C(15)	1.466	-1.362	0.000	C(15)	-1.209	0.537	0.000	C(15)	1.486	-1.385	0.000
C(16)	2.289	-2.486	-0.001	C(16)	-0.934	1.904	0.000	C(16)	2.278	-2.531	0.000
H(17)	1.844	-3.478	-0.001	H(17)	0.100	2.244	0.000	H(17)	1.807	-3.511	0.000
N(18)	1.538	1.164	0.000	N(18)	-3.115	-1.108	0.000	N(18)	1.609	1.139	0.000
H(19)	0.555	1.435	0.000	H(19)	-2.597	-1.971	-0.001	H(19)	0.629	1.418	0.000
Hg(20)	-0.586	-1.540	0.000	Hg(20)	0.396	-0.761	0.000	Hg(20)	-0.552	-1.475	0.000
C(21)	-4.473	1.928	0.000	C(21)	5.537	0.125	-0.000	C(21)	-4.896	1.026	0.000
C(22)	-3.163	-0.026	0.000	C(22)	3.555	1.497	-0.000	C(22)	-3.128	-0.517	0.000
C(23)	-2.069	2.062	-0.001	C(23)	3.397	-0.853	0.000	C(23)	-2.611	1.782	0.000
C(24)	-3.376	2.719	-0.001	C(24)	4.816	-1.034	0.000	C(24)	-4.037	2.076	0.000
H(25)	-5.479	2.334	0.000	H(25)	6.624	0.118	-0.000	H(25)	-5.973	1.159	0.000
N(26)	-2.060	0.683	0.000	N(26)	2.824	0.340	-0.000	N(26)	-2.248	0.471	0.000
N(27)	-4.400	0.550	0.000	N(27)	4.957	1.349	-0.000	N(27)	-4.470	-0.283	0.000
C(28)	-3.436	4.209	-0.001	C(28)	5.463	-2.383	0.000	C(28)	-4.530	3.486	0.000
H(29)	-2.917	4.611	0.875	H(29)	5.169	-2.964	0.881	H(29)	-4.153	4.024	0.875
H(30)	-2.917	4.610	-0.878	H(30)	5.169	-2.964	-0.880	H(30)	-4.153	4.023	-0.876
H(31)	-4.467	4.573	-0.001	H(31)	6.553	-2.293	-0.000	H(31)	-5.623	3.525	0.000
C(32)	-5.613	-0.256	0.001	C(32)	5.740	2.574	-0.001	C(32)	-5.396	-1.404	0.000
H(33)	-5.649	-0.891	-0.888	H(33)	5.498	3.169	-0.884	H(33)	-5.242	-2.023	-0.886
H(34)	-5.649	-0.890	0.890	H(34)	5.498	3.170	0.882	H(34)	-5.241	-2.023	0.887
H(35)	-6.474	0.413	0.001	H(35)	6.801	2.319	-0.000	H(35)	-6.415	-1.015	0.000
C(36)	4.524	-3.653	-0.001	C(36)	-1.585	4.337	0.001	C(36)	4.486	-3.746	0.000
H(37)	4.320	-4.272	0.880	H(37)	-0.987	4.598	0.880	H(37)	4.268	-4.359	0.882
H(38)	4.320	-4.271	-0.883	H(38)	-0.987	4.598	-0.880	H(38)	4.269	-4.359	-0.881
H(39)	5.593	-3.421	-0.001	H(39)	-2.476	4.972	0.001	H(39)	5.559	-3.536	0.001
S(40)	-3.035	-1.769	0.001	H(40)	-5.183	-3.059	-0.001	H(40)	1.649	3.966	0.000
O(41)	-1.006	2.690	-0.001	S(41)	2.335	-2.240	0.000	S(41)	-1.441	2.981	-0.001
H(42)	1.506	3.983	0.001	O(42)	3.069	2.620	-0.001	O(42)	-2.711	-1.735	0.000

Table S6. Cartesian coordinates of the optimized (PBE0) Hg(II)-mediated base pairs between 8-mercuri-3-methylcarbazole and either 1-methyl-2-thiothymine or 1-methyl-4-thiothymine.

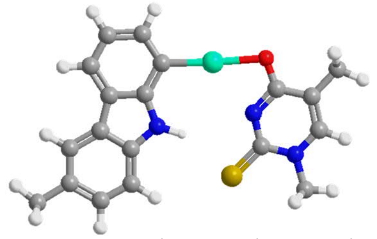

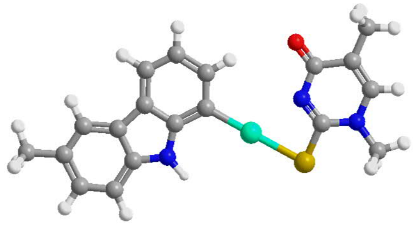

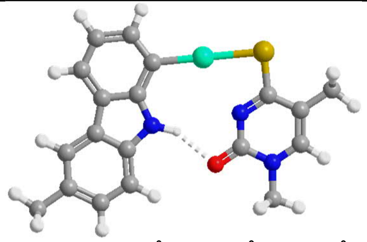

Atom	X / Å	Y / Å	Z / Å	Atom	X / Å	Y / Å	Z / Å	Atom	X / Å	Y / Å	Z / Å
C(1)	-2.864	-3.517	0.000	C(1)	1.711	3.036	-0.001	C(1)	-2.783	-3.542	-0.001
C(2)	-1.486	-3.266	0.000	C(2)	0.681	2.082	-0.002	C(2)	-1.406	-3.274	-0.001
C(3)	-0.985	-1.964	0.000	C(3)	0.963	0.714	-0.001	C(3)	-0.911	-1.970	0.000
C(4)	-1.904	-0.905	0.000	C(4)	2.306	0.334	-0.000	C(4)	-1.847	-0.924	0.000
C(5)	-3.306	-1.156	0.000	C(5)	3.356	1.288	-0.000	C(5)	-3.246	-1.187	0.000
C(6)	-3.775	-2.469	0.000	C(6)	3.043	2.648	-0.001	C(6)	-3.704	-2.504	-0.001
H(7)	-3.217	-4.544	-0.001	H(7)	1.452	4.090	-0.002	H(7)	-3.124	-4.573	-0.001
H(8)	-0.802	-4.109	0.000	H(8)	-0.352	2.423	-0.002	H(8)	-0.717	-4.114	-0.001
H(9)	-4.843	-2.668	-0.001	H(9)	3.834	3.393	-0.001	H(9)	-4.770	-2.713	-0.001
C(10)	-3.954	0.133	0.000	C(10)	4.595	0.544	0.000	C(10)	-3.908	0.095	0.000
C(11)	-5.290	0.546	0.000	C(11)	5.944	0.908	0.001	C(11)	-5.247	0.498	0.000
C(12)	-2.924	1.097	0.000	C(12)	4.245	-0.824	0.000	C(12)	-2.886	1.069	0.001
C(13)	-5.595	1.902	0.000	C(13)	6.930	-0.073	0.001	C(13)	-5.563	1.852	0.000
H(14)	-6.091	-0.189	-0.001	H(14)	6.226	1.958	0.001	H(14)	-6.043	-0.243	0.000
C(15)	-3.208	2.463	0.001	C(15)	5.216	-1.824	0.001	C(15)	-3.180	2.433	0.001
C(16)	-4.541	2.840	0.000	C(16)	6.546	-1.429	0.001	C(16)	-4.517	2.799	0.001
H(17)	-4.787	3.900	0.000	H(17)	7.321	-2.192	0.001	H(17)	-4.773	3.856	0.001
N(18)	-1.701	0.455	0.001	N(18)	2.867	-0.927	0.000	N(18)	-1.659	0.440	0.001
H(19)	-0.813	0.951	0.001	H(19)	2.348	-1.788	-0.002	H(19)	-0.781	0.954	0.000
Hg(20)	1.017	-1.569	0.000	Hg(20)	-0.632	-0.594	-0.001	Hg(20)	1.113	-1.584	0.000
C(21)	4.603	1.973	0.000	C(21)	-5.747	0.177	0.001	C(21)	3.882	2.866	0.000
C(22)	2.215	2.118	0.000	C(22)	-3.587	-0.755	0.000	C(22)	1.551	2.301	-0.001
C(23)	3.291	0.022	0.000	C(23)	-3.792	1.601	0.001	C(23)	3.187	0.614	0.000
C(24)	4.598	0.613	0.000	C(24)	-5.251	1.432	0.001	C(24)	4.266	1.556	0.000
H(25)	5.532	2.535	0.000	H(25)	-6.813	-0.029	0.000	H(25)	4.610	3.672	0.000
N(26)	2.190	0.773	0.000	N(26)	-3.037	0.435	0.000	N(26)	1.917	0.991	-0.001
N(27)	3.466	2.727	0.000	N(27)	-4.937	-0.944	-0.000	N(27)	2.580	3.251	-0.001
C(28)	5.833	-0.227	-0.001	C(28)	-6.109	2.652	0.001	C(28)	5.703	1.141	0.001
H(29)	5.860	-0.879	0.878	H(29)	-5.889	3.271	0.877	H(29)	5.938	0.534	0.881
H(30)	5.860	-0.879	-0.879	H(30)	-5.889	3.271	-0.874	H(30)	5.939	0.534	-0.879
H(31)	6.734	0.393	-0.001	H(31)	-7.173	2.400	0.001	H(31)	6.363	2.013	0.001
C(32)	3.557	4.180	0.000	C(32)	-5.520	-2.278	0.000	C(32)	2.188	4.651	-0.001
H(33)	3.057	4.585	-0.883	H(33)	-5.204	-2.834	-0.886	H(33)	1.584	4.867	-0.885
H(34)	3.057	4.585	0.883	H(34)	-5.212	-2.830	0.892	H(34)	1.584	4.867	0.883
H(35)	4.610	4.464	0.000	H(35)	-6.606	-2.182	-0.005	H(35)	3.085	5.272	-0.001
C(36)	-7.022	2.378	-0.001	C(36)	8.390	0.293	0.001	C(36)	-6.994	2.316	0.000
H(37)	-7.241	2.992	0.881	H(37)	8.882	-0.132	0.916	H(37)	-7.219	2.927	0.882
H(38)	-7.240	2.992	-0.882	H(38)	8.881	-0.125	-0.917	H(38)	-7.218	2.928	-0.881
H(39)	-7.722	1.537	-0.001	H(39)	8.496	1.410	0.003	H(39)	-7.686	1.469	0.000
S(40)	0.805	3.032	0.000	S(40)	-2.532	-2.153	0.000	H(40)	-2.386	3.172	0.001
O(41)	3.138	-1.260	0.000	O(41)	-3.255	2.705	0.001	O(41)	0.377	2.673	-0.001
H(42)	-2.412	3.201	0.001	H(42)	4.942	-2.875	-0.009	S(42)	3.510	-1.098	0.001

Table S7. Cartesian coordinates of the optimized (PBE0) Hg(II)-mediated base pairs between 1,8-dimercuri-3-methylcarbazole and either 1-methyl-2-thiothymine or 1-methyl-4-thiothymine.

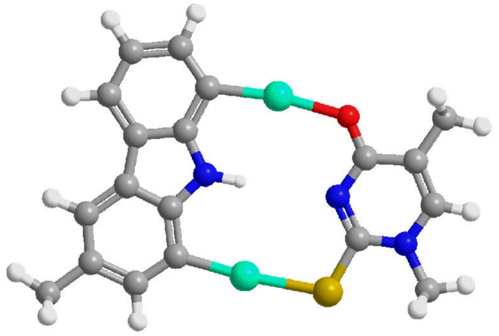

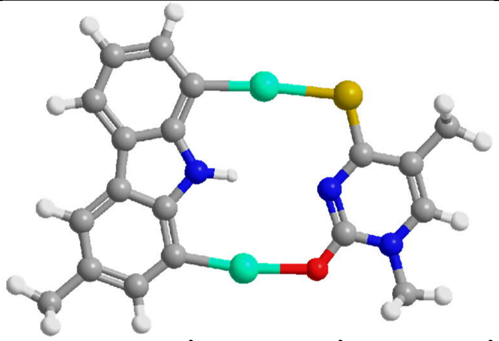

Atom	X / Å	Y / Å	Z / Å	Atom	X / Å	Y / Å	Z / Å
C(1)	2.956	4.428	0.000	C(1)	2.826	4.473	0.000
C(2)	1.554	4.330	0.000	C(2)	1.428	4.326	0.000
C(3)	0.953	3.078	0.000	C(3)	0.855	3.058	0.000
C(4)	1.779	1.956	0.000	C(4)	1.727	1.972	0.000
C(5)	3.192	2.031	0.000	C(5)	3.136	2.089	0.000
C(6)	3.774	3.302	0.000	C(6)	3.681	3.377	0.000
H(7)	3.408	5.415	0.000	H(7)	3.244	5.475	0.000
H(8)	0.959	5.238	0.000	H(8)	0.807	5.216	0.000
H(9)	4.853	3.417	0.000	H(9)	4.756	3.525	0.000
C(10)	3.672	0.659	0.000	C(10)	3.660	0.734	0.000
C(11)	4.921	0.032	0.000	C(11)	4.929	0.147	0.000
C(12)	2.524	-0.161	0.000	C(12)	2.537	-0.121	0.000
C(13)	5.010	-1.360	0.000	C(13)	5.068	-1.242	0.000
H(14)	5.833	0.624	0.000	H(14)	5.820	0.769	0.000
C(15)	2.575	-1.551	0.000	C(15)	2.651	-1.508	0.000
C(16)	3.833	-2.142	0.000	C(16)	3.922	-2.066	0.000
H(17)	3.935	-3.224	0.000	H(17)	4.055	-3.144	0.000
N(18)	1.402	0.636	0.000	N(18)	1.392	0.637	0.000
H(19)	0.451	0.298	0.000	H(19)	0.449	0.278	0.000
Hg(20)	-1.014	2.540	0.000	Hg(20)	-1.130	2.489	0.000
Hg(21)	0.701	-2.412	0.000	Hg(21)	0.816	-2.395	0.000
C(22)	-4.940	-1.200	0.000	C(22)	-4.582	-2.140	0.000
C(23)	-2.663	-1.831	0.000	C(23)	-2.225	-2.197	0.000
C(24)	-3.218	0.423	0.000	C(24)	-3.301	-0.145	0.000
C(25)	-4.622	0.119	0.000	C(25)	-4.590	-0.777	0.000
H(26)	-5.968	-1.545	0.000	H(26)	-5.497	-2.722	0.000
N(27)	-2.283	-0.556	0.000	O(27)	-1.204	-2.973	0.000
N(28)	-3.989	-2.180	0.000	N(28)	-2.163	-0.859	0.000
C(29)	-5.640	1.209	0.000	N(29)	-3.427	-2.855	0.000
H(30)	-5.518	1.849	0.879	C(30)	-5.879	-0.017	0.000
H(31)	-5.518	1.849	-0.878	H(31)	-5.956	0.629	0.881
H(32)	-6.654	0.804	0.000	H(32)	-5.956	0.629	-0.881
C(33)	-4.421	-3.581	0.000	H(33)	-6.731	-0.700	0.000
H(34)	-4.047	-4.091	-0.890	C(34)	-3.439	-4.317	0.000
H(35)	-4.047	-4.091	0.890	H(35)	-2.927	-4.692	-0.888
H(36)	-5.510	-3.603	0.000	H(36)	-2.927	-4.691	0.888
C(37)	6.348	-2.048	0.000	H(37)	-4.474	-4.655	0.000
H(38)	6.468	-2.685	0.882	C(38)	6.431	-1.881	0.000
H(39)	6.468	-2.686	-0.882	H(39)	6.574	-2.513	0.882
H(40)	7.166	-1.324	0.000	H(40)	6.574	-2.514	-0.882
S(41)	-1.586	-3.204	0.000	H(41)	7.221	-1.127	0.000
O(42)	-2.912	1.669	0.000	S(42)	-3.367	1.590	0.000

Table S8. Calculated energies of the Hg(II)-mediated base pairs.

Base pair	*E*(PBE0DH) / Hartree	*ΔE*(PBE0DH) / kJ mol^{-1}	*E*(PBE0) / Hartree	*ΔE*(PBE0) / kJ mol^{-1}
1-Mercuri-3-methylcarbazole—1-methyl-2-thiothymine	-1524.046894	22.74	-1524.231387	23.23
1-Mercuri-3-methylcarbazole—1-methyl-4-thiothymine (Hg—S)	-1524.045077	27.51	-1524.230250	26.22
1-Mercuri-3-methylcarbazole—1-methyl-4-thiothymine (Hg—O)	-1524.026946	75.12	-1524.212045	74.02
8-Mercuri-3-methylcarbazole—1-methyl-4-thiothymine	-1524.055556	0	-1524.240236	0
8-Mercuri-3-methylcarbazole—1-methyl-2-thiothymine (Hg—S)	-1524.034968	54.05	-1524.219922	53.33
8-Mercuri-3-methylcarbazole—1-methyl-2-thiothymine (Hg—O)	-1524.029700	67.89	-1524.214998	66.26
1,8-Dimercuri-3-methylcarbazole—1-methyl-2-thiothymine	-1676.610042	11.54	-1676.882740	11.46
1,8-Dimercuri-3-methylcarbazole—1-methyl-4-thiothymine	-1676.614439	0	-1676.887104	0
